# Transitional Care Interventions From Hospital to Community to Reduce Health Care Use and Improve Patient Outcomes: A Systematic Review and Network Meta-Analysis

**DOI:** 10.1001/jamanetworkopen.2023.44825

**Published:** 2023-11-30

**Authors:** Natasha Tyler, Alexander Hodkinson, Claire Planner, Ioannis Angelakis, Christopher Keyworth, Alex Hall, Paul Pascall Jones, Oliver George Wright, Richard Keers, Tom Blakeman, Maria Panagioti

**Affiliations:** 1 National Institute for Health Research School for Primary Care Research, Division of Population Health, Health Services Research and Primary Care, School of Health Sciences, Faculty of Biology, Medicine and Health, University of Manchester, Manchester Academic Health Science Centre, University of Manchester, Manchester, United Kingdom; 2 National Institute for Health and Care Research Greater Manchester Patient Safety Translational Research Centre, Division of Population Health, Health Services Research & Primary Care, University of Manchester, Manchester, United Kingdom; 3 Institute of Population Health, Department of Primary Care & Mental Health, University of Liverpool, Liverpool, United Kingdom; 4 School of Psychology, University of Leeds, Leeds, United Kingdom; 5 Division of Nursing, Midwifery & Social Work, University of Manchester, Manchester, United Kingdom; 6 University of Manchester, Manchester, United Kingdom; 7 Pharmacy Department, Pennine Care NHS Foundation Trust, Aston-Under-Lyne, United Kingdom; 8 Centre for Pharmacoepidemiology and Drug Safety, Division of Pharmacy and Optometry, School of Health Sciences, University of Manchester, Manchester, United Kingdom

## Abstract

**Importance:**

Discharge from the hospital to the community has been associated with serious patient risks and excess service costs.

**Objective:**

To evaluate the comparative effectiveness associated with transitional care interventions with different complexity levels at improving health care utilization and patient outcomes in the transition from the hospital to the community.

**Data Sources:**

CENTRAL, Embase, MEDLINE, and PsycINFO were searched from inception until August 2022.

**Study Selection:**

Randomized clinical trials evaluating transitional care interventions from hospitals to the community were identified.

**Data Extraction and Synthesis:**

At least 2 reviewers were involved in all data screening and extraction. Random-effects network meta-analyses and meta-regressions were applied. The Preferred Reporting Items for Systematic Reviews and Meta-analyses (PRISMA) guidelines were followed.

**Main Outcomes and Measures:**

The primary outcomes were readmission at 30, 90, and 180 days after discharge. Secondary outcomes included emergency department visits, mortality, quality of life, patient satisfaction, medication adherence, length of stay, primary care and outpatient visits, and intervention uptake.

**Results:**

Overall, 126 trials with 97 408 participants were included, 86 (68%) of which were of low risk of bias. Low-complexity interventions were associated with the most efficacy for reducing hospital readmissions at 30 days (odds ratio [OR], 0.78; 95% CI, 0.66 to 0.92) and 180 days (OR, 0.45; 95% CI, 0.30 to 0.66) and emergency department visits (OR, 0.68; 95% CI, 0.48 to 0.96). Medium-complexity interventions were associated with the most efficacy at reducing hospital readmissions at 90 days (OR, 0.64; 95% CI, 0.45 to 0.92), reducing adverse events (OR, 0.42; 95% CI, 0.24 to 0.75), and improving medication adherence (standardized mean difference [SMD], 0.49; 95% CI, 0.30 to 0.67) but were associated with less efficacy than low-complexity interventions for reducing readmissions at 30 and 180 days. High-complexity interventions were most effective for reducing length of hospital stay (SMD, −0.20; 95% CI, −0.38 to −0.03) and increasing patient satisfaction (SMD, 0.52; 95% CI, 0.22 to 0.82) but were least effective for reducing readmissions at all time periods. None of the interventions were associated with improved uptake, quality of life (general, mental, or physical), or primary care and outpatient visits.

**Conclusions and Relevance:**

These findings suggest that low- and medium-complexity transitional care interventions were associated with reducing health care utilization for patients transitioning from hospitals to the community. Comprehensive and consistent outcome measures are needed to capture the patient benefits of transitional care interventions.

## Key Points

**Question:**

What is the comparative effectiveness associated with transitional care interventions with different complexity levels in improving health care utilization and patient outcomes after hospital discharge?

**Findings:**

In this systematic review and network meta-analysis including 126 trials with 97 408 participants, low- and medium-complexity interventions were associated with decreased odds of readmission at 30 days compared with usual care. All intervention complexities were associated with significant reductions in the odds of readmissions at 180 days.

**Meaning:**

These findings suggest that low- and medium-complexity transitional care interventions may be more effective for reducing readmission for patients transitioning from hospitals to the community.

## Teaser

This systematic review and network meta-analysis evaluates the comparative effectiveness associated with transitional care interventions with different complexity levels at improving health care utilization and patient outcomes in the transition from hospitals to the community.

## Introduction

An increased demand for urgent hospital care has created pressure to discharge patients to the community,^1^ with some patients being discharged too early or without necessary support to recover in the community. especially during the COVID-19 pandemic.^2,3^ There is evidence that 1 in 5 patients may experience suboptimal or unsafe care around the time of discharge from a hospital, mainly because of the prompt reduction in continuity of care and coordination challenges of multiple independent professionals and agencies.^1,4,5^

To date, several trials have evaluated transitional care interventions to improve health care utilization and patient outcomes in the transition from hospitals to the community. Some interventions include multiple components,^6^ implemented mainly prior to discharge but some also after discharge,^7,8^ and/or involve a care coordinator or case manager.^9,10^ Other less-intensive interventions target 1 key challenge of the discharge process (eg, medication safety)^11,12^ at 1 stage of the discharge process.^13,14^ A number of systematic reviews suggest that various transitional care interventions are promising for improving health care utilization and possibly patient outcomes.^15,16,17,18^ However, most of these systematic reviews have focused on certain health care settings or populations or have included data from varying study designs that cannot be pooled together.^19,20^ Hence, despite the large number of trials conducted to improve discharge from hospitals to the community, there is no definitive evidence on how intensive (in terms of the number of components and number of discharge stages) transitional care interventions must be to work best, and whether different intervention complexity levels are best for improving certain outcomes.

This systematic review and network meta-analysis^21^ examined the comparative effectiveness and uptake associated with different intensities of transitional care interventions in improving health care utilization and patient outcomes in the transition from the hospital to the community.

## Methods

This systematic review and network meta-analysis is reported following the Preferred Reporting Items for Systematic Reviews and Meta-analyses Extension Statement for Reporting of Systematic Reviews Incorporating Network Meta-analyses of Health Care Interventions (PRISMA-NMA) reporting guideline.^22^ The review protocol is registered on PROSPERO (record No. CRD42020166169).

### Patient and Public Involvement

We regularly consulted a group of 4 patient and carer partners who were members of an established patient and public involvement group about the appropriateness of our research questions, development of the review protocol, classification of the complexity levels of transitional care interventions, and selection of the outcome measures of this study. Patient and carer partners also advised on the interpretation of our findings, and their dissemination including drafting lay summaries.

### Search Methods

Searches were performed in the Cochrane Central Register of Controlled Trials, CINAHL, Embase, MEDLINE, and PsycINFO from inception until August 2022, with no language restriction. We used combinations of Medical Subject Headings terms and text words in *discharge*, *intervention*, *readmission*, *continuity of patient care*. The full search strategy for each database is available in eAppendix 1 in Supplement 1. The search strategy was adapted from a Cochrane discharge planning from hospital review^19^ and the reference lists of 2 relevant reviews were screened.^19,20^

### Eligibility Criteria

#### Population

All patients in hospitals (acute, rehabilitation, or community) were eligible. Patients of any age, sex, or condition were eligible.

#### Intervention

Inclusion criteria were randomized clinical trials (RCTs) or cluster RCTs evaluating an intervention for transitional care from hospitals to the community, implemented prior to discharge (discharge planning), after discharge, or across the discharge period (before, bridging, and after). We excluded studies whereby the transitional or discharge element was a minor component of a multifaceted intervention. We also excluded studies that were solely about follow-up in the community without a discharge component.

#### Comparator

Any comparator was eligible for inclusion. Comparators included usual care, another intervention, minimal intervention, or no intervention.

### Outcome

The primary outcomes were readmission at 30, 90, and 180 days after discharge. Secondary outcomes included emergency department (ED) visits, mortality, quality of life (QOL), patient satisfaction, medication adherence, length of stay, primary care and outpatient visits, and intervention uptake.

### Data Collection and Extraction

A 3-stage screening was conducted. Title screening was conducted by 2 authors independently (N.T. and M.P.). One author screened 100% of abstracts (N.T.) and 4 authors double-screened 25% of abstracts each, independently (A. Hodkinson, C.K., and A. Hall). One author screened 100% of full texts (N.T.) and 2 authors double-screened 50% each, independently (C.K., A. Hall).

Quantitative data were extracted by 1 author (N.T.) and checked for consistency by 2 authors (A. Hodkinson and M.P.). Descriptive data were extracted by 2 authors (P.P.J. and O.G.W.) and checked for consistency by 2 authors (C.K. and A. Hall). A novel data extraction Excel spreadsheet version 1 (Microsoft) was used that was based on the Cochrane data extraction spreadsheet but refined for the needs of this study. The spreadsheet was piloted on 5 studies and adapted after discussion among 3 authors (N.T., A. Hodkinson, and M.P.).

### Classification of Intervention Complexity

**Figure 1.**
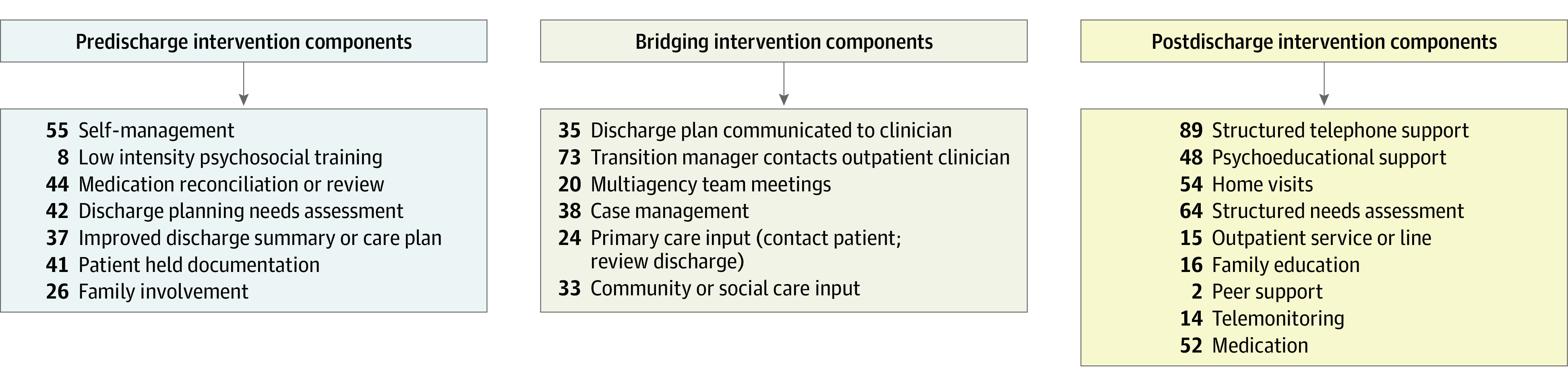
Transitional Care Components Per Discharge Phase

For determining the complexity of the interventions, we focused on the number of key transitional care components included, as well as the number of discharge stages (before discharge, after discharge, or bridging) at which the components were implemented. These components were themed after reviewing previous systematic reviews of transitional care interventions,^23,24,25,26,27,28^ consultations within our research team, and professionals and patients with lived experience of transitioning from hospitals to the community. The transitional care components per discharge phase are presented in Figure 1. Interventions that included 8 or more of these components were classified as high complexity, those with 4 to 7 components, medium complexity, and those with 1 to 3 components, low complexity.

### Assessment of Risk of Bias

We used 4 risk of bias (ROB) criteria from the Cochrane Risk of Bias Tool for RCTs: allocation concealment (range, 1-3; 3 indicates adequate; 2, less adequate; 1, unclear), intention-to-treat (range, 1-3; 3 indicates yes; 2, no; 1, unclear), attrition (range, 0-2; 2 indicates low [<5%]; 1, medium [5%-20%]; 0, high or unclear [>20%]) and selection reporting bias (range, 0-2; 2 indicates low; 1, medium; 0, unclear); we excluded blinding because it was used in service-level interventions. A total ROB score was calculated for each study, which ranged from 2 to 10. Scores greater than 6 were classified as low ROB overall and scores of 6 of less were classified as high ROB overall.

### Missing Data

Study authors were contacted (3 attempts were made) where there was missing or unclear data (eg, relating to the primary outcomes). Studies for which sufficient primary data were not obtained were excluded.

### Statistical Analysis

#### Network Meta-Analyses of Primary Outcomes

We conducted network meta-analyses on the complexity of interventions (low, medium, high), including minimal control interventions, to reduce readmission at 30, 90, and 180 days after discharge; ED visits; mortality; and intervention uptake compared with usual care (UC) at the level of significance of α = .05. We conducted pairwise meta-analyses using Dersimonian Laird random effects on the complexity of interventions to reduce adverse events, patient safety incidents, medication adherence, length of hospital stay, general QOL, and patient satisfaction at the level of significance of α = .05. We converted the dichotomous outcome data to log odds ratios (ORs) and then back to ORs. Continuous data were converted to the standardized mean difference (SMD), and pooled effect sizes were interpreted according to Cohen criteria.^16^ The Comprehensive Meta-Analysis version 3 (Biostat) was used to perform the transformations.

Random-effects network meta-analysis models were based on the frequentist package *netmeta* in R version 4.0.5 (R Project for Statistical Computing). Network graphs scaled by the number of studies and forest plots presented by each intervention complexity compared with no intervention or UC, were presented. League tables of all head-to-head comparisons of interventions were also inspected (eAppendix 2 in Supplement 1). The *I^2^* statistic and the heterogeneity variance in the random effect’s distribution (τ^2^) were used to measure the extent of the influence of variability across and within studies on intervention effects. Traditionally, values of 25% indicate low heterogeneity; 50%, moderate heterogeneity; and 75%, high heterogeneity. We considered the *P*-score, a frequentist analogue to surface under the cumulative ranking,^18^ to rank the interventions’ performance. We separated direct from indirect evidence by use of node splitting to evaluate consistency.^19^ Cochrane *Q* statistic was used to calculate consistency throughout the entire network.^19^ We produced network funnel plots to examine the presence of bias due to small-study effect, which allowed us to visually scrutinize the criterion of symmetry. A sensitivity network meta-analysis for 30- and 90-day readmissions was conducted based on the number of discharge stages (1 to 3) and their 7 combinations (before, after, bridging, before to after, before and bridging, after and bridging, and before, after, and bridging).

#### Meta-Regressions

A series of univariate network meta-regressions were conducted for readmissions at 30 and 90 days, intervention uptake, and mortality, with a level of significance of α = .05. All models were fitted in OpenBUGS version 3.2.3 (MRC Biostatistics Unit, University of Cambridge) using uninformative prior distributions for the intervention effects and a minimally informative prior distribution for common heterogeneity SD. We assumed uninformative priors for all meta-regression coefficients. Model convergence was ensured by visual inspection of the 3 Markov Chain Monte Carlo chains after considering the Brooks Gelman Rubin diagnostic. Overall, 8 factors were examined, including age (<45, 45 to 59, 60 to 79, ≥80 years), sex (studies involving 54% or more females, studies involving 54% or more males, mixed or not reported), Organization for Economic Cooperation and Development (OECD) (no, yes, or not reported), World Health Organization (WHO) region (Africa, America, Southeast Asia, Europe, Eastern Mediterranean, Western Pacific, or unclear), delivery professional (nurse, pharmacist, medic, care coordinator, multidisciplinary team, 2 professionals, allied health professionals, nonclinical staff, social worker, or not reported), medication reconciliation (no, yes, or not reported), patient population (medical or mental health), ROB, and patient complexity (low vs high, based on studies that explicitly reported the population as high risk, high complexity or described multimorbidity, polypharmacy, vulnerability, and terminal illness).

## Results

**Figure 2.**
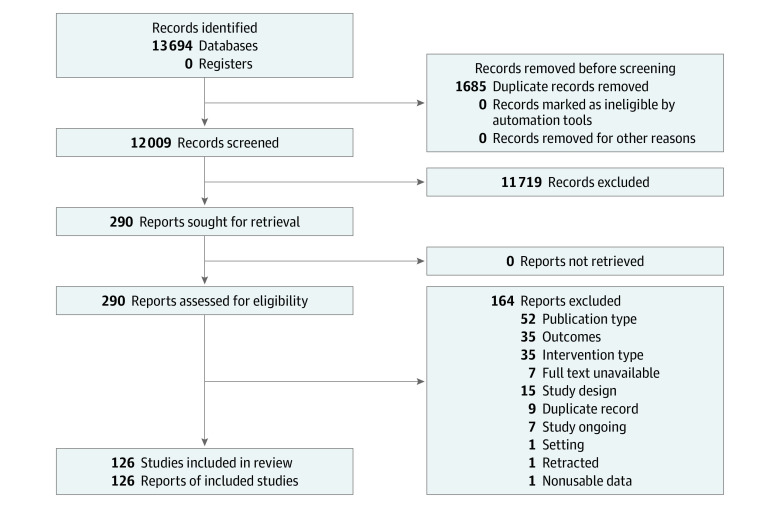
Flowchart of Study Selection

After removing duplicates, the search retrieved 10 685 references. Following title and abstract screening, 274 full texts were retrieved. A total of 126 RCTs^7,8,10,11,12,13,14,29,30,31,32,33,34,35,36,37,38,39,40,41,42,43,44,45,46,47,48,49,50,51,52,53,54,55,56,57,58,59,60,61,62,63,64,65,66,67,68,69,70,71,72,73,74,75,76,77,78,79,80,81,82,83,84,85,86,87,88,89,90,91,92,93,94,95,96,97,98,99,100,101,102,103,104,105,106,107,108,109,110,111,112,113,114,115,116,117,118,119,120,121,122,123,124,125,126,127,128,129,130,131,132,133,134,135,136,137,138,139,140,141,142,143,144,145,146,147,148^ involving 97 408 participants met our inclusion criteria (Figure 2). The study characteristics are presented in eAppendix 3 in Supplement 1.

### Descriptive Characteristics of the Included Studies

Most studies were conducted in OECD countries (88 studies [70%]); 51 studies (40%) were conducted in the Americas, 35 studies (28%) in Europe, 28 studies (22%) in the Western Pacific, 8 studies (6%) in the Eastern Mediterranean, 3 studies (2%) in Africa, and 1 study (1%) in South-East Asia. Forty-two studies (33%) included mostly female participants, 42 studies (33%) included mostly male participants, and 37 studies (29%) included an equal percentage of male and female participants. The mean age of the participants ranged between 2 and 87 years (median [IQR], 66 [59-75] years). Nine studies (7%) were conducted in mental health hospitals, and the remaining 117 studies (93%) were conducted in general hospitals. There were 56 studies (44%) that did not use a condition reporting index, 21 studies (17%) that used the Charlson Comorbidity Index, 10 studies (8%) that used the New York Heart Association functional classification, and 39 studies (33%) that used another reporting index (eAppendix 3 in Supplement 1).

In assessment of study interventions, 37 studies (29%) applied low-complexity interventions, 41 studies (33%) used medium-complexity interventions, and 48 studies (38%) used high-complexity interventions. In terms of discharge stage, 49 studies (38%) applied intervention elements across all 3 stages (before discharge, after discharge, and bridging), 49 studies (39%) applied intervention elements in 2 stages, and 28 studies (22%) applied the intervention in 1 stage only. We found 45 studies (36%) that included a medication reconciliation component. In 45 studies (36%), interventions were conducted by a nurse, 34 studies (27%) had interventions conducted by another health professional, 29 studies (23%) used a multidisciplinary team, 8 studies (6%) had interventions conducted by social care professionals, and 10 studies (8%) had interventions conducted by others.

### Assessment of Risk of Bias

In ROB analysis, 86 studies (68%) were of low ROB, whereas 40 studies (32%) showed high ROB. Ratings for each of the ROB domains are provided in eAppendix 4 in Supplement 1.

### Network Meta-Analysis

#### 30-Day Hospital Readmissions

**Figure 3.**
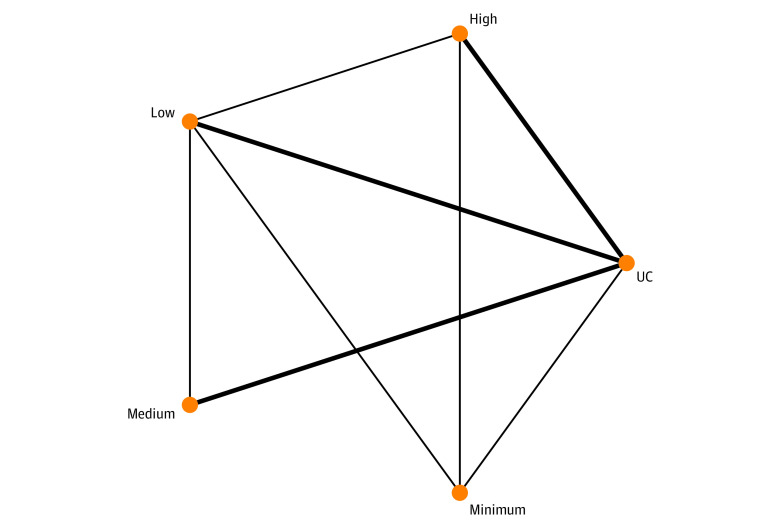
Network Meta-Analysis of Association of Discharge Intervention Intensities With Reducing 30-Day Readmissions Minimum indicates minimal intervention; UC, usual care. Line thickness indicates number of included studies.

Pooling data from the 73 studies^7,8,11,12,13,14,29,30,31,34,35,36,37,38,39,43,45,46,47,48,49,50,51,52,53,54,56,58,59,60,63,67,68,69,70,73,76,77,79,80,81,85,86,87,88,89,90,94,95,96,97,98,102,104,106,111,112,113,114,115,116,119,121,125,127,128,129,131,133,135,141,146,147^ (85 direct comparisons) involving 77 201 participants, low-complexity (OR, 0.78; 95% CI, 0.66 to 0.92) and medium-complexity (OR, 0.82; 95% CI, 0.68 to 0.97) interventions were associated with decreased odds of readmission at 30 days compared with usual care (Figure 3; eAppendix 2 in Supplement 1). High-intensity interventions were not associated with reductions in readmissions (OR, 0.96; 95% CI, 0.80 to 1.15). The *P*-score also showed that low-complexity interventions (*P*-Score, 89%) were associated with the most efficacy in reducing the 30-day readmission odds. Global heterogeneity of the network was seen to be moderate (*I^2^* = 65%; 95% CI, 53% to 71%). There was evidence of inconsistency through node-splitting analysis in the comparison of high-complexity interventions against minimal interventions (ratio OR, 2.96; 95% CI, 1.20 to 7.29) and the comparison of minimal interventions against usual care (ratio OR, 4.29; 95% CI, 1.80 to 10.18) (eAppendix 5 in Supplement 1). There was evidence of publication bias (Egger *P* < .001) (eAppendix 6 in Supplement 1), and the league table of head-to-head comparisons also showed that low-complexity (OR, 0.50; 95% CI, 0.32 to 0.77), medium-complexity (OR, 0.52; 95% CI, 0.33 to 0.81), and high-complexity (OR, 0.61; 95% CI, 0.40 to 0.92) interventions were significantly associated with reducing 30-day readmissions compared with minimal interventions.

Meta-regressions (eAppendix 7 in Supplement 1) showed that low-complexity interventions were associated with more efficacy for reducing 30-day readmissions when delivered by a health professional other than a nurse or social carer (β = −1.51; 95% CI, −2.47 to −0.56; *P* = .002) and in studies with high ROB (β = −0.88; 95% CI, −2.47 to −0.09; *P* = .04). Medium-complexity interventions were associated with more efficacy for reducing 30-day readmissions in studies based in Western Pacific (β = −0.84; 95% CI, −1.47 to −0.20; *P* = .01) compared with the Americas. Sensitivity analysis focused on discharge stages revealed that interventions applied at 1 discharge stage (OR, 0.68; 95% CI, 0.55 to 0.84; *P*-score = 0.99; *I*^2^ = 66%) and especially after discharge (OR, 0.56; 95% CI, 0.40 to 0.78; *P*-score = 0.90; *I*^2^ = 64%) were only associated with reducing 30-day readmissions (eAppendix 8 in Supplement 1).

#### 90-Day Hospital Readmissions

Using data from 34 studies^33,36,44,48,54,55,58,61,64,66,67,74,76,81,83,87,91,92,93,95,96,103,105,109,110,117,118,120,123,125,127,130,139,144^ (34 direct comparisons) involving 16 774 participants, medium-complexity (OR, 0.64; 95% CI, 0.45 to 0.92), and high-complexity (OR, 0.72; 95% CI, 0.57 to 0.91) interventions were associated decreased odds of readmission at 90 days compared with usual care (eAppendix 2 and eAppendix 9 in Supplement 1). Low-intensity interventions were not associated with reductions in readmissions (OR, 0.65; 95% CI, 0.41 to 1.02). The *P*-score ranked medium-complexity interventions (*P*-Score, 73%) as being associated with the most efficacy in reducing 90-day readmissions. Global heterogeneity of the network was seen to be moderate (*I^2^* = 68%; 95% CI, 50% to 78%), and since there was no indirect evidence, inconsistency assessment was not applicable (eAppendix 5 in Supplement 1). However, there was evidence for publication bias (Egger *P* < .001) (eAppendix 8 in Supplement 1).

Meta-regressions showed that low-complexity interventions were associated with less efficacy for reducing 90-day readmissions in non-OECD countries (β = 1.39; 95% CI, 0.35 to 2.44; *P* = .009) and when delivered by a professional who was not nurse, other HCP, or social carer (β = 2.30; 95% CI, 0.61 to 3.99; *P* = .008) or an MDT (β = 1.79; 95% CI, −0.51 to 3.06; *P* = .006). High-complexity interventions were less effective for reducing 90-day readmissions in studies involving mixed sexes (β = 0.89; 95% CI, 0.30 to 1.48; *P* = .003) compared with studies with more than 54% female participants.

Sensitivity analysis focused on discharge stage showed that interventions applied at 1 discharge stage were associated with the most efficacy for reducing 90-day readmissions (OR, 0.31; 95% CI, 0.16 to 0.59; *P*-score = 0.99, *I*^2^ = 65%) although interventions with any number of discharge stages were also associated with efficacy. Specifically, interventions at the postdischarge stage (OR, 0.31; 95% CI, 0.16 to 0.59; *P*-score = 0.95, *I*^2^ = 63%) were associated with the most efficacy in reducing 90-day readmissions.

#### 180-Day Hospital Readmissions

**Figure 4.**
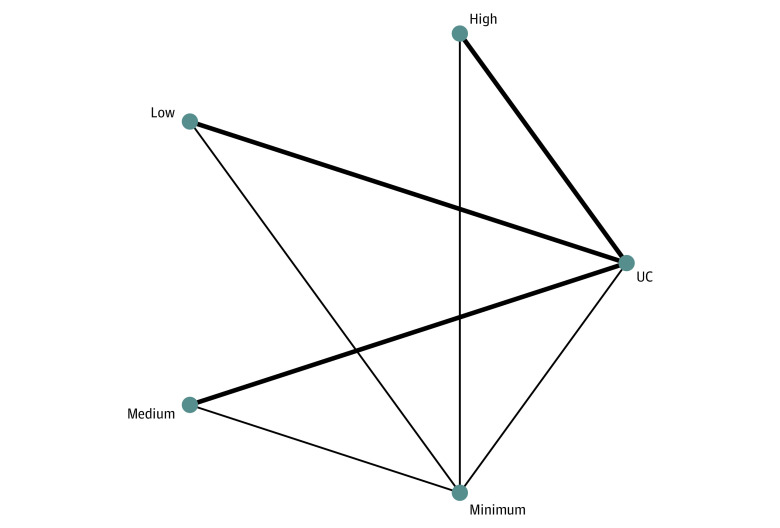
Network Meta-Analysis of Association of Discharge Intervention Intensities With Reducing 180-Day Readmissions Minimum indicates minimal intervention; UC, usual care. Line thickness indicates number of included studies.

Pooling data from 27 studies^9,36,40,44,48,55,58,62,75,77,82,85,95,96,97,99,106,107,124,130,134,136,138,140,141,143,145^ (34 direct comparisons) involving 13 039 participants, low-complexity (OR, 0.45; 95% CI, 0.30 to 0.66), medium-complexity (OR, 0.57; 95% CI, 0.35 to 0.91), and high-complexity (OR, 0.78; 95% CI, 0.62 to 0.98) interventions were associated decreased odds of readmission at 180 days compared with usual care (Figure 4). The *P*-score showed that low-complexity interventions (*P*-Score = 94%) were most effective in reducing the 180-day readmission odds. Global heterogeneity of the network was seen to be moderate (*I^2^* = 64%; 95% CI, 48% to 76%). No evidence of inconsistency in the model was found (eAppendix 5 in Supplement 1), and publication bias assessment revealed no concerns (Egger *P* = .06) (eAppendix 6 in Supplement 1). The only significant head-to-head finding from the league table of comparisons was that low-complexity interventions were associated with significantly better reductions in 180-day readmissions (OR, 0.57; 95% CI, 0.36 to 0.90) compared with high-complexity interventions.

#### ED Visits

**Figure 5.**
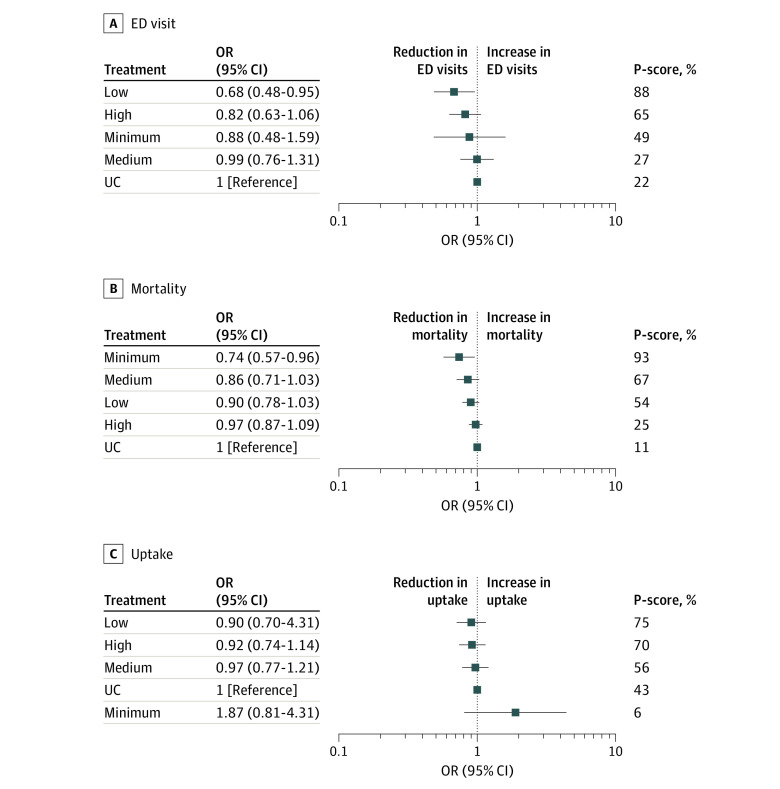
Association of Intervention Intensity With Emergency Department (ED) Visits, Mortality, and Intervention Uptake Minimum indicates minimal intervention; OR, odds ratio; UC, usual care.

Across 41 studies^7,9,29,30,31,34,35,37,39,47,48,49,50,52,56,57,58,60,61,67,68,73,78,80,87,88,94,102,105,106,113,115,119,121,125,130,132,137,145,146,148^ (45 direct comparisons) involving 28 034 participants, only low-complexity interventions (OR, 0.68; 95% CI, 0.48 to 0.96) were associated decreased odds of ED visits compared with usual care (Figure 5). The *P*-score showed that low-complexity interventions (*P*-Score, 87%) were associated with the most efficacy in reducing the odds of the ED visits. Global heterogeneity of the network was seen to be moderate (*I^2^* = 72%; 95% CI, 60% to 80%), and there was no evidence of inconsistency in the model (eAppendix 5 in Supplement 1). There was evidence of publication bias (Egger *P* = .03) (eAppendix 6 in Supplement 1).

#### Mortality

Across 42 studies^7,9,10,12,32,35,37,39,40,44,47,48,56,57,70,76,77,78,79,83,89,91,92,93,94,97,98,100,106,110,111,114,116,121,123,125,127,137,139,141,142,148^ (53 direct comparisons) involving 31 988 participants, none of the 3 intervention intensities were significantly associated with reducing mortality compared with usual care (Figure 5). Global heterogeneity of the network was seen to be very low (*I^2^* = 0%; 95% CI, 0% to 36%), and there was no evidence of inconsistency in the model (eAppendix 5 in Supplement 1). There was evidence of publication bias (Egger *P* = .001) (eAppendix 6 in Supplement 1). Meta-regressions did not reveal significant moderators for the association of intervention intensities with mortality.

#### Intervention Uptake

Pooling uptake data from 109 studies^7,8,9,10,11,12,13,14,29,30,31,32,33,34,35,36,37,38,39,41,43,44,47,49,50,51,52,53,55,56,57,58,59,60,61,62,63,64,65,66,67,68,69,70,72,73,74,75,76,77,78,79,80,81,83,84,85,86,87,88,91,92,93,94,95,96,97,98,99,101,102,103,104,106,107,111,112,113,115,116,117,118,119,120,121,122,123,124,125,126,128,129,131,132,133,134,135,136,137,138,139,140,141,143,144,145,146,147,148^ (123 direction comparisons) involving 82 623 participants, none of the intervention intensities were associated with increasing the odds of intervention uptake compared with usual care (Figure 5). Global heterogeneity of the network was seen to be moderate (*I^2^* = 63%; 95% CI, 51% to 67%), and there was no evidence of inconsistency in the model (eAppendix 5 in Supplement 1) or publication bias (Egger *P* = .41) (eAppendix 6 in Supplement 1). Meta-regressions showed that medium-complexity interventions were associated with lower uptake in studies involving mixed sexes (β = −0.78; 95% CI, −1.55 to −0.02; *P* = .046) and when delivered in Africa (β = −3.86; 95% CI, −5.41 to −2.30; *P* < .001).

### Pairwise Meta-Analyses

#### Adverse Events

Medium-complexity interventions were associated decreased odds of adverse events after discharge (5 studies^11,102,104,134,138^: OR, 0.42; 95% CI, 0.24 to 0.75) without heterogeneity (*I*^2^ = 0%; 95% CI, 0% to 75%). Low-complexity (3 studies^52,113,116^) and high-complexity (3 studies^52,84,106^) interventions were not significantly associated with reducing adverse events.

#### Patient Safety Incidents

Low-complexity interventions were associated with decreased odds of patient safety incidents (2 studies^14,52^: fixed-effects OR, 0.71; 95% CI, 0.53 to 0.94). High-complexity (4 studies^31,50,52,56^) and medium-complexity (5 studies^11,67,102,104,138^) interventions were not significantly associated with patient safety incidents.

#### Medication Adherence

High-complexity (5 studies^63,74,86,91,101^: SMD, 0.19; 95% CI, 0.03 to 0.36) and medium-complexity (7 studies^44,93,94,103,130,134,138^: SMD, 0.49; 95% CI, 0.30 to 0.67) interventions were associated with increases in medication adherence. Heterogeneity was low. Low-complexity interventions (3 studies^98,119,127^) were not significantly associated with medication adherence.

#### Length of Hospital Stay

High-complexity interventions were associated with reductions in the length of hospital stay (12 studies^56,65,71,72,74,87,90,99,101,109,110,112^: SMD, −0.20; 95% CI, −0.38 to −0.03). Heterogeneity was high (*I*^2^ = 75%; 95% CI, 56% to 86%). Low-complexity (6 studies^8,13,38,43,89,126^) and medium-complexity (5 studies^30,51,55,80,121^) interventions were not significantly associated with length of hospital stay.

#### Patient Satisfaction

High-complexity interventions were associated with increased patient satisfaction (7 studies^9,74,81,91,105,133,143^: SMD, 0.52; 95% CI, 0.22 to 0.82). Heterogeneity was moderate (*I*^2^ = 58%; 95% CI, 3% to 82%). Low-complexity (5 studies^7,13,32,43,115^) and medium-complexity (4 studies^62,67,80,130^) interventions were not significantly associated with patient satisfaction.

#### QOL

None of the intervention intensities were associated with significantly improved QOL. This included general (27 studies^9,32,38,44,56,65,66,83,84,85,91,97,105,107,110,112,114,123,125,126,132,133,140,141,145,148^), mental (8 studies^13,32,81,95,99,131,133,135^), or physical QOL (5 studies38,13,81,131,135) QOL measures among patients after discharge.

## Discussion

This systematic review and network meta-analysis found that low-complexity interventions, followed by medium-complexity interventions, especially those with a postdischarge component (eg, patient follow-up visit or phone call) were associated with the most efficacy in reducing health care utilization and mortality. These interventions were associated with between 18% and 55% reductions in hospital readmissions compared with usual care. High-complexity interventions were associated with reducing some health care utilization outcomes, but their associations were less pronounced. Moreover, we obtained preliminary evidence from pairwise meta-analysis that medium-complexity interventions might be best for reducing patient and medication harms (ie, adverse events^11,102,104,134,138^ and medication adherence^44,93,94,103,130,134,138^) whereas high-complexity interventions might be best for improving patient satisfaction.^9,74,81,91,105,133,143^ In general, the intervention complexity did not affect the intervention uptake; the only exception was that the uptake of medium-complexity interventions might be lower in low-resource settings, such as African countries, compared with high-resource countries. Moreover, an important but unintended finding of this review was that the range of outcomes reported by interventions was very narrow. Most trials reported hospital readmissions and, at best, some additional health care utilization outcomes (eg, ED visits,^7,9,29,30,31,34,35,37,39,47,48,49,50,52,56,57,58,60,61,67,68,73,78,80,87,88,94,102,105,106,113,115,119,121,125,130,132,137,145,146,148^ length of hospital stay^8,13,30,38,43,51,55,56,65,71,72,74,80,87,89,90,99,101,109,110,112,121,126^), and adverse outcomes (mortality^7,9,10,12,32,35,37,39,40,44,47,48,56,57,70,76,77,78,79,83,89,91,92,93,94,97,98,100,106,110,111,114,116,121,123,125,127,137,139,141,142,148^). Few trials have measured patient-reported outcomes (eg, QOL^9,32,38,44,56,65,66,83,84,85,91,97,105,107,110,112,114,123,125,126,132,133,140,141,145,148^ and patient satisfaction with the transitional care^7,9,13,32,43,62,67,74,80,81,91,105,115,130,133,143^) and broader risks for patient harm and safety^11,14,31,50,52,56,67,102,104,138^; none of the studies reported staff outcomes, despite the fact that transitional care interventions were mostly service delivery interventions relying on staff engagement for their success.

The evidence from previous reviews about the effectiveness of transitional care interventions from hospitals to the community is inconclusive. Direct comparisons with our findings are not possible because to our knowledge, this is the first network meta-analysis that examined the comparative effectiveness of intervention complexities. For example, some reviews have found little or no evidence that discharge planning interventions reduce readmissions,^20,26,27^ whereas other reviews concluded that intensive interventions promoting integrated systems between inpatient and community care and multidisciplinary working might be most effective.^149^ However, most of these systematic reviews have focused on hospital subsettings, were underpowered to detect significant reductions in readmissions, or their conclusions reflected the mixed findings of RCTs and observational studies. Our findings are partly consistent with the findings of a previous narrative systematic review^15^ that examined interventions to improve mental health care transitions and found that less complex interventions targeting 1 specific outcome, such as homelessness, were more likely to be successful compared with more complex interventions that aimed to reduce readmissions.

Our findings convey 3 key messages for clinicians and policymakers. First, low- and medium-complexity interventions may be the most effective options to reduce health care utilization and prevent ED visits for patients transitioning from hospital to the community. Second, the targets and benefits of high-complexity interventions must be reviewed. The achieved reductions in readmission rates may not show good value for the cost of high-complexity interventions,^23,150^ but improvements in patient and staff experience of discharge could better justify their costs and need for scalable implementation. Third, a core outcome set needs to be developed and used as standard practice by future trials of transitional care interventions. This core outcome set should complement health care utilization outcomes with patient-reported outcomes^151,152^ and staff-reported outcomes, as staff experiences are important for the success (ie, delivery as planned) and sustainability of service delivery interventions. Key prerequisites to inform actionable clinical practice and guidelines are better understanding of how patient factors and intervention mechanisms impact the effectiveness of transitional care interventions for patients transitioning from hospitals to the community, more comprehensive data on cost-effectiveness, and establishing core outcome sets to capture the full range of benefits and impacts of such interventions.

### Strengths and Limitations

This systematic review has numerous methodological strengths but has also important limitations. First, our classification approach is not flawless; for example, some of the components might be more important than others in improving all or some of the health care utilization or patient outcomes. Our classification system of the interventions is an integrated version of similar classification systems that previous reviews have used.^23,24,25,26,27,28^ We also included expert and patient and public involvement input when deciding on the intervention components of the classification system. However, we recommend that future trials adopt a more standardized approach to reporting the intervention components they have used. This practice will facilitate comparisons between different transitional care interventions and support similar meta-analyses in the future. Second, only a small proportion of the included studies had secondary outcomes including patient-reported outcomes, which precluded the use of network meta-analyses; nevertheless, these outcomes were quantified using pairwise meta-analyses. Moreover, although we did a series of network meta-regressions to identify factors associated with moderating the intervention outcomes, we were not able to examine whether clinical or social characteristics of patients (eg, frailty or multimorbidity, having carers) were confounders in our analyses due to low reporting quality of individual patient–level data.^153,154^ We used patient complexity as a moderator by comparing studies that explicitly described the patient group as high risk or high complexity or described multimorbidity, polypharmacy, vulnerability, or terminal illness across the whole patient population of the study. However, we recommend individual-patient data meta-analysis to reliably examine whether patient level factors, such as patient complexity or index disease, moderate the effectiveness of different interventions needed. Furthermore, realist reviews could shed further light into the mechanisms of action and implementation of transitional care interventions.^155^

## Conclusions

The findings of this systematic review and meta-analysis mostly supported the use of low- and medium-complexity transitional care interventions for reducing health care utilization for patients transitioning from hospitals to the community. We strongly recommend the development of a core outcome set that will include patient-reported and staff-reported outcomes to better capture the full range of benefits and impacts of transitional care interventions, especially high-complexity interventions.

## Notes

Supplement 1. **eAppendix 1.** Search Criteria **eAppendix 2.** League Table of Head-to-Head Comparisons **eAppendix 3.** Characteristics of Included Studies **eAppendix 4.** Risk of Bias Assessment **eAppendix 5.** Inconsistency Analysis **eAppendix 6.** Publication Bias and Funnel Plots **eAppendix 7.** Meta-Regressions **eAppendix 8.** Sensitivity Analysis **eAppendix 9.** 90-Day Readmissions and Secondary Outcomes

Supplement 2.
Data Sharing Statement
