# Supplementary material for: Transitional Care Interventions From Hospital to Community to Reduce Health Care Use and Improve Patient Outcomes: A Systematic Review and Network Meta-Analysis
Source: JAMA Netw Open. 2023 Nov 30;6(11):e2344825. doi: 10.1001/jamanetworkopen.2023.44825 (PMC10690480; doi:10.1001/jamanetworkopen.2023.44825)
Supplement: Supplement 1. — eAppendix 1. Search Criteria eAppendix 2. League Table of Head-to-Head Comparisons eAppendix 3. Characteristics of Included Studies eAppendix 4. Risk of Bias Assessment eAppendix 5. Inconsistency Analysis eAppendix 6. Publication Bias and Funnel Plots eAppendix 7. Meta-Regressions eAppendix 8. Sensitivity Analysis eAppendix 9. 90-Day Readmissions and Secondary Outcomes [file jamanetwopen-e2344825-s001.pdf]

Supplemental Online Content

Tyler N, Hodkinson A, Planner C, et al. Transitional care interventions from hospital to community to reduce health care use and improve patient outcomes. *JAMA Netw Open*. 2023;6(11):e2344825. doi:10.1001/jamanetworkopen.2023.44825

eAppendix 1. Search Criteria

eAppendix 2. League Table of Head-to-Head Comparisons

eAppendix 3. Characteristics of Included Studies

eAppendix 4. Risk of Bias Assessment

eAppendix 5. Inconsistency Analysis

eAppendix 6. Publication Bias and Funnel Plots

eAppendix 7. Meta-Regressions

eAppendix 8. Sensitivity Analysis

eAppendix 9. Readmissions and Secondary Outcomes

This supplemental material has been provided by the authors to give readers additional information about their work.

© 2023 Tyler N et al. *JAMA Network Open.*

Appendix 1: Search Criteria

Embase <1976 to 2022 Week 24>

1	(discharge and (plan* or service? or program* or intervention?)).ti.	3491
2	*Patient Discharge/	12544
3	(patient* adj2 discharge*).ti,ab.	79339
4	(hospital adj2 discharge*).ti,ab.	66005
5	(discharge adj2 plan*).ti,ab.	6903
6	(discharge adj service?).ti,ab.	325
7	(discharge adj program*).ti,ab.	363
8	(discharge adj procedure*).ti,ab.	211
9	2 or 3 or 4 or 5 or 6 or 7 or 8	146297
10	Length of Stay/	229440
11	Patient Readmission/	70908
12	(readmission or readmitted or re-admission or re-admitted).ti,ab.	64142
13	(rehospitali?ation* or re-hospitali?ation* or rehospitali?ed or re-hospitali?ed).ti,ab.	16183
14	length of stay.ti,ab.	128404
15	length of hospital stay.ti,ab.	43130
16	((hospital or hospitali?ed or bed) adj2 days).ti,ab.	23923
17	Continuity of Patient Care/	291167
18	10 or 11 or 12 or 13 or 14 or 15 or 16 or 17	632980
19	9 and 18	37190
20	1 or 19	39538
21	(random* or factorial* or crossover* or cross over* or cross-over* or placebo* or (doubl* adj blind*) or (singl* adj blind*) or assign*or allocat* or volunteer*).ti,ab.	2173756
22	crossover-procedure/ or double-blind procedure/ or randomized controlled trial/ or single-blind procedure/	771038
23	21 or 22	2281522
24	nonhuman/	6826093
25	23 not 24	2031840
26	20 and 25	4105

Ovid MEDLINE(R) <1946 to March Week 24 2021>

1	(discharge and (plan* or service? or program* or intervention?)).ti.	2353
2	*Patient Discharge/	15771
3	(patient* adj2 discharge*).ti,ab.	34481
4	(hospital adj2 discharge*).ti,ab.	37347
5	(discharge adj2 plan*).ti,ab.	4233
6	(discharge adj service?).ti,ab.	151
7	(discharge adj program*).ti,ab.	207
8	(discharge adj procedure*).ti,ab.	133

© 2023 Tyler N et al. *JAMA Network Open.*

9	2 or 3 or 4 or 5 or 6 or 7 or 8	78357
10	Length of Stay/	98678
11	Patient Readmission/	21189
12	(readmission or readmitted or re-admission or re-admitted).ti,ab.	28774
13	(rehospitali?ation* or re-hospitali?ation* or rehospitali?ed or re-hospitali?ed).ti,ab.	7848
14	length of stay.ti,ab.	58904
15	length of hospital stay.ti,ab.	22985
16	((hospital or hospitali?ed or bed) adj2 days).ti,ab.	12237
17	Continuity of Patient Care/	20287
18	10 or 11 or 12 or 13 or 14 or 15 or 16 or 17	193616
19	9 and 18	19573
20	1 or 19	21146
21	randomized controlled trial.pt.	561377
22	controlled clinical trial.pt.	94722
23	randomized.ab.	486697
24	placebo.ab.	207481
25	drug therapy.fs.	2458666
26	randomly.ab.	322856
27	trial.ab.	517730
28	groups.ab.	1999563
29	21 or 22 or 23 or 24 or 25 or 26 or 27 or 28	4860317
30	exp animals/ not humans.sh.	4974687
31	29 not 30	4173167
32	20 and 31	6266

APA PsycInfo <1806 to March Week 24 2021>

1	(discharge and (plan* or service? or program* or intervention?)).ti.	470
2	Discharge Planning/	506
3	*Hospital Discharge/	2021
4	(patient* adj2 discharge*).ti,ab.	3795
5	(hospital adj2 discharge*).ti,ab.	4351
6	(discharge adj2 plan*).ti,ab.	1320
7	(discharge adj service?).ti,ab.	55
8	(discharge adj program*).ti,ab.	37
9	(discharge adj procedure*).ti,ab.	38
10	2 or 3 or 4 or 5 or 6 or 7 or 8 or 9	9618
11	Length of Stay/	4396
12	Psychiatric Hospital Readmission/	1043
13	(readmission or readmitted or re-admission or re-admitted).ti,ab.	3273
14	(rehospitali?ation* or re-hospitali?ation* or rehospitali?ed or re-hospitali?ed).ti,ab.	2095
15	length of stay.ti,ab.	5579

© 2023 Tyler N et al. *JAMA Network Open.*

16	length of hospital stay.ti,ab.	982
17	((hospital or hospitali?ed or bed) adj2 days).ti,ab.	1361
18	(placebo* or random*).tw. or exp treatment/	1276732
19	11 or 12 or 13 or 14 or 15 or 16 or 17	15170
20	10 and 19	1719
21	1 or 20	2106
22	18 and 21	1912

EBM Reviews - Cochrane Central Register of Controlled Trials <September 2021>
EBM Reviews - Cochrane Database of Systematic Reviews <2005 to September 30, 2021>

1	(discharge and (plan* or service? or program* or intervention?)).ti.	601
2	Patient Discharge/	1723
3	(patient* adj2 discharge*).ti,ab.	8554
4	(hospital adj2 discharge*).ti,ab.	11838
5	(discharge adj2 plan*).ti,ab.	654
6	(discharge adj service?).ti,ab.	62
7	(discharge adj program*).ti,ab.	98
8	(discharge adj procedure*).ti,ab.	41
9	2 or 3 or 4 or 5 or 6 or 7 or 8	18941
10	Length of Stay/	7440
11	Patient Readmission/	1143
12	(readmission or readmitted or re-admission or re-admitted).ti,ab.	5876
13	(rehospitali?ation* or re-hospitali?ation* or rehospitali?ed or re-hospitali?ed).ti,ab.	2856
14	length of stay.ti,ab.	12222
15	length of hospital stay.ti,ab.	7767
16	((hospital or hospitali?ed or bed) adj2 days).ti,ab.	5788
17	Continuity of Patient Care/	642
18	10 or 11 or 12 or 13 or 14 or 15 or 16 or 17	34930
19	9 and 18	5431
20	1 or 19	5850

Appendix 2: League table of head-to-head comparisons

Results in the bottom left correspond to the *network meta-analysis* results, and results in the top right correspond to the *pairwise meta-analysis* findings. Results in **bold** as statistically significant

30-day readmission (odds ratio)

**Low**	0.74 (0.35 - 1.54)	0.83 (0.39 - 1.73)	**0.77 (0.65 - 0.91)**	0.81 (0.42 - 1.56)
0.96 (0.76 - 1.22)	**Medium**	.	**0.80 (0.67 - 0.96)**	.
0.81 (0.64 - 1.04)	0.85 (0.66 - 1.09)	**High**	0.98 (0.81 - 1.17)	0.85 (0.52 - 1.39)

© 2023 Tyler N et al. *JAMA Network Open.*

**0.78 (0.66 - 0.92)**	**0.81 (0.68 - 0.97)**	0.96 (0.80 - 1.15)	**UC**	**0.39 (0.23 - 0.64)**
**0.50 (0.32 - 0.77)**	**0.52 (0.33 - 0.81)**	**0.61 (0.40 - 0.92)**	**0.64 (0.42 - 0.96)**	**Min**

90-day readmission (odds ratio)

**Medium**	.	.	**0.64 (0.45 - 0.92)**
0.99 (0.56 - 1.77)	**Low**	.	0.65 (0.41 - 1.02)
0.90 (0.58 - 1.38)	0.90 (0.54 - 1.50)	**High**	**0.72 (0.57 - 0.91)**
**0.64 (0.45 - 0.92)**	0.65 (0.41 - 1.02)	**0.72 (0.57 - 0.91)**	**UC**

180-day readmission (odds ratio)

**Low**	.	.	0.87 (0.42 - 1.79)	**0.42 (0.28 - 0.63)**
0.79 (0.42 - 1.46)	**Medium**	.	.	**0.57 (0.35 - 0.91)**
**0.57 (0.37 - 0.90)**	0.73 (0.43 - 1.23)	**High**	0.73 (0.38 - 1.44)	0.79 (0.63 - 1.00)
**0.57 (0.34 - 0.96)**	0.72 (0.38 - 1.38)	0.99 (0.62 - 1.58)	**Min**	0.81 (0.50 - 1.32)
**0.45 (0.30 - 0.66)**	**0.57 (0.35 - 0.91)**	**0.78 (0.62 - 0.98)**	0.78 (0.51 - 1.22)	**UC**

ED visit (odds ratio)

**Low**	.	.	.	**0.68 (0.48 - 0.96)**
0.83 (0.54 - 1.29)	**High**	0.93 (0.46 - 1.87)	.	0.81 (0.62 - 1.06)
0.77 (0.38 - 1.56)	0.93 (0.50 - 1.71)	**Min**	.	0.88 (0.44 - 1.74)
0.69 (0.44 - 1.09)	0.83 (0.56 - 1.24)	0.89 (0.45 - 1.77)	**Medium**	0.98 (0.73 - 1.32)
**0.68 (0.48 - 0.96)**	0.81 (0.62 - 1.06)	0.88 (0.48 - 1.62)	0.98 (0.73 - 1.32)	**UC**

Uptake (odds ratio)

Low	0.63 (0.17 - 2.32)	1.13 (0.58 - 2.18)	0.86 (0.67 - 1.11)	0.86 (0.23 - 3.24)
0.97 (0.71 - 1.34)	**High**	.	0.92 (0.74 - 1.14)	**0.23 (0.06 - 0.86)**
0.93 (0.67 - 1.27)	0.95 (0.70 - 1.29)	**Medium**	0.98 (0.78 - 1.22)	.
0.90 (0.70 - 1.15)	0.92 (0.74 - 1.14)	0.97 (0.77 - 1.21)	UC	0.68 (0.26 - 1.79)
0.48 (0.20 - 1.13)	0.49 (0.21 - 1.15)	0.52 (0.22 - 1.23)	0.54 (0.23 - 1.24)	**Min**

Mortality (odds ratio)

**Min**	.	0.76 (0.54 - 1.07)	0.73 (0.48 - 1.11)	0.82 (0.58 - 1.15)
0.87 (0.61 - 1.25)	**Medium**	1.19 (0.52 - 2.73)	.	0.84 (0.68 - 1.02)
0.82 (0.60 - 1.12)	0.94 (0.74 - 1.20)	**Low**	1.00 (0.67 - 1.49)	0.90 (0.78 - 1.04)
0.76 (0.55 - 1.03)	0.87 (0.69 - 1.09)	0.92 (0.78 - 1.09)	**High**	0.98 (0.88 - 1.10)
0.74 (0.55 - 1.00)	0.84 (0.69 - 1.03)	0.90 (0.78 - 1.03)	0.97 (0.87 - 1.09)	**UC**

© 2023 Tyler N et al. *JAMA Network Open.*

Appendix 3: Characteristics of Included Studies

ID	Age (mean, SD)	Gender (% male)	Ethnicity	Condition (or setting if NR)	Control	Population (Medical/Mental Health)	Discharge stage	Complexity	Delivered by
Adamuz 2015	I=65, C=72 Overall - 68.5	I=61.9%, C=57.8%, O=60.0%	NR	Pneumonia	TAU	Medical	Pre	Low	Nurse
Arendts 2018	78.0 (+/-8.0)	62.00%	NR	Discharge from ED, Chest pain, SOB, Constipation, Fall, Urinary Frequency/Dysuria	TAU	Medical	Post/Bridging	Medium	Nurse
Auger 2018	2.0 (+/- 5.7)	51.90%	White=61.4% Black=29.0% Other=9.7%	Respiratory Diseases, Neurological Diseases, Gastrointestinal Diseases	TAU	Medical	Pre/Post/ Bridging	Medium	Nurse
Balaban 2008	I= 58, C= 54, historic C=61	I= 42.6% (C 38.8%, historic 40%)	NR	Chronic Conditions (diabetes, CHF, CAD, COPD, Depression)	historical and TAU	Medical	Pre/Post/ Bridging	High	Nurse
Barfar 2017	I=34.5 (12.4), C=36.1 (12.1)	I=56.3%, C=58.8%, O=57.5%	NR	Bipolar I disorder Schizophrenia-schizoaffective disorder	TAU	Mental health	Post/Bridging	High	GP + Social worker
Barker 2020	69 (11)	49%	NR	COPD	TAU	Medical	Pre/Post	Low	Technology (no staff)

© 2023 Tyler N et al. *JAMA Network Open.*

Bawazeer 2021	I=52.92 (15.9), C=53.83 (15.41)	I=41% C=38%	NR	Patients discharged with Warfarin or Insulin	TAU	Medical	Pre/Post	Medium	Pharmacist (students)
Bell 2016	60.0 (+/- 14.0)	59%	White=77%, Black=18.0%, Other=5%	Acute coronary syndrome, Congestive Heart Failure	TAU	Medical	Pre/Post/ Bridging	Medium	Pharmacist
Biese 2018	75 (+/-7.2)	39.90%	American Indian/ Alaskan=0.2%, Asian=0.4%, Black=19%, White=76.8%, Other=3.6%	Acute Care	TAU	Medical	Post/Brid ging	Medium	Nurse
Bloodworth 2019	I=52.63 (13.6) C= 57.54 (15.1)	I=57%, C=52%, O=53.88%	African American I=75%, C=64%, O=68.13%	Acute myocardial infarction Chronic obstructive pulmonary disease<br><br>Chronic heart failure Pneumonia	TAU	Medical	Pre/Post/ Bridging	High	Pharmacist
Bloom 2019	I= 56 (44–67), C= 56 (48–66)	I= 46%, C= 55%, O= 50.90%	White: I= 75%, C= 74%, O= 74.13%	Respiratory failure, Sepsis/septic shock, Altered mental status, Liver failure	TAU	Medical	Pre/Post/ Bridging	High	MDT
Blum 2014	72.5 (+/-9)	71%	African American=45% , White=55%	Chronic Heart Failure,	TAU	Medical	Post	Low	NR

© 2023 Tyler N et al. *JAMA Network Open.*

				Ischemia, T2DM					
Bonetti 2018	65 (+/- 11.5)	34.50%	NR	AMI, Unstable Angina, CHF, AF	TAU	Medical	Pre/Post	Medium	Pharmacist
Bonnet Zamponi 2013	86.1 (+/-6.2)	34%	NR	NR Polypharmacy Study	TAU	Medical	Pre/Bridging	High	Geriatrician
Bonsack 2016	I= 40 (11.9) C= l 41.3 (10.6)	I= 33.3%, C 48.1%	I= 84.3%, C=92.2% White	Mental Health (affective, neurotic, PD, psychotic disorder, substance use)	TAU	Mental health	Pre/Post/ Bridging	High	Nurse/Social worker
Bouchard 2021	69 (54-77)	61.6%	NR	At least one chronic disease (ie, diabetes mellitus, hypertension, pulmonary or cardiovascular disease)	TAU	Medical	Pre	Medium	Pharmacist
Bruhwiler 2019	71.5 (+/- 20)	45.50%	NR	Discharges from Stroke, Respiratory, ID, Nephrology, Cardiology and Geriatric Wards	TAU	Medical	Pre	Low	Pharmacist
Chen 2018	61 (+/- 15)	56.40%	NR	Congestive Heart Failure	TAU	Medical	Pre/Post	Medium	Nurse
Coskun 2022	I=60-69=25, >70=8 C 60-69=18, >70=14	I=71.9% C=84.4%	NR	Cardiac	TAU	Medical	Pre/Post/ Bridging	High	Nurse + MDT

© 2023 Tyler N et al. *JAMA Network Open.*

Danielsen 2020	I=67.3 (9.8), C=65.8 (11,1)	I=71.6%, C=70.9%	NR	Heart Problems	TAU	Medical	Post	Low	Nurse + project co-ordinator
Dawes 2007	I=46.8 (+/-11.5) C=46.4 (+/-12.6) O=46.6 (+/-12.0)	0%	NR	Major Surgery for benign Gynae Disease	TAU	Medical	Pre	Low	Nurse
Dawson 2021	I=67 (14), C=66 (15)	52.30%	Black 12%<br>Asian 1.2%<br>White 83%<br>Other 1.5%<br>Unknown 2%	All admissions	TAU	Medical	Post	Low	Nurse
Devore 2021	I = 62.3 (13.8) C=62.9 (13.4)	I= 67.2% C=66.2%	I= White 55/9%<br>Black 35.9%<br>Asian 2.8%<br>Other 6.5%<br><br>C= White 56.3%<br>Black= 40.5%<br>Asian= 0.6%<br>Other= 3.7%	Cardiac	TAU	Medical	Pre/Post/ Bridging	Low	MDT
Dhalla 2014	I=71.2 (+/-16.1), C=71.3 (+/-16), O=71.2 (+/-16)	I=51%, C=52%, O= 51.3%	NR	High risk of readmission as defined by LACE criteria	TAU	Medical	Post/Bridging	High	Interprofessional team of co-ordinators
Edey 2018	I=70.6, C=67.8, O=69.0	I=50% C=53% O=51.6%	NR	Defined via speciality, Cardiovascular, Endocrine, GI, Haematology, ID, Malignancy, MSK, Derma,	TAU	Medical	Pre/Bridging	Low	Pharmacist

© 2023 Tyler N et al. *JAMA Network Open.*

				Neuro, Psych, Renal, Respiratory, Rheum, Urology					
Englander 2014	Mean NR	I=59.3% C=59.2% O=60.2%	Non-white=28% White=72%	Readmission after discharge from Cardiology or General Medicine Ward	TAU	Medical	Pre/Post/ Bridging	High	Nurse + pharmacist
Evans 1993	I= 184/417 > 70. C= 198/418 > 70.	I = 96%, C=94%	NR	Medical, neurological, or surgical services	TAU	Medical	Pre/Bridging	Medium	NR
Farris 2014	61(+-12)	NR	91% White	Cardiovascular related, Asthma, COPD.	TAU	Medical	Pre/Post/ Bridging	High	Pharmacist
Finlayson 2018	O= 77.6 (6.64)	O= 27%	NR	Primary admission: Respiratory disease Cardiac disease Renal Falls Other Following Comorbidities: Cardiovascular disease Orthopaedic Respiratory disease Gastrointestinal Endocrine	Active Control	Medical	Post	Low	Nurse + exercise specialist

© 2023 Tyler N et al. *JAMA Network Open.*

				Renal<br>Other					
Finn 2020	Phase 1: I= 80.58 ± 9.1, C= 80.76 ± 9.23; 9; Phase 2: I=82.05 ± 6.8 2 C= 84.17 ± 7.4;	Phase 1: 53.3%; Phase 2: 51.3%	NR	Not reported. Hospitalisation.	TAU	Medical	Pre	Low	Pharmacist
Gardner 2020	I= 83.3 (5.3); C=81.3 (5.4)	I= 42.1%; C=42.7%	NR	NR	TAU	Medical	Pre/Post/ Bridging	High	SNF/ nursing home staff
Gharadi-Vasfi 2015	I= 34.1 (10), C= 36.5 (10.1)	I= 63% C=72%	NR	Severe Mental Health	TAU	Mental health	Post/Brid ging	Medium	NR
Gilbert 2021	I= 86.8 (5.4); C=87 (5.5)	I= 36.6%; C= 36.3%	NR	All admissions	TAU	Medical	Pre/Post/ Bridging	High	Nurse
Gillard 2022	39.7 (13.7)	I=48% C=45%	I= Asian or Asian British 13%; Black, African, Caribbean or Black British 16%; Mixed/multiple ethnic groups 11%; Other ethnic group 3%; White 58%.<br>C= Asian or Asian British 11%%; Black, African, Caribbean or Black British	Mental Health inpatients	TAU	Mental Health	Pre/Bridgi ng	Medium	Peer Worker

© 2023 Tyler N et al. *JAMA Network Open.*

			16%; Mixed/multiple ethnic groups 6%; Other ethnic group 2%; White 65%						
Gillespie 2009	I= 86.4 (4.2)<br>C= 87.1 (4.1)	I=42.3%,<br>C=40.3%	NR	Heart failure<br>Diabetes mellitus<br>Pulmonary disease<br>Arrhythmia<br>Malignant disease, past and present coronary artery disease<br>Cerebral vascular lesion, past<br>Myocardial infarction<br>Hypertension<br>Dementia	TAU	Medical	Pre/Post/ Bridging	Medium	Pharmacist
Goldman 2014	66.2 (9.0)	56.50%	Black/African American=24.5 %. Latino/Hispanic 19.6%, White= 19.0%, Other/declined =24.5%, Filipino=5.9%, Other Asian=1.9%	NR	TAU	Medical	Pre/Post/ Bridging	High	Nurse

© 2023 Tyler N et al. *JAMA Network Open.*

Graabaek 2018	C=75.0<br>I1=74.8<br>I2=75.5<br>O=75.4	I1=52%<br>I2=50%<br>C=51%<br>O=51%	NR	Neurology, Cardiology, General Medicine	Minimal	Medical	Pre	Low	Pharmacist
Gurwitz 2014	79 (+/- 7.2)	47.50%	NR	Diabetes, MI, Heart Failure, COPD, Cancer, Stroke, Renal Disease	TAU	Medical	Pre/Bridging	Medium	Physician + primary care provider
Habib 2021	64.6 (45.3-75.0)	61.2%	NR	Internal Medicine	TAU	Medical	Pre/Post	Medium	Digital
Hanrahan 2014	I= 44.1 (11.2) C= 45.8 (11.9)	I= 55% C 50%	I: African American=45%, White =30%, Asian=5%, More than one race=10%, Other 10%, Hispanic=5% C: African American= 45%, White= 30%, More than one race =20%, Hispanic/Latino=10%,	Mental Health (major depression, bipolar, schizophrenia, schizoaffective disorder, schizophrenia, psychosis, personality disorder, substance use)	TAU	Medical	Post/Bridging	High	Nurse
Harrison 2002	75.64 (9.87)	55%	NR	Heart Failure	TAU	Medical	Pre/Post/ Bridging	High	Nurse
Harvey 2014	I=83.8 (+/-7) C=86.7 (+/-7) O=85.3 (+/-7.1)	I=33%<br>C=41%<br>O=37%	NR	Pneumonia, UTI. Heart failure, Anaemia, Volume depletion, Cellulitis, Chest	TAU	Medical	Post/Bridging	Medium	Geriatrician + nurse

© 2023 Tyler N et al. *JAMA Network Open.*

				pain, Cerebrovascular Event, COPD					
Heaton 2019	O=61.7 (12.7)	52.80%	White= 79.5%, Black=18.3%, Other=2.3%	Congestive heart failure Chronic obstructive pulmonary disease Diabetes mellitus Myocardial infarction Pneumonia	TAU	Medical	Post/Bridging	High	Pharmacist
Hegelund 2019	I=73 (+/-10.4) C=72 (+/-8.1) O=72.5 (+/-9.2)	I=39% C=43% O=41.4%	NR	COPD	TAU	Medical	Pre/Post	Medium	Nurse
Hegelund 2020	I= 73 (67-81), C=72 (67-78)	I=39%, C=43%	NR	COPD	TAU	Medical	Pre/Post	High	Nurse
Hengartner 2016	I= 42.1 (11.4), C=41.0 (11.3)	I= 50.6%, C 49.4%	NR	Mental Health (Psychosis, mood disorder, other)	TAU	Mental health	Pre/Post/Bridging	High	Social worker + someone from network
Hosein Abadi	I= 62.3 (10.4); C= 62.3 (12.4)	I= 73.3%; C=66.7%	NR	Heart Failure	TAU	Medical	Pre/Bridging	High	Researcher
Hu 2020	I= 32.98 (7.73); C= 31.79 (9.21)	I= 70%; C= 59.2%	NR	Kidney transplant recipient	TAU	Medical	Pre/Post	Medium	Nurse (Dr assists with discharge plan development)
Indraratna 2022	I=61.3 (12.3) C= 61.7 (12.6)	I= 80% C=78%	NR	Cardiac	TAU	Medical	Bridging	Low	MDT

© 2023 Tyler N et al. *JAMA Network Open.*

Jack 2009	I=50.1 (15.1), C= 49.6 (15.3).	I= 52%, C= 47%.	White non-Hispanic I= 28%, C=27%, Black non-Hispanic I=51%, C=52%, Hispanic I=10%, C=10%, Other race or mixed race I=10%, C=10%.	All admissions	TAU	Medical	Pre/Post/ Bridging	High	Nurse + pharmacist
Jayaram 2022	I= 38 (13) C= 41 (17)	I=23% C=43%	NR	Asthma	TAU	Medical	Pre/Post/ Bridging	High	Nurse + MDT
Jennings 2015	I=64.88 (+/- 10.86) C=64.43 (+/- 10.47) O=64.7 (+/- 10.7)	I=43.4% C=46.8% O=44.8%	White=24.4% Black=75% Asian=0.6%	COPD, GORD, Depression, Anxiety	TAU	Medical	Pre/Post/ Bridging	Low	Nurse
Karaoui 2020	I= 74.69 (12.09); C= 73.15 (14.74), O= 73.9 (no SD reported)	I= 46%; C= 43%	NR	Indication for anticoagulation: Atrial fibrillation, venous thromboembolism, aortic valve replacement, mitral valve replacement. Baseline assessed Conditions: hypertension, HF, CKD, Cancer, history	TAU	Medical	Pre	Low	Pharmacist

© 2023 Tyler N et al. *JAMA Network Open.*

				of bleeding, history of gastrointestina l bleeding					
Kennedy 1987	I= 80.05. C =80.53	I =51%. C= 44%.	White I = 90%. White C= 98%, Black I=10%. Black C= 0%	ICU	TAU	Medical	Pre/Post/ Bridging	High	Nurse
Ko 2017	I=74.9±7.9, C=74.6±8.6, O= 74.8, 8.3	I=94.4%, C=96.7%, O=95.55%,	NR	COPD is Primary, following Comorbidities: Hypertension, Diabetes mellitus Hyperlipidaemi a Ischaemic heart disease Heart failure Old pulmonary TB	TAU	Medical	Post/Brid ging	High	Nurse + physio + respiratory specialist
Kowalkowski 2022	I= 64.7 ± 14.9 C= 62.7 ± 15.5	I=47% C=49.1%	I= White 68.5, Black 27.8, Other 3.7, C = White 61.1, Black 32.5, Other 6.4	Sepsis	TAU	Medical	Bridging/ Post	Medium	Nurse
Kripalani 2012	I=61(14), C= 59(14).	I=59.1%, C=58.2%.	White I= 75.4% Black I=18.2, Other T=6.4 White C=78.3, Black C= 16.6, Other=5.1%	Acute coronary syndromes or acute decompensate d heart failure.	TAU	Medical	Pre/Post	Medium	Pharmacist + study co-ordinator
Lainscak 2013	71 (9)	71%	NR	Primary admission for	TAU	Medical	Pre/Post/ Bridging	High	Discharge coordinator

© 2023 Tyler N et al. *JAMA Network Open.*

				COPD. Comorbidities reported: Ischemic heart disease Arterial hypertension Heart failure Atrial fibrillation Diabetes mellitus Malignant disease Asthma					
Laramee 2003	70.7 (11.8)	I= 58%. C=50%	NR	CHF. Comorbidities: Hypertension Diabetes mellitus COPD Peripheral vascular disease Smoker Hyperlipidaemia Obesity Prior myocardial infarction Myocardial infarction this admission Ischemic origin for heart failure	TAU	Medical	Pre/Post/ Bridging	High	Case manager

© 2023 Tyler N et al. *JAMA Network Open.*

Latour 2006	I=65.29 (+/-15.74), C=62.32 (+/-17.5), O=63.9 (+/-16.6)	I=50%, C=52.2%, O=51%	NR	Endocrine, Circulatory, Respiratory, GI, Infectious disease, Other	TAU	Medical	Post/Bridging	High	Nurse
Lavesen 2016	I=69.72 (+/-10.3) C=70.9 (+/-9.79) O=70.2 (+/-10.1)	I=38.7% C=39.4% O=39%	NR	COPD	TAU	Medical	Post/Bridging	Medium	Nurse
Lea 2020	I 78 (25.7-95.6), C 80.7 (23.1-96.4)	I=47%, C=45%	NR	Multimorbid patients	TAU	Medical	Pre/Bridging	Low	Pharmacist Medical
Legrain 2011	I= 85.8 (6.0). C= 86.4 (6.3)	I= 30.3%. C=37.4%.	NR	Dementia, Stroke, Hypertension, Diabetes Mellitus, Heart Failure, Coronary Artery Disease, Arrhythmia, Chronic Pulmonary insufficiency, Cancer.	TAU	Medical	Pre/Bridging	High	Geriatrician
Lembeck 2019	I=82.5 (7.6), C=82.2 (7.3)	I=44%, C=36%, O=39.85%	White=98%	NR Frailty	TAU	Medical	Post/Bridging	Low	Nurse
Levine 2018	I=65(28); C=60(29)	I=78%; C=27%; O=50%	I= White 44%, Latino 44%, Black 11%; C= White 45%, Latino 27%, Black 27%	Heart failure, chronic obstructive pulmonary disease, or asthma.	TAU	Medical	Post/Bridging	Medium	MDT

© 2023 Tyler N et al. *JAMA Network Open.*

Li 2014	I=57.4 (+/-12.8) C=55.2 (+/-11.9) O=56.3 (+/-12.4)	I=60.9% C=56.1% O=58.5%	NR	End Stage Renal Failure	TAU	Medical	Pre/Post/ Bridging	High	Nurse
Li 2018	I 1=60 ± 15, I 2= 62 ± 14, C= 61 ± 15	I1=57.5%, I2=54.5%, C=57.3%, O=56.45%	NR	HF is primary, Secondary Ischaemic heart disease non-ischaemic cardiomyopathy Valvular heart diseases Hypertension Diabetes mellitus COPD Chronic renal disease	TAU and Active	Medical	Pre/Post	Medium	Nurse
Lin 2014	I=62.1, C=63.4, O=62.8	C=63% I=56% O=60%	NR	Cardiology, Respiratory and Endocrine wards	TAU	Medical	Pre	Low	Nurse
Lindhart 2019	I1=74 (+/-7) I2=75 (+/-7) C=75 (+/-7) O=75 (+/-6.9)	I1=44.6% I2=53.2% C=39.4% O=45.8%	NR	Frailty as assessed by Handgrip strength and chair to stand test	Active Control	Medical	Pre/Post/ Bridging	Medium	Nurse
Liu 2020	49.69 (13.55)	O= 21.6%	NR	Rheumatoid arthritis	TAU	Medical	Pre/Post/ Bridging	High	Specialist nurse
Lockwood 2019	I=83.4 (+/-7.1) C=80.9 (+/-7.3) O=82.1 (+/-7.3)	I=24% C=32% O=28.6%	NR	Hip Fracture Surgery	TAU	Medical	Pre/Post/ Bridging	Medium	Occupational therapist

© 2023 Tyler N et al. *JAMA Network Open.*

Magny-Normilus 2021	I=65 (11.3), C=63.9 (11.6)	I=49% C=33%	I= Black=27.3%, Hispanic =38.6% White =33% Other= 1.1%, C= Black=31.5%, Hispanic=32.6 %, White=34.8%, Other=1.1%	Type 2 diabetes (and active cardiovascular disease)	TAU	Medical	Pre/Post/ Bridging	High	Nurse + Pharmacist + GP/Inpatient clinician
McWilliams 2019	I=58.3 (+/-17.7) C=59.4 (+/-17.4) O=58.9 (+/-17.6)	I=48.9% C=47.5% O=48.2%	American Indian or Alaska Native= 0.2% Asian=1.3% Black=39% White=50.3% Other=7.2%. Missing n=13	COPD, CHF, T2DM, ESRF	TAU	Medical	Pre/Post/ Bridging	High	MDT
Mehta 2020	66 (58-73 IQR)	21.90%	White=45.5%, Black=43.4%, Asian=2.1%, Hispanic =1.2%, Other=7.8%	Hip and Knee Arthroplasty	TAU	Medical	Post	Low	Surgeon + nurse + social worker
Meisinger 2013	I=75.2 (+/-6) C=75.6 (+/-6) O=75.4 (+/-6)	I=63.7% C=61.3% O=62%	NR	Congestive Heart Failure and Diabetes	TAU	Medical	Pre/Post/ Bridging	High	Nurse
Moher 1992	Reported as Young and Old	45.70%	NR	NR (all admissions except ICU, and expected imminent death)	TAU	Medical	Pre/Bridging	Low	Nurse

© 2023 Tyler N et al. *JAMA Network Open.*

Naylor 1994	75.5	Medical DRG- I=57%. C=41%. Surgical DRG: I=82%, C=61%.	White: Medical DRG- I: 61%. C: 69%. Surgical DRG: I: 97%, C: 98%	NR	TAU	Medical	Pre/Post/ Bridging	High	MDT
Nazareth 2001	Over 75	NR	NR	NR	TAU	Medical	Pre/Post/ Bridging	High	Pharmacist
Negarandeh 2019	O=54.9	I=60% C=60.6% O=60.3%	NR	CHF	TAU	Medical	Post	Medium	Nurse
Nguyen 2018	I=62 (+/-8.4), C=59.8 (+/-8.8), O=61.2 (+/-9.6)	I=77.2% C=67.8% O=72.3%	NR	Acute coronary syndrome, MI, Stroke, CHF, Renal Failure, Peptic Ulcer, Asthma, COPD	TAU	Medical	Pre/Post	Medium	Pharmacist
Noel 2020	I= 65.66 (13.24); C= 63.67 (14.78)	I=36%, C=37%	Non-Caucasian= 16%	NR (hospitalised patients with 2 or more chronic conditions)	TAU	Medical	Pre/Post	Medium	PCP provider or clinical trainee reporting to PCP provider
O'Cnell 2018	I=42.1 (+/-12) C=38 (+/-9.3) O=40.1 (+/-10.9)	I=44% C=57% O=50%	Black=26% White=59% Unknown=15.8 %	Mood Disorder, Psychotic Disorder (schizophrenia, schizoaffective disorder, psychotic disorder not otherwise specified, bipolar disorder (type	TAU	Mental health	Post	low	Peer mentor

© 2023 Tyler N et al. *JAMA Network Open.*

				I, type II, or not otherwise specified), or major depressive disorder with or without psychotic features)					
Odeh 2020	I= 67.3; C= 67.6	46%	NR	NR	TAU	Medical	Pre/Post/ Bridging	High	Pharmacists
Ong 2016	I=73 (IQ of 62-84), C= 74 (63-82)	53.80%	African American I=21.5% (18.5-24.5) C= 22.7% (19.6-25.8) O= 22.1%, Hispanic/Latino I=12.0% (9.6-14.3), C= 10.9% (8.6-13.1), O=11.45%, White I=54.7% (51.0-58.4), C= 54.3% (50.7-58.0), O=54.50%, Asian/Pacific Islander or other I=11.8% (9.4-14.2) C= 12.1% (9.7-14.5) O=11.89%	Heart Failure	TAU	Medical	Pre/Post/ Bridging	High	Nurse

© 2023 Tyler N et al. *JAMA Network Open.*

Oscalices 2019	62.6 (+/- 15.2)	59.20%	NR	CHF, HTN, T2DM, Dyslipidaemias	TAU	Medical	Pre/Post	Low	Researcher
Ougrin 2020	I=16.23 (1.54) C=16.34 (1.70)	I= 32.1% C=37.7%	I=White British 52.8%, Other 47.2%, C =White British 45.3, Other 54.7	Adolescent Mental Health	TAU	Mental health	Pre/Post/ Bridging	High	Consultant +admin+ nurses (community)+ clinical support workers
Pardessus 2002	83.2 (7.7)	21.60%	NR	Hospitalised for Falls	TAU	Medical	Pre/Bridgi ng	Low	Occupational therapist
Parsons 2020	I=81.1(7.8), C=80.5(8.3)	I=23.2%, C=27.3%, O=24.81%	European I=97.5%, C=97.5, O=97.5%. Māori (indigenous population) I= 2.0%, C=1.5%, O=1.75% Other I=0.5% C=1.0%. O=0.75%	Traumatic brain injury Spinal fracture Soft tissue Clavicle, shoulder, and humeral fracture Wrist and forearm fracture Pelvic fracture Hip fracture Femur and knee fracture Tibia, fibula, ankle, and foot fracture. Other fracture	TAU	Medical	Post/Brid ging	High	Health care assistants
Phatak 2016	I=55.4, C=55.8 (no SDs given)	I=37.95%, C=41.8%, O=39.92%	NR	Cardiovascular disease Pneumonia Respiratory Infectious	TAU	Medical	Pre/Post/ Bridging	Medium	Pharmacist

© 2023 Tyler N et al. *JAMA Network Open.*

				disease<br>Gastrointestinal<br>Endocrine<br>Genitourinary<br>Haematological<br>Injury<br>Neurological<br>Heart failure<br>Myocardial infarction<br>Mental/substance abuse					
Piette 2020	60.7 (12.9)	46.30%	White 80.2%	Admitted to acute hospital	TAU	Medical	Post/Bridging	Medium	Electronic voice recorder
Pourrat 2020	c/I sequence: 61.6(16.5), I/C sequence 63.7 (16.7)	C/I = 52.3%, I/C = 57.85%	NR	All general admissions	TAU	Medical	Pre/Post/ Bridging	Medium	Pharmacist
Qian 2019	I=66.54 ± 10.16, C= 64.8 ± 10.49	I=77%, C= 78% O=77.78%	NR	Stroke	TAU	Medical	Pre/Post/ Bridging	High	Nurse
Ravn Nielsen 21018	Basic I=72 (63-80). Extended I: 71 (63-79) C= 73 (65-80)	Basic I:49.7%, Extended I: 45.0%, C=44.2%	NR	Heart failure<br>Diabetes<br>Hypertension<br>Arrhythmia<br>Malignant diseases<br>Cerebral vascular lesion<br>Myocardial infarction<br>Pulmonary	TAU	Medical	Pre/Post/ Bridging	High	Pharmacist

© 2023 Tyler N et al. *JAMA Network Open.*

				diseases<br>Dementia					
Reynolds 2004	NR	NR	NR	Mental Health (bipolar disorder, schizophrenia, and depression,	TAU	Mental health	Post/Bridging	Medium	Nurse
Rich 1993	I= 80.0 (6.3). C=77.3 (6.1)	I= 39.7%. C= 42.9%	White I = 46.0%. White C= 57.1%,	Primary Chronic Heart Failure, Secondary: Hypertension Diabetes mellitus Prior myocardial infarction Prior Congestive heart failure	TAU	Medical	Pre/Post/Bridging	High	Nurse + cardiologist
Rich 1995	I=80.1 +-5.9, C=78.4 +-(6.1)	I= 32%. C= 41%	"Non-white race": C=59%. I=52%	Primary Chronic Heart Failure, Secondary: Hypertension Diabetes mellitus Prior myocardial infarction Prior Congestive heart failure	TAU	Medical	Pre/Post/Bridging	High	MDT

© 2023 Tyler N et al. *JAMA Network Open.*

Ritchie 2016	63.4(+/-12.5)	52.40%	White=53.1%, Black/Other=46.9%	CHF, COPD	TAU	Medical	Pre/Post	Medium	Nurse
Sahota 2017	84.1 (+/-6.3)	36.00%	NR	Admitted for 'Acute Medical Emergency'	TAU	Medical	Pre/Post/ Bridging	high	Nurse
Salameh 2018	I=62.3 (15.6), C=63.9, (13.4)	I=56.9%, C=54.1%	NR	Respiratory Gastroenterology Cardiology Neurology Nephrology/Urology Oncology/haematology Infectious Endocrinology Rheumatology	TAU	Medical	Pre	Low	Pharmacist
Sales 2014	I=72.5 (+/-14.8) C=72.6 (+/-13.4) O=72.6 (+/-64.1)	I=37.1% C=47.8% O=42.3%	NR	Heart Failure	TAU	Medical	Pre/Post	Medium	Trained volunteer
Salmany 2018	I=47.2 (16), C=49.2 (16)	I=45.7%, C=47.3%	NR	Cancer	TAU	Medical	Post/Bridging	Low	Pharmacist
Santana 2017	I=58.8 (17.9), C=59.1 (17.9) overall - 59, 17.9	I= 46.6%, C=53.4%, O= 53.6%	Caucasian I: 78.2% C: 79.5%, O: 78.84%, Non-Caucasian: I: 21.8%, C: 20.5%, O: 21.15%	Diabetes with organ damage Chronic pulmonary disease Liver disease renal disease Congestive heart failure Multiple diagnoses	TAU	Medical	Pre/Bridging	Low	Medical residents

© 2023 Tyler N et al. *JAMA Network Open.*

				Respiratory Infections<br>Renal/Kidney<br>Metabolic/Electrolyte disorders<br>Cardiac<br>Loss of Consciousness<br>GI/Hepatic<br>Poison/Toxic<br>Deep vein thrombosis/pulmonary embolism					
Shahrokhi 2017	I=34.11 ± 12.34<br>C=31.12 ± 10.83	73.52%	NR	Head Trauma	TAU	Medical	Post	Low	Nurse
Shaw 2000	47 (17)	37%	NR	NR	TAU	Medical	Pre/Post/Bridging	High	Pharmacist
Strano 2019	I=78.3 (12.5),<br>C=79.9 (7.9),<br>Total=79.0 ± 10.5	41.8	NR	HF, Chronic lung disease<br>Hypertension<br>Diabetes<br>Renal failure	TAU	Medical	Post	Low	Nurse
Sudas Na Ayutthaya 2017	57.7 (+/-10.9)	40%	NR	Atrial Fibrillation, DVT, Valvular Heart Disease, Mechanical Valves, PE	TAU	Medical	Post	Low	Pharmacist
Thygesen 2015	77.8 (+/-7.6)	52%	NR	Dementia, Acute Medical/Surgical Treatment,	TAU	Medical	Post/Bridging	Medium	GP + nurse

© 2023 Tyler N et al. *JAMA Network Open.*

				Active Psychiatric Treatment, Substance Abuse					
Tomita 2012	37.5 (9.5)	71%	62% African American	Mental Health (Schizophrenia, schizoaffective disorder, substance misuse)	TAU	Mental health	Post/Bridging	Medium	Social worker
Utens 2012	I=68.3 (+/-10.3), C=67.8 (+/-11.3), O=68.1 (+/-10.8)	C=55.1% I=68.6% O=61.9%	NR	COPD	TAU	Medical	Pre/Post/Bridging	Medium	Nurse
van der Hejiden 2019	74.7 (+/-8.8)	47.75%	NR	NR	TAU	Medical	Pre/Post/Bridging	High	Pharmacist
Van Spell 2019	I=77.77 (12.42), C=77.59 (11.89)	I=50.7%, C=48.6%, O=49.56%	NR	Heart Failure	TAU	Medical	Pre/Post/Bridging	High	Nurse
Vesterby 2017	I=63 (range 43-80), C=64 (range 45-84)	I=52.78%, C=52.78%, O=52.78%	NR	NOF fracture, other	TAU	Medical	Post	Low	Physio
Vinluan 2015	I=74 ± 5.9, C=71 ± 6.9	I=14%, C=22%, O=18.75%	NR	Heart Failure	TAU	Medical	Pre/Post	Low	Pharmacist
Webster 2011	I=52.2 (+/-18.2) C=45.9 (+/-17.7) O=49.1 (+/-18.2)	I=63.7% C=63.7% O=63.7%	NR	Discharge after surgery, Surgeries Listed. ENT, MaxFac, Ortho (elective +	TAU	Medical	Pre	Low	Nurse

© 2023 Tyler N et al. *JAMA Network Open.*

				trauma), General, Urology, Plastics, Burns					
Weiss 2019	59.59 (17.54)	48.80%	American Indian or Alaska Native =0.8%, Asian 3.3%, Black, or African American = 14.5%, Hawaiian or Pacific Islander =0.3%, White =65.0%, Unknown = 16.1%	Medical/Surgical Unit	Active Control	Medical	Pre	Low	Nurse
Wong 2004	I=61.3 (+/-11) C=63.7 (+/-11.1) O=62.1 (+/-11)	I=61.5% C=51% O=56.4%	NR	Type 2 Diabetes	TAU	Medical	Pre/Post	Medium	Nurse
Wong 2014	Overall=76.5	NR	NR	NR	Active Control	Medical	Pre/Post/ Bridging	Medium	Nurse
Wong 2015	I=67.5(11.6), C= 71.5(11.6), O=69.5 (11.8)	I=37%, C=37% O=37%	NR	Stroke, Haemorrhagic and Ischaemic split	TAU	Medical	Pre/Post/ Bridging	High	Nurse
Wong 2016	78.4 (+/-13.4)	52.50%	NR	Chronic Heart Failure	TAU	Medical	Pre/Post/ Bridging	High	MDT
Wu 2019	I=70.2(6.2), C=68.6(5.8) O= 68.6	I=68.6%, C=71.4%	NR	Acute MI	TAU	Medical	Pre/Post	Medium	NR

© 2023 Tyler N et al. *JAMA Network Open.*

	(SD 5.8) 69.4 (6.1)								
Xie 2018	37.1 (+/- 14.1)	14%	NR	Systemic Lupus Erythematous	TAU	Medical	Post	Medium	Nurse
Xu 2019	I=63.24 ± 10.19, C=64.12 ± 10.19	I=67.5%, C=74.2%, O=70.85%	NR	CHD, Hypertension, Hyperlipidaemi a Obesity Diabetes mellitus	TAU	Medical	Pre/Post	Medium	Pharmacist
Yiadom 2020	I=52.4 (17.7) C=53.4 (18.1)	I=50.4% C=53.4%	I: White=75.9%, Black=21.1%, Other=1%: C: White=76.2%, Black=20.7%, Other=2%	All general medicine discharges	TAU	Medical	Post	Low	Nurse
Yin 2020	I=47.5 (18.3) C=46.3 (19.0)	I=64.5% C=63.3%	NR	Nephrotic syndrome	TAU	Medical	Pre/Post	Medium	Pharmacist
You 2020	I=50.8 (9.9), C=51.3 (10.6)	I=73.6%, C=73.8%	NR	Heart Failure	TAU	Medical	Post	Low	Nurse
Zhang 2018	I=66.6 (10.5); C=65.3 (8.13); O= 65.9 (9.43)	I=50%, C=57%, O=46.2% 53.8	NR	MI, Angina	TAU	Medical	Pre/Post/ Bridging	High	Nurse

© 2023 Tyler N et al. *JAMA Network Open.*

Appendix 4: Risk of Bias Assessment

Study	Allocation concealment	Intention-to-treat (yes/no)	Attrition (<5%, 5-20%, or >20%)	Selection reporting bias	Total score	RoB Grade
Adamuz 2015	3	3	2	2	10	Low
Arendts	1	3	2	1	7	Low
Auger 2018	3	3	2	2	10	Low
Balaban 2008	1	1	2	2	6	High
Barfar 2017	3	3	1	2	9	Low
Barker 2020	1	3	0	1	5	High
Bawazeer 2021	3	2	1	2	7	Low
Bell 2016	3	3	2	2	10	Low
Biese 2018	3	3	2	1	9	Low
Bloodworth 2019	3	3	2	1	9	Low
Bloom 2019	1	3	0	2	6	High
Blum	1	1	2	2	6	High
Bonetti 2018	3	3	0	0	6	High
Bonnet Zamponi	1	2	0	2	5	High
Bonsack 2016	3	3	1	1	8	Low
Bouchard 2021	3	2	2	2	9	Low
Bruhwiler 2019	1	1	1	2	5	High
Chen 2018	3	3	1	2	9	Low
Chen 2019	1	3	2	2	8	Low
Coskun 2021	2	1	2	2	7	Low
Dalal 2018	1	1	0	2	4	High
Danielsen 2020	3	3	2	1	9	Low
Dawes	3	1	2	2	8	Low

© 2023 Tyler N et al. *JAMA Network Open.*

Dawson 2021	3	3	0	1	7	Low
Devore 2021	3	1	1	2	7	Low
Dhalla 2014	3	3	2	2	10	Low
Edey 2019	1	1	2	1	5	High
Englander	1	3	0	2	6	High
Evans 1993	1	1	2	2	6	High
Farris 2014	3	1	2	2	8	Low
Finlayson 2018	3	3	1	1	8	Low
Finn 2020	1	3	2	2	8	Low
Gardner 2020	2	1	0	2	5	High
Gharadi-Vasfi 2015	3	3	2	2	10	Low
Gilbert 2021	3	3	2	2	10	Low
Gillard 2022	2	3	2	2	9	Low
Gillespie 2009	3	1	1	2	7	Low
Goldman 2014	3	3	1	2	9	Low
Graabaek 2018	3	3	1	2	9	Low
Gurwitz 2014	3	3	0	1	7	Low
Habib 2021	2	3	0	2	7	Low
Hanrahan	3	1	1	2	7	Low
Harrison 2002	3	3	1	2	9	Low
Harvey 2014	3	3	1	2	9	Low
Heaton 2019	3	3	2	2	10	Low
Hegelund 2019	2	3	0	1	6	High
Hegelund 2020	1	3	0	2	6	High
Hengartner 2016	3	3	1	1	8	Low
Hosein Abadi 2020	3	3	1	2	9	Low
Hu 2020	3	1	1	2	7	Low
Indraratna 2022	1	3	2	2	8	Low
Jack 2009	3	3	0	2	8	Low

© 2023 Tyler N et al. *JAMA Network Open.*

Jayaram 2022	2	3	2	2	9	Low
Jennings 2014	3	1	2	1	7	Low
Karaoui 2020	1	3	1	2	7	Low
Kennedy 1987	3	1	0	1	5	High
Ko 2017	3	3	0	2	8	Low
Kowalkowski 2022	1	1	0	1	3	High
Kripalani 2012	3	3	2	2	10	Low
Lainscak 2013	3	3	1	2	9	Low
Laramee 2003	1	1	1	2	5	High
Latour	1	3	0	1	5	High
Lavesen 2016	1	1	0	1	3	High
Lea 2020	3	3	2	2	10	Low
Legrain 2011	3	3	2	2	10	Low
Lembeck 2019	3	3	2	2	10	Low
Levine 2018	3	1	1	2	7	Low
Li 2014	3	3	1	1	8	Low
Li 2018	3	3	1	2	9	Low
Lin	1	3	2	2	8	Low
Lindhardt 2019	3	1	1	1	6	High
Liu 2020	3	3	1	2	9	Low
Lockwood 2019	3	3	0	2	8	Low
Magny-Normilus 2021	1	3	2	2	8	Low
McWilliams 2019	2	3	0	2	7	Low
Mehta 2020	3	3	1	2	9	Low
Meisinger	3	3	1	1	8	Low
Moher 1992	1	1	2	1	5	High
Naylor 1994	1	3	0	1	5	High
Nazareth 2001	3	1	0	2	6	High
Negarandeh 2019	3	1	1	2	7	Low

© 2023 Tyler N et al. *JAMA Network Open.*

Nguyen 2018	3	1	0	2	6	High
Noel 2020	3	1	1	2	7	Low
O'Connell 2018	3	3	1	2	9	Low
Odeh	3	3	1	2	9	Low
Ong 2016	3	3	0	2	8	Low
Oscalices 2019	3	2	2	0	7	Low
Ougrin 2020	1	3	2	2	8	Low
Pardessus 2002	1	1	0	0	2	High
Parsons 2018	1	3	0	1	5	High
Parsons 2020	3	3	1	2	9	Low
Piette 2020	3	3	1	1	8	Low
Pourrat 2020	3	3	2	2	10	Low
Qian 2019	1	1	0	1	3	High
Ravn-Nielson 2018	3	3	2	2	10	Low
Reynolds 2004	3	1	0	1	5	High
Rich 1993	1	1	0	2	4	High
Rich 1995	3	3	0	0	6	High
Ritchie 2016	1	3	1	2	7	Low
Sahota 2017	1	3	2	2	8	Low
Salameh 2018	3	1	2	2	8	Low
Sales	1	3	2	2	8	Low
Salmany 2018	3	1	1	1	6	High
Sanatana 2017	3	3	1	2	9	Low
Shahronki 2017	1	1	1	1	4	High
Shaw 2000	1	3	0	0	4	High
Strano 2019	3	1	2	2	8	Low
Sudas Na Ayutthaya 2018	3	3	2	2	10	Low
Thygesen 2015	3	3	2	2	10	Low
Tomita 2012	3	3	0	0	6	High

© 2023 Tyler N et al. *JAMA Network Open.*

Utens	3	3	1	2	9	Low
van der Hejiden 2019	1	3	0	1	5	High
Van Spell 2019	2	1	2	2	7	Low
Vesterby 2017	3	3	2	1	9	Low
Vinluan 2015	3	1	0	1	5	High
Webster	3	3	0	2	8	Low
Weiss 2019	1	3	0	2	6	High
Wong 2004	1	2	1	2	6	High
Wong 2014	3	3	1	2	9	Low
Wong 2015	3	3	1	1	8	Low
Wong 2016	3	3	2	2	10	Low
Wu 2019	1	1	1	1	4	High
Xie 2018	3	3	2	2	10	Low
Xu 2019	3	3	2	2	10	Low
Yiadom 2020	1	3	1	2	7	Low
Yin 2020	3	1	2	2	8	Low
You 2020	2	1	2	1	6	High
Zhang 2017	1	1	1	2	5	High

© 2023 Tyler N et al. *JAMA Network Open.*

Appendix 5: Inconsistency Analysis

30-day readmission

Comparison	k	prop	nma	95%-CI	direct	95%-CI	indir.	95%-CI	RoR	95%-CI	z	p-value
High:Low	1	0.11	1.23	[0.96; 1.56]	1.21	[0.58; 2.53]	1.23	[0.95; 1.59]	0.98	[0.45; 2.15]	-0.04	0.9694
High:Medium	0	0	1.18	[0.92; 1.52]	.	.	1.18	[0.92; 1.52]	.	.	.	.
High:Min	2	0.7	0.61	[0.40; 0.92]	0.85	[0.52; 1.39]	0.29	[0.13; 0.61]	2.96	[1.20; 7.29]	2.36	**0.0185**
High:UC	22	0.97	0.96	[0.80; 1.15]	0.98	[0.81; 1.17]	0.52	[0.18; 1.49]	1.87	[0.65; 5.39]	1.16	0.2481
Low:Medium	1	0.11	0.96	[0.76; 1.22]	0.74	[0.35; 1.54]	0.99	[0.77; 1.28]	0.74	[0.34; 1.62]	-0.75	0.4533
Low:Min	2	0.43	0.5	[0.32; 0.77]	0.81	[0.42; 1.56]	0.35	[0.20; 0.61]	2.32	[0.97; 5.52]	1.89	0.0584
Low:UC	29	0.97	0.78	[0.66; 0.92]	0.77	[0.65; 0.91]	1.13	[0.46; 2.78]	0.68	[0.27; 1.71]	-0.81	0.4169
Medium:Min	0	0	0.52	[0.33; 0.81]	.	.	0.52	[0.33; 0.81]	.	.	.	.
Medium:UC	25	0.98	0.81	[0.68; 0.97]	0.8	[0.67; 0.96]	1.43	[0.37; 5.44]	0.56	[0.15; 2.18]	-0.83	0.4064
Min:UC	3	0.66	1.57	[1.04; 2.37]	2.58	[1.56; 4.28]	0.6	[0.30; 1.22]	4.29	[1.80; 10.18]	3.3	**0.001**

Footnote:

k: number of studies; prop: proportion of direct evidence; nma: network meta-analysis estimate; 95%-CI: 95% confidence interval; direct: direct evidence estimates; indir.: indirect evidence estimates; RoR: Ratio Odds Ratio; z: standardised z score.

90-day readmission

Comparison	k	prop	nma	95%-CI	direct	95%-CI	indir.	95%-CI	RoR	95%-CI	z	p-value
High:Low	0	0	1.11	[0.66; 1.88]	.	.	1.11	[0.66; 1.88]	.	.	.	.
High:Medium	0	0	1.18	[0.76; 1.83]	.	.	1.18	[0.76; 1.83]	.	.	.	.
High:UC	17	1	0.71	[0.56; 0.91]	0.71	[0.56; 0.91]	.	.	.	.	.	.
Low:Medium	0	0	1.06	[0.59; 1.92]	.	.	1.06	[0.59; 1.92]	.	.	.	.
Low:UC	7	1	0.64	[0.40; 1.02]	0.64	[0.40; 1.02]	.	.	.	.	.	.
Medium:UC	11	1	0.6	[0.42; 0.87]	0.6	[0.42; 0.87]	.	.	.	.	.	.

© 2023 Tyler N et al. *JAMA Network Open.*

180-day readmission

Comparison	k	prop	nma	95%-CI	direct	95%-CI	indir.	95%-CI	RoR	95%-CI	z	p-value
High:Low	0	0	1.77	[1.11; 2.81]	.	.	1.77	[1.11; 2.81]	.	.	.	.
High:Medium	0	0	1.31	[0.84; 2.05]	.	.	1.31	[0.84; 2.05]	.	.	.	.
High:Min	1	0.36	1.16	[0.76; 1.76]	0.73	[0.36; 1.48]	1.49	[0.88; 2.50]	0.49	[0.21; 1.18]	-1.58	0.114
High:UC	14	0.98	0.76	[0.60; 0.97]	0.79	[0.62; 1.00]	0.21	[0.04; 0.97]	3.8	[0.80; 18.14]	1.67	0.0942
Low:Medium	0	0	0.74	[0.43; 1.29]	.	.	0.74	[0.43; 1.29]	.	.	.	.
Low:Min	1	0.45	0.65	[0.40; 1.08]	0.87	[0.41; 1.84]	0.52	[0.26; 1.02]	1.66	[0.61; 4.57]	0.99	0.3239
Low:UC	5	0.94	0.43	[0.29; 0.65]	0.42	[0.28; 0.64]	0.62	[0.12; 3.11]	0.68	[0.13; 3.57]	-0.46	0.6457
Medium:Min	1	0.47	0.88	[0.55; 1.43]	1.1	[0.55; 2.22]	0.72	[0.37; 1.40]	1.53	[0.58; 4.01]	0.86	0.389
Medium:UC	9	0.92	0.58	[0.40; 0.86]	0.54	[0.36; 0.81]	1.3	[0.33; 5.13]	0.42	[0.10; 1.75]	-1.19	0.2339
Min:UC	3	0.8	0.66	[0.45; 0.96]	0.66	[0.44; 1.01]	0.65	[0.28; 1.49]	1.02	[0.40; 2.60]	0.05	0.9606

ED visit

Comparison	k	prop	nma	95%-CI	direct	95%-CI	indir.	95%-CI	RoR	95%-CI	z	p-value
High:Low	0	0	1.2	[0.78; 1.84]	.	.	1.2	[0.78; 1.84]	.	.	.	.
High:Medium	0	0	0.82	[0.56; 1.20]	.	.	0.82	[0.56; 1.20]	.	.	.	.
High:Min	2	0.77	0.93	[0.51; 1.69]	0.93	[0.47; 1.84]	0.93	[0.26; 3.28]	1.01	[0.24; 4.23]	0.01	0.9943
High:UC	17	1	0.82	[0.63; 1.06]	0.82	[0.63; 1.06]	.	.	.	.	.	.
Low:Medium	0	0	0.68	[0.44; 1.06]	.	.	0.68	[0.44; 1.06]	.	.	.	.
Low:Min	0	0	0.77	[0.39; 1.54]	.	.	0.77	[0.39; 1.54]	.	.	.	.
Low:UC	9	1	0.68	[0.48; 0.95]	0.68	[0.48; 0.95]	.	.	.	.	.	.
Medium:Min	0	0	1.13	[0.59; 2.19]	.	.	1.13	[0.59; 2.19]	.	.	.	.
Medium:UC	16	1	0.99	[0.76; 1.31]	0.99	[0.76; 1.31]	.	.	.	.	.	.
Min:UC	2	0.8	0.88	[0.48; 1.59]	0.88	[0.45; 1.71]	0.87	[0.23; 3.31]	1.01	[0.23; 4.48]	0.01	0.9913

© 2023 Tyler N et al. *JAMA Network Open.*

Mortality

Comparison	k	prop	nma	95%-CI	direct	95%-CI	indir.	95%-CI	RoR	95%-CI	z	p-value
High:Low	1	0.19	1.09	[0.91; 1.29]	1	[0.67; 1.49]	1.11	[0.91; 1.34]	0.9	[0.58; 1.41]	-0.44	0.6582
High:Medium	0	0	1.14	[0.92; 1.42]	.	.	1.14	[0.92; 1.42]	.	.	.	.
High:Min	1	0.42	1.32	[1.00; 1.74]	1.37	[0.90; 2.10]	1.28	[0.89; 1.84]	1.07	[0.61; 1.87]	0.24	0.8077
High:UC	12	0.97	0.97	[0.87; 1.09]	0.98	[0.88; 1.10]	0.74	[0.38; 1.43]	1.33	[0.68; 2.60]	0.84	0.4033
Low:Medium	1	0.08	1.05	[0.83; 1.32]	0.84	[0.37; 1.92]	1.07	[0.84; 1.36]	0.78	[0.33; 1.85]	-0.56	0.5757
Low:Min	2	0.63	1.22	[0.92; 1.60]	1.32	[0.93; 1.86]	1.06	[0.67; 1.67]	1.25	[0.71; 2.21]	0.76	0.4456
Low:UC	17	0.95	0.9	[0.78; 1.03]	0.9	[0.78; 1.04]	0.83	[0.45; 1.52]	1.08	[0.58; 2.02]	0.26	0.7967
Medium:Min	1	0.27	1.16	[0.85; 1.57]	1.22	[0.67; 2.21]	1.14	[0.79; 1.63]	1.07	[0.53; 2.15]	0.2	0.8448
Medium:UC	16	0.96	0.86	[0.71; 1.03]	0.85	[0.70; 1.03]	1.07	[0.39; 2.88]	0.79	[0.29; 2.19]	-0.44	0.657
Min:UC	3	0.76	0.74	[0.57; 0.96]	0.81	[0.60; 1.09]	0.55	[0.32; 0.95]	1.46	[0.79; 2.70]	1.2	0.2308

Intervention Uptake

Comparison	k	prop	nma	95%-CI	direct	95%-CI	indir.	95%-CI	RoR	95%-CI	z	p-value
High:Low	1	0.06	1.02	[0.74; 1.39]	1.59	[0.43; 5.82]	0.99	[0.71; 1.37]	1.61	[0.42; 6.13]	0.69	0.4873
High:Medium	0	0	0.95	[0.70; 1.28]	.	.	0.95	[0.70; 1.28]	.	.	.	.
High:Min	1	0.41	0.49	[0.21; 1.15]	0.23	[0.06; 0.86]	0.84	[0.28; 2.53]	0.27	[0.05; 1.53]	-1.48	0.1395
High:UC	41	0.98	0.92	[0.74; 1.14]	0.92	[0.74; 1.14]	1.03	[0.19; 5.57]	0.89	[0.16; 4.90]	-0.13	0.8965
Low:Medium	4	0.3	0.93	[0.69; 1.26]	1.01	[0.58; 1.74]	0.9	[0.63; 1.29]	1.11	[0.58; 2.14]	0.32	0.7511
Low:Min	1	0.42	0.48	[0.21; 1.14]	0.86	[0.23; 3.23]	0.32	[0.11; 0.98]	2.67	[0.47; 15.06]	1.12	0.2647
Low:UC	35	0.93	0.91	[0.72; 1.15]	0.89	[0.70; 1.14]	1.14	[0.46; 2.85]	0.78	[0.30; 2.02]	-0.5	0.6151
Medium:Min	0	0	0.52	[0.22; 1.23]	.	.	0.52	[0.22; 1.23]	.	.	.	.
Medium:UC	40	0.96	0.97	[0.78; 1.20]	0.98	[0.79; 1.22]	0.72	[0.25; 2.12]	1.36	[0.45; 4.08]	0.55	0.5836
Min:UC	2	0.75	1.87	[0.81; 4.32]	1.47	[0.56; 3.86]	3.84	[0.73; 20.28]	0.38	[0.06; 2.62]	-0.98	0.3276

© 2023 Tyler N et al. *JAMA Network Open.*

Appendix 6: Publication bias and funnel plots

Comparison adjusted funnel plots.

30-day readmission

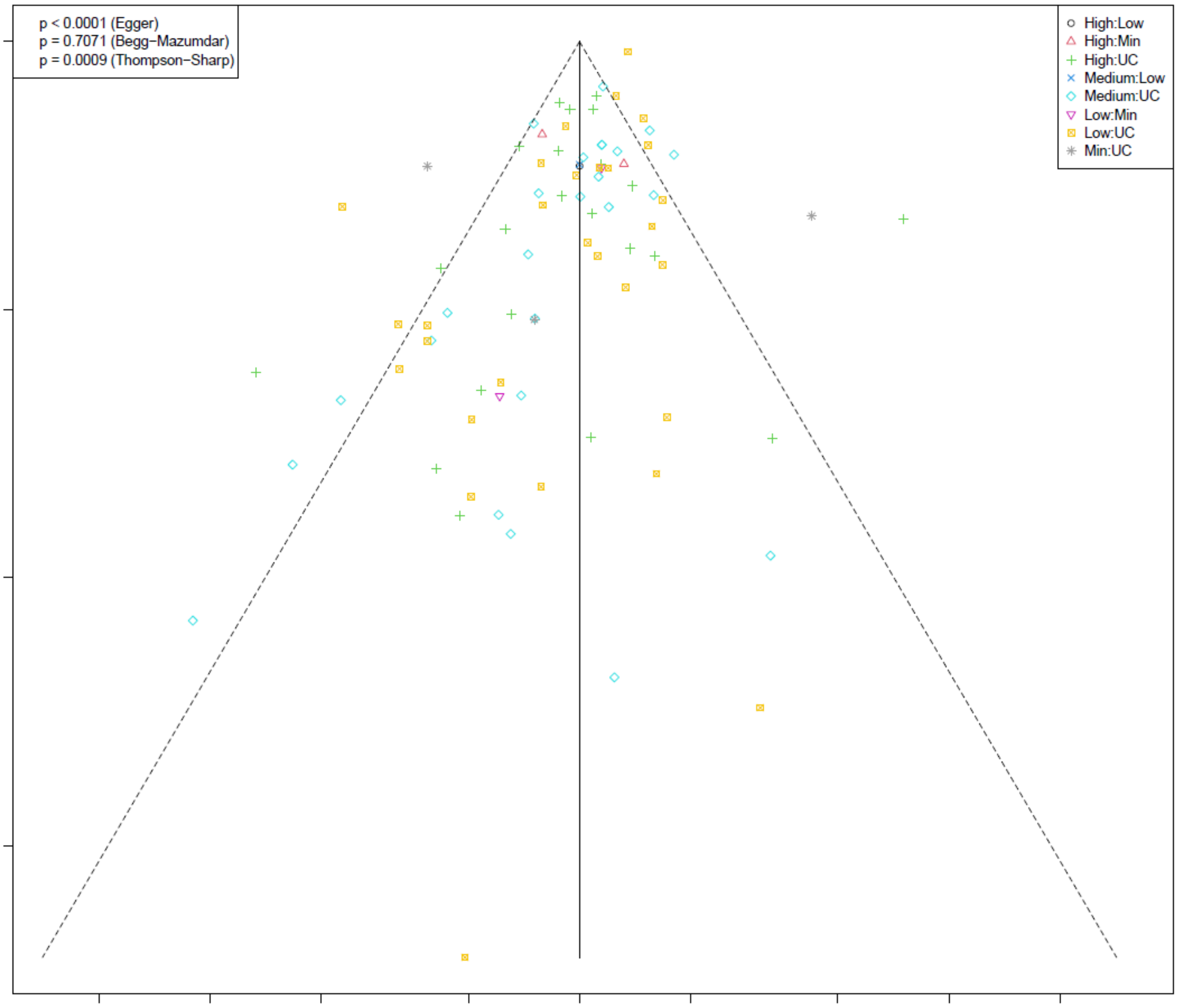

© 2023 Tyler N et al. *JAMA Network Open.*

90-day readmission

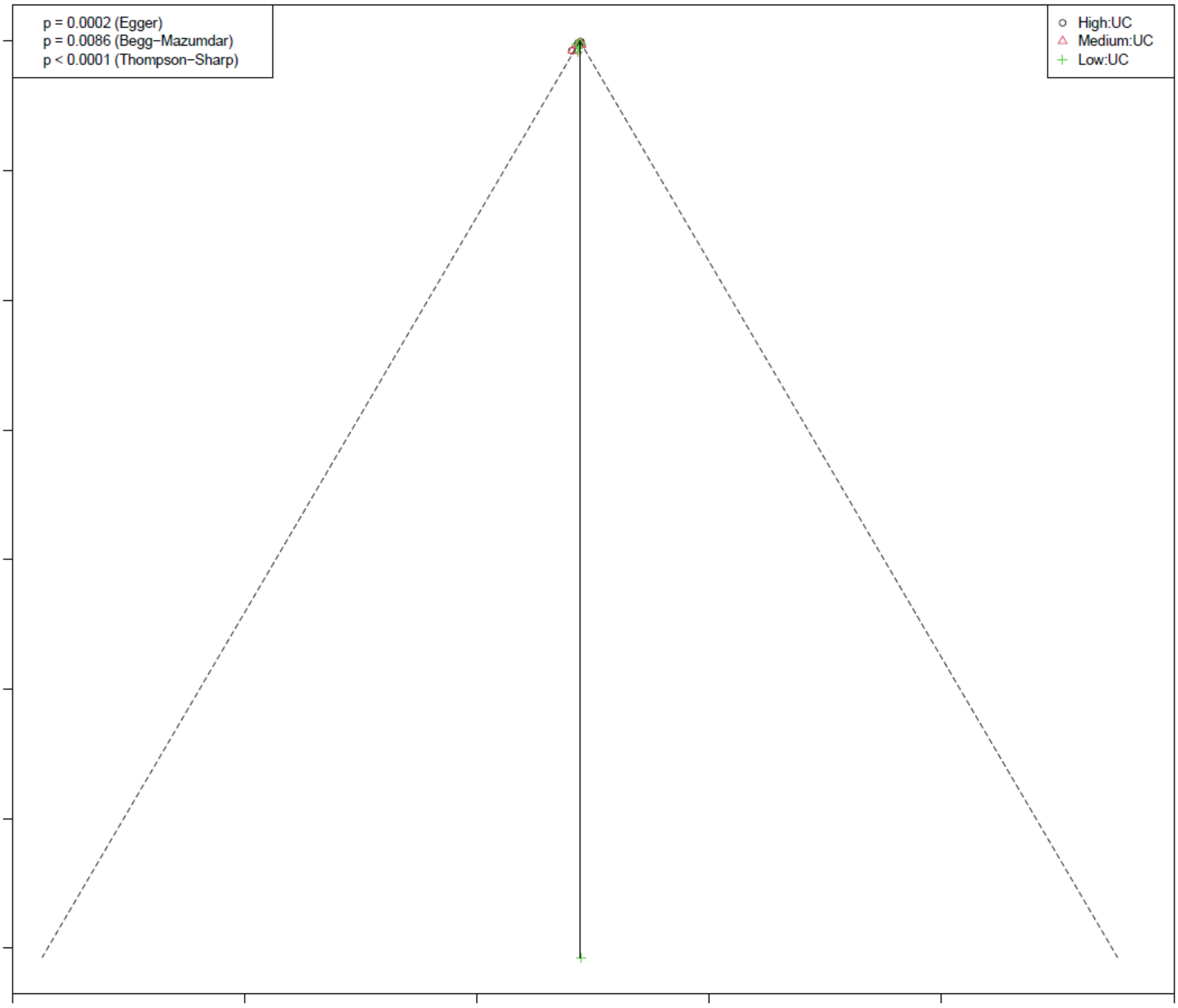

© 2023 Tyler N et al. *JAMA Network Open.*

180-day readmission

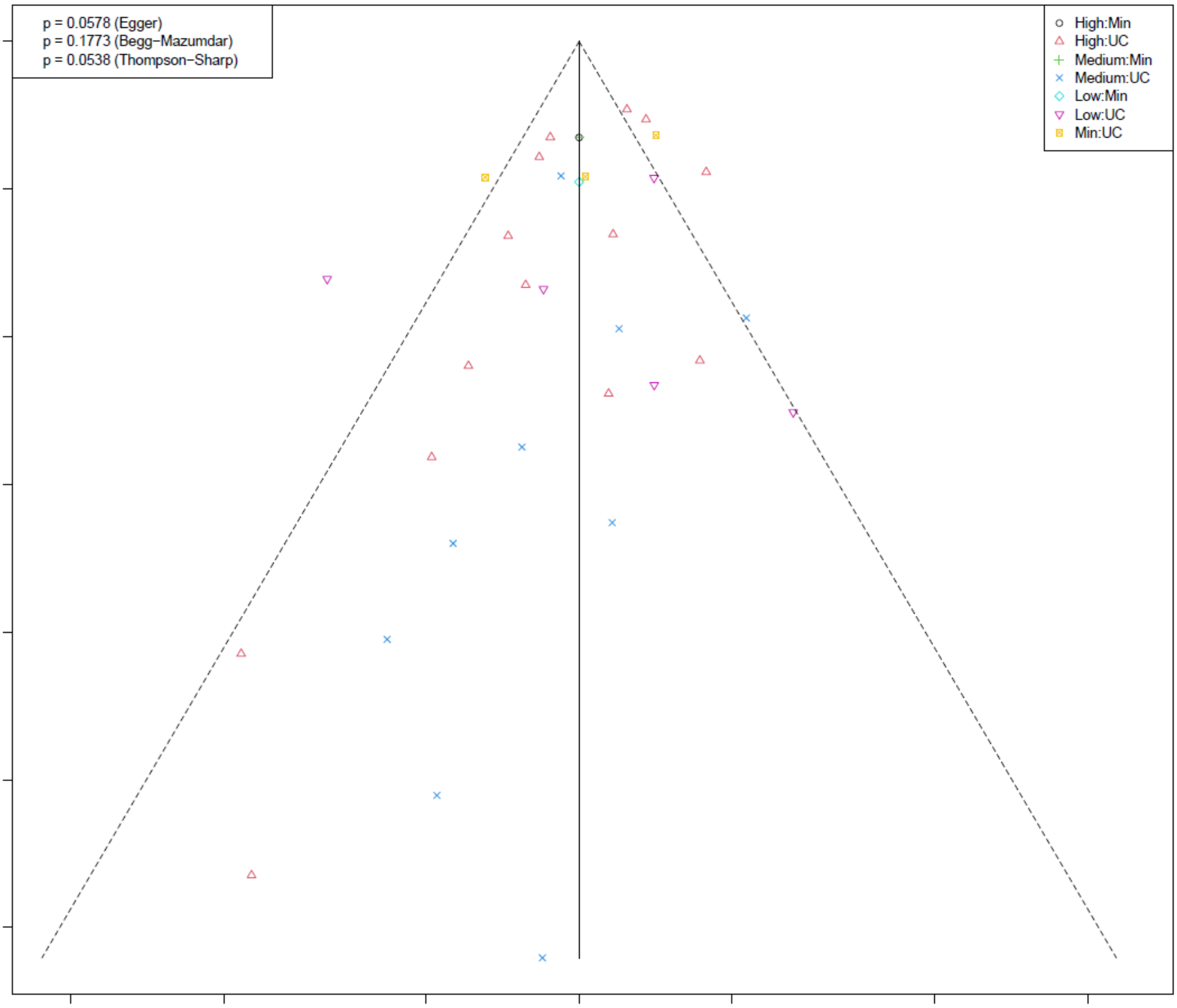

© 2023 Tyler N et al. *JAMA Network Open.*

ED visit

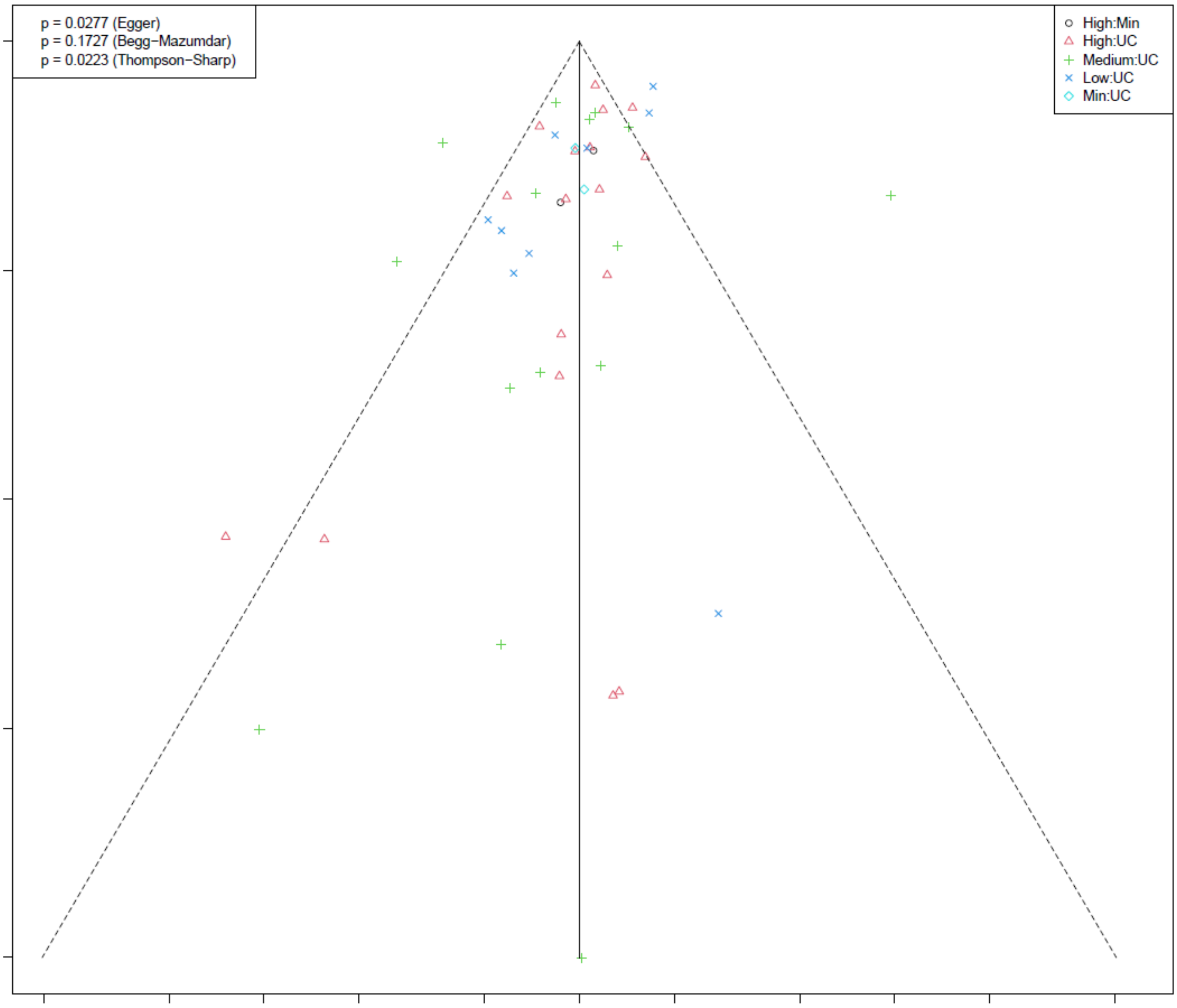

© 2023 Tyler N et al. *JAMA Network Open.*

Mortality

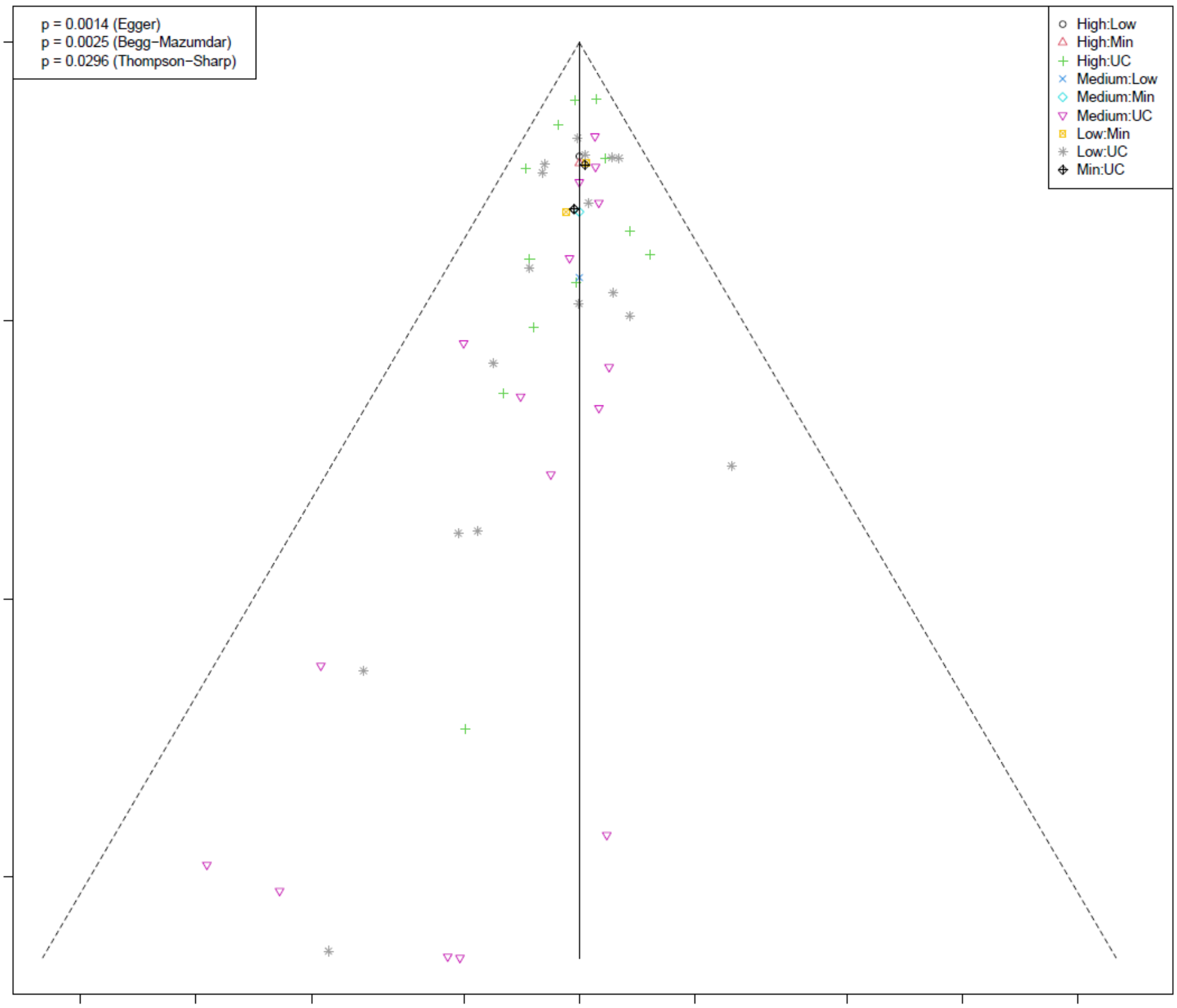

© 2023 Tyler N et al. *JAMA Network Open.*

Intervention Uptake

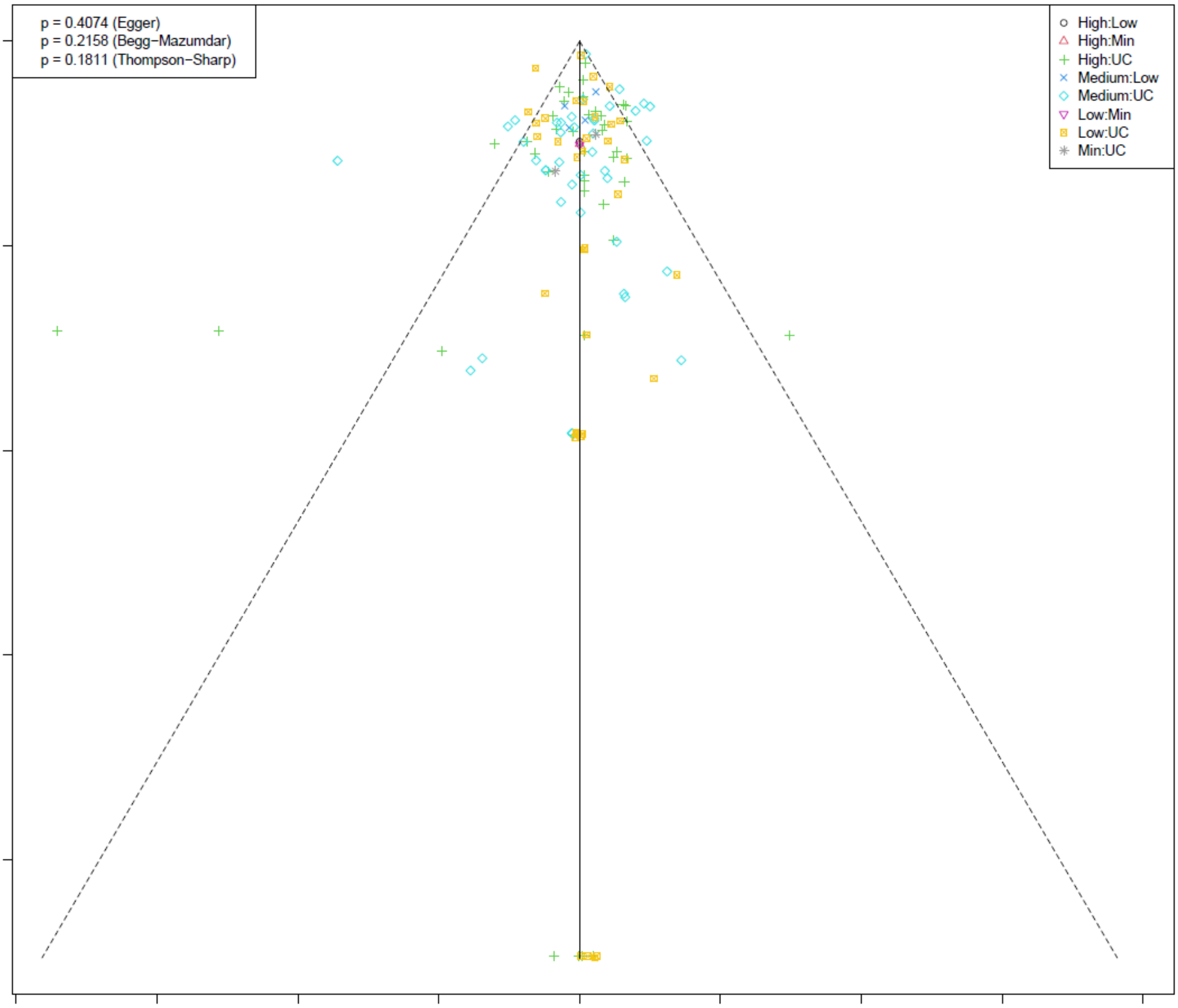

© 2023 Tyler N et al. *JAMA Network Open.*

Appendix 7: Meta-Regressions

30-day readmission

Network meta-regression results for the primary outcome 'readmissions at 30-days' based on complexity of the discharge intervention. "Results are as log OR".

		High vs. UC (n=22)	Medium vs. UC (n=25)	Low vs. UC (n=29)
**Variable**	**Category**	**Beta (95% CI), P-value**		
**Study and patient characteristics**				
Age	< 45 yrs.	Ref.	Ref.	Ref.
	45 to 59 yrs.	-1.67 (-3.45, 0.10), p=0.064	0.03 (-1.05, 1.12), p=0.953	1.23 (-0.25, 2.71), p=0.103
	60to 79 yrs.	-1.08 (-2.79, 0.62), p=0.214	0.19 (-0.54, 0.92), p=0.609	0.72 (-0.78, 2.21), pp=0.331
	≥ 80 yrs.	-1.54 (-3.35, 0.27), p=0.095	-1.03 (-2.65, 0.60), p=0.215	1.25 (-0.41, 2.91), p=0.139
	NR	-1.31 (-3.30, 0.68), p=0.196	-0.16 (-1.18, 0.87), p=0.766	1.40 (-0.46, 3.26), p=0.140
Sex	Female (≥55%)	Ref.	Ref.	Ref.
	Male (≥55%)	0.11 (-0.80, 1.01), p=0.818	-0.03 (-0.59, 0.53), p=0.907	-0.41 (-1.18, 0.24), p=0.237
	Mixed	0.28 (-0.39, 0.94), p=0.414	0.34 (-0.22, 0.89), p=0.223	0.11 (-0.35, 0.57), p=0.644
	NR	0.36 (-0.88, 1.60), p=0.565	-0.13 (-1.25, 1.00), p=0.823	-0.10 (-1.02, 0.82), p=0.833
OECD	No	Ref.	Ref.	Ref.
	Yes	0.55 (-0.59, 1.68), p=0.345	0.62 (-0.01, 1.24), p=0.056	0.05 (-0.65, 0.75), p=0.881
	NR	NA		
WHO region	Americas	Ref.	Ref.	Ref.
	Europe	0.51 (-0.11, 1.13), p=0.108	-0.04 (-0.58, 0.50), p=0.874	0.03 (-0.51, 0.57), p=0.907
	Western pacific	-0.47 (-1.61, 0.67), p=0.417	-0.84 (-1.47, -0.20), **p=0.010**	-0.08 (-1.41, 0.80), p=0.846
	South-east Asia	NA	NA	NA
	Africa	NA	NA	0.10 (-0.98, 0.75), p=0.756
	Eastern Mediterranean	NA	NA	0.53 (-0.84, 1.90), p=0.450
	NR	NA	NA	NA
Patient Complexity	Low Complexity	Ref.	Ref.	Ref.
	High Complexity	NA	0.12 (-0.46, 0.69), p=0.692	-0.14 (-0.61, 0.33), p=0.557
**Intervention Characteristics**				
Delivery professional	Nurse	Ref.	Ref.	Ref.
	Other HCP	0.14 (-0.48, 0.77), p=0.649	-0.22 (-0.78, 0.35), p=0.455	-0.18 (-0.60, 0.24), p=0.397
	MDT	-0.37 (-0.85, 0.10), p=0.121	0.01 (-0.53, 0.56), p=0.962	-0.38 (-0.91, 0.15), p=0.162
	Social/PC	NA	-0.30 (-1.36, 0.76), p=0.580	-1.20 (-2.68, 0.28), p=0.112

© 2023 Tyler N et al. *JAMA Network Open.*

	Other	NA	-0.36 (-1.22, 0.50), p=0.410	-1.51 (-2.47, -0.56), **p=0.002**
Population	Medical	Ref.	Ref.	Ref.
	MH	NA	NA	-1.02 (-2.52, 0.48), p=0.182
Inc. Medication Reconciliation	No	Ref.	Ref.	Ref.
	Yes	-0.04 (-0.47, 0.27), p=0.851	0.11 (-0.32, 0.54), p=0.510	-0.07 (-0.53, 0.37), p=0.742
**Sensitivity analysis**				
Quality assessment	Low (>6)	Ref.	Ref.	Ref.
	High (≤6)	-0.74 (-1.74, 0.53), p=0.473	-0.34 (-1.08, 0.19), p=0.173	-0.88 (-2.47, -0.09), **p=0.042**

© 2023 Tyler N et al. *JAMA Network Open.*

90-day readmission

Network meta-regression results for the primary outcome 'readmissions at 90-days' based on complexity of the discharge intervention

		High vs. UC (n=17)	Medium vs. UC (n=11)	Low vs. UC (n=7)
**Variable**	**Category**	**Beta (95% CI), P-value**		
**Study and patient characteristics**				
Age	< 45 yrs.	Ref.	Ref.	Ref.
	45 to 59 yrs.	NA	-0.31 (-2.59, 1.97), p=0.790	-0.53 (-2.12, 1.07), p=0.517
	60to 79 yrs.	0.29 (-0.64, 1.21), p=0.544	0.15 (-1.30, 1.61), p=0.836	0.87 (-0.38, 2.14), p=0.174
	≥ 80 yrs.	0.01 (-1.27, 1.29), p=0.983	NA	NA
	NR	NA	NA	NA
Sex	Female (≥55%)	Ref.	Ref.	Ref.
	Male (≥55%)	0.71 (-0.02, 1.43), p=0.057	0.21 (-1.27, 1.69), p=0.777	-1.75 (-3.02, 0.48), **p=0.007**
	Mixed	0.89 (0.30, 1.48), **p=0.003**	0.29 (-1.96, 2.54), p=0.801	0.09 (-0.91, 1.09), p=0.860
	NR	0.92 (-0.12, 1.97), p=0.084	NA	NA
OECD	No	Ref.	Ref.	Ref.
	Yes	0.43 (-0.54, 1.40), p=0.382	-0.04 (-1.12, 1.05), p=0.949	1.39 (0.35, 2.44), **p=0.009**
	NR	NA	NA	NA
WHO region	Americas	Ref.	Ref.	Ref.
	Europe	-0.54 (-1.36, 0.28), p=0.199	-0.10 (-2.47, 2.27), p=0.935	0.57 (-0.70, 1.84), p=0.381
	Western pacific	-0.22 (-1.44, 0.99), p=0.719	-0.29 (-2.70, 2.13), p=0.816	-1.78 (-3.21, -0.035), **p=0.015**
	South-east Asia	NA	NA	-0.06 (-2.26, 1.14), p=0.521
	Africa	NA	NA	NA
	Eastern Mediterranean	-1.02 (-2.66, 0.63), p=0.224	-0.10 (-2.75, 2.55), p=0.942	-1.57 (-4.06, 0.92), p=0.216
	NR	NA	NA	NA
Patient Complexity	Low Complexity	Ref.	Ref.	Ref.
	High Complexity	NA	NA	-0.06 (-2.23, 2.12), p=0.959
**Intervention Characteristics**				
Delivery professional	Nurse	Ref.	Ref.	Ref.
	Other HCP	-0.03 (-0.99, 0.93), p=0.950	1.23 (-1.01, 3.47), p=0.283	1.18 (-0.85, 3.21), p=0.254
	MDT	-0.18 (-1.01, 0.66), p=0.676	-0.31 (-2.48, 1.85), p=0.776	1.79 (-0.51, 3.06), **p=0.006**
	Social/PC	0.37 (-1.11, 1.84), p=0.624	NA	1.23 (-0.59-3.04), p=0.18
	Other	-0.91 (-2.76, 0.94), p=0.336	0.39 (-1.17, 1.95), p=0.625	2.30 (0.61, 3.99), **p=0.008**
Population	Medical	Ref.	Ref.	Ref.

© 2023 Tyler N et al. *JAMA Network Open.*

	MH	NA	0.37 (-1.46, 2.20), p=0.695	0.06 (-2.24, 2.12), p=0.955
Inc. Medication Reconciliation	No	Ref.	Ref.	Ref.
	Yes	0.07 (0.84, -0.58), p=0.840	1.06 (-0.65, 2.78), p=0.225	-1.77 (-3.18, 0.35), **p=0.015**
**Sensitivity analysis**				
Quality assessment	Low (>6)	Ref.	Ref.	Ref.
	High (≤6)	-0.94 (-2.10, 0.79), p=0.648	0.05 (-0.97, 1.08), p=0.641	-0.28 (-0.67, 0.48), p=0.189

© 2023 Tyler N et al. *JAMA Network Open.*

Mortality

Network meta-regression results for the outcome 'mortality combined' based on complexity of the discharge intervention

		High vs. UC (n=12)	Medium vs. UC (n=16)	Low vs. UC (n=17)
**Variable**	**Category**	**Beta (95% CI), P-value**		
**Study and patient characteristics**				
Age	< 45 yrs.	Ref.	Ref.	Ref.
	45 to 59 yrs.	NA	-1.73 (-4.29, 0.893), p=0.185	NA
	60to 79 yrs.	0.28 (-1.15, 1.72), p=0.698	-0.36 (-1.90-1.18), p=0.65	0.01 (-0.69, 0.71), p=0.979
	≥ 80 yrs.	0.13 (-1.41, 1.68), p=0.866	0.08 (-1.84, 1.68), p=0.9275	0.37 (-0.46, 1.19), p=0.382
	NR	NA	NA	0.46 (-1.03, 1.95), p=0.544
Sex	Female (≥55%)	Ref.	Ref.	Ref.
	Male (≥55%)	0.56 (-0.55, 1.66), p=0.324	0.13 (-0.89, 1.14), p=0.807	0.01 (-0.90, 0.70), p=0.800
	Mixed	0.33 (-0.42, 1.08), p=0.391	0.37 (-0.39, 1.14), p=0.341	-0.07 (-0.74, 0.59), p=0.830
	NR	0.64 (-0.68, 1.96), p=0.340	NA	NA
OECD	No	Ref.	Ref.	Ref.
	Yes	NA	0.29 (-0.57, 1.16), p=0.503	0.29 (-0.49, 1.10), p=0.463
	NR	NA	NA	NA
WHO region	Americas	Ref.	Ref.	Ref.
	Europe	0.05 (-0.52, 0.61), p=0.878	0.22 (-0.54, 0.98), p=0.571	0.27 (-0.44, 0.99), p=0.455
	Western pacific	NA	0.25 (-0.89, 1.39), p=0.666	0.02 (-0.90, 0.94), p=0.960
	South-east Asia	NA	NA	NA
	Africa	NA	NA	0.19 (-0.82, 1.19), p=0.716
	Eastern Mediterranean	NA	-1.28 (-3.44, 0.87), p=0.243	-0.42 (-2.34, 1.51), p=0.673
	NR	NA	NA	NA
Patient Complexity	Low Complexity	Ref.	Ref.	Ref.
	High Complexity	NA	0.19 (-0.57, 0.95), p=0.629	0.02 (-0.60, 0.64), p=0.941
**Intervention Characteristics**				
Delivery professional	Nurse	Ref.	Ref.	Ref.
	Other HCP	-0.01 (-0.72, 0.70), p=0.979	0.13 (-0.65, 0.92), p=0.746	-0.10 (-0.71, 0.51), p=0.747
	MDT	-0.14 (-0.81, 0.54), p=0.695	0.23 (-0.86, 1.33), p=0.671	-0.47 (-1.43, 0.49), p=0.340
	Social/PC	-0.04 (-1.40, 1.31), p=0.949	0.12 (-1.31, 1.54), p=0.873	NA
	Other	-0.71 (-2.93, 1.51), p=0.529	NA	-0.58 (-2.12, 0.97), p=0.464
Population	Medical	Ref.	Ref.	Ref.

© 2023 Tyler N et al. *JAMA Network Open.*

	MH	NA	0.36 (-1.18, 1.89), p=0.648	NA
Inc. Medication Reconciliation	No	Ref.	Ref.	Ref.
	Yes	-0.06 (-0.64, 0.31), p=0.849	-0.02 (-0.67, 0.70), p=0.960	-0.05 (-0.62, 0.52), p=0.868
**Sensitivity analysis**				
Quality assessment	Low (>6)	Ref.	Ref.	Ref.
	High (≤6)	-0.37 (-0.94, 0.59), p=0.376	-1.08 (-1.97, 0.54), p=0.489	-0.78 (-1.84, 0.87), p=0.355

© 2023 Tyler N et al. *JAMA Network Open.*

Intervention Uptake

Network meta-regression results for the outcome 'uptake' based on complexity of the discharge intervention

		High vs. UC (n=41)	Medium vs. UC (n=40)	Low vs. UC (n=35)
**Variable**	**Category**	**Beta (95% CI), P-value**		
**Study and patient characteristics**				
Age	< 45 yrs.	Ref.	Ref.	Ref.
	45 to 59 yrs.	-0.12 (-1.68, 1.43), p=0.875	-0.28 (-1.57, 1.01), p=0.671	-0.72 (-2.01, 0.58), p=0.277
	60to 79 yrs.	-0.77 (-2.15, 0.61), p=0.276	0.13 (-0.87, 1.16), p=0.788	-0.53 (-1.74, 0.68), p=0.388
	≥ 80 yrs.	0.24 (-1.58, 2.05), p=0.801	-0.23 (-1.61, 1.15), p=0.745	-0.51 (-3.07, 2.05), p=0.698
	NR	-0.97 (-4.03, 2.08), p=0.531	-0.04 (-1.62, 1.72), p=0.957	NA
Sex	Female (≥55%)	Ref.	Ref.	Ref.
	Male (≥55%)	-0.03 (-1.13, 1.18), p=0.961	0.10 (-0.57, 0.77), p=0.766	0.25 (-0.36, 0.86), p=0.416
	Mixed	-0.78 (-1.87, 0.30), p=0.160	-0.78 (-1.55, -0.02), **p=0.046**	-0.15 (-0.66, 0.37), p=0.581
	NR	-0.20 (-2.23, 1.83), p=0.847	-0.63 (-2.03, 0.77), p=0.379	-0.52 (-1.47, 0.42), p=0.278
OECD	No	Ref.	Ref.	Ref.
	Yes	-0.63 (-1.63, 0.37), p=0.215	-0.60 (-1.20, 0.01), p=0.054	-0.30 (-0.82, 0.21), p=0.247
	NR	NA	NA	NA
WHO region	Americas	Ref.	Ref.	Ref.
	Europe	0.51 (-0.52, 1.53)), p=0.334	0.33 (-0.22, 0.86), p=0.179	0.26 (-0.32, 0.84), p=0.381
	Western pacific	1.05 (-0.09, 2.20), p=0.073	0.42 (-0.15, 0.98), p=0.147	0.03 (-0.54, 0.61), p=0.919
	South-east Asia	NA	NA	0.01 (-2.73, 2.75), p=0.995
	Africa	NA	-3.86 (-5.41, -2.30), **p<0.0001**	-0.39 (-1.53, 0.75), p=0.503
	Eastern Mediterranean	0.72 (-1.51, 2.95), p=0.527	0.45 (-0.43, 1.32), p=0.320	0.88 (-0.30, 2.07), p=0.145
	NR	NA	NA	NA
Patient Complexity	Low Complexity	Ref.	Ref.	Ref.
	High Complexity	0.65 (-0.22, 1.52), p=0.143	0.60 (-0.11, 1.31), p=0.099	-0.24 (-0.73, 0.25), p=0.330
**Intervention Characteristics**				
Delivery professional	Nurse	Ref.	Ref.	Ref.
	Other HCP	-0.47 (-1.62, 0.68), p=0.423	0.45 (-0.28, 1.17), p=0.228	0.24 (-0.34, 0.82), p=0.417
	MDT	-0.61 (-1.72, 0.59), p=0.281	-0.12 (-1.11, 0.87), p=0.814	-0.12 (-0.43, 0.66), p=0.675
	Social/PC	0.40 (-1.06, 1.87), p=0.586	-0.25 (-1.39, 0.89), p=0.674	0.17 (-1.31, 1.65), p=0.822
	Other	NA	0.43 (-0.53, 1.40), p=0.384	0.23 (-0.66, 1.11), p=0.617
Population	Medical	Ref.	Ref.	Ref.

© 2023 Tyler N et al. *JAMA Network Open.*

	MH	0.40 (-1.09, 1.90), p=0.573	0.18 (-0.87, 1.24), p=0.731	0.05 (-1.39, 1.51), p=0.939
Inc. Medication Reconciliation	No	Ref.	Ref.	Ref.
	Yes	-0.81 (-1.64, 0.02), p=0.056	-0.16 (-0.35, 0.40), p=0.891	0.24 (-0.37, 0.85), p=0.437
**Sensitivity analysis**				
Quality assessment	Low (>6)	Ref.	Ref.	Ref.
	High (≤6)	-0.01 (-0.75, 0.69), p=0.499	0.28 (-0.47, 0.87), p=0.689	-0.79 (-1.49, 0.49), p=0.271

© 2023 Tyler N et al. *JAMA Network Open.*

Appendix 8: Sensitivity analysis

30-day readmissions

- Discharge process

Network Graph:

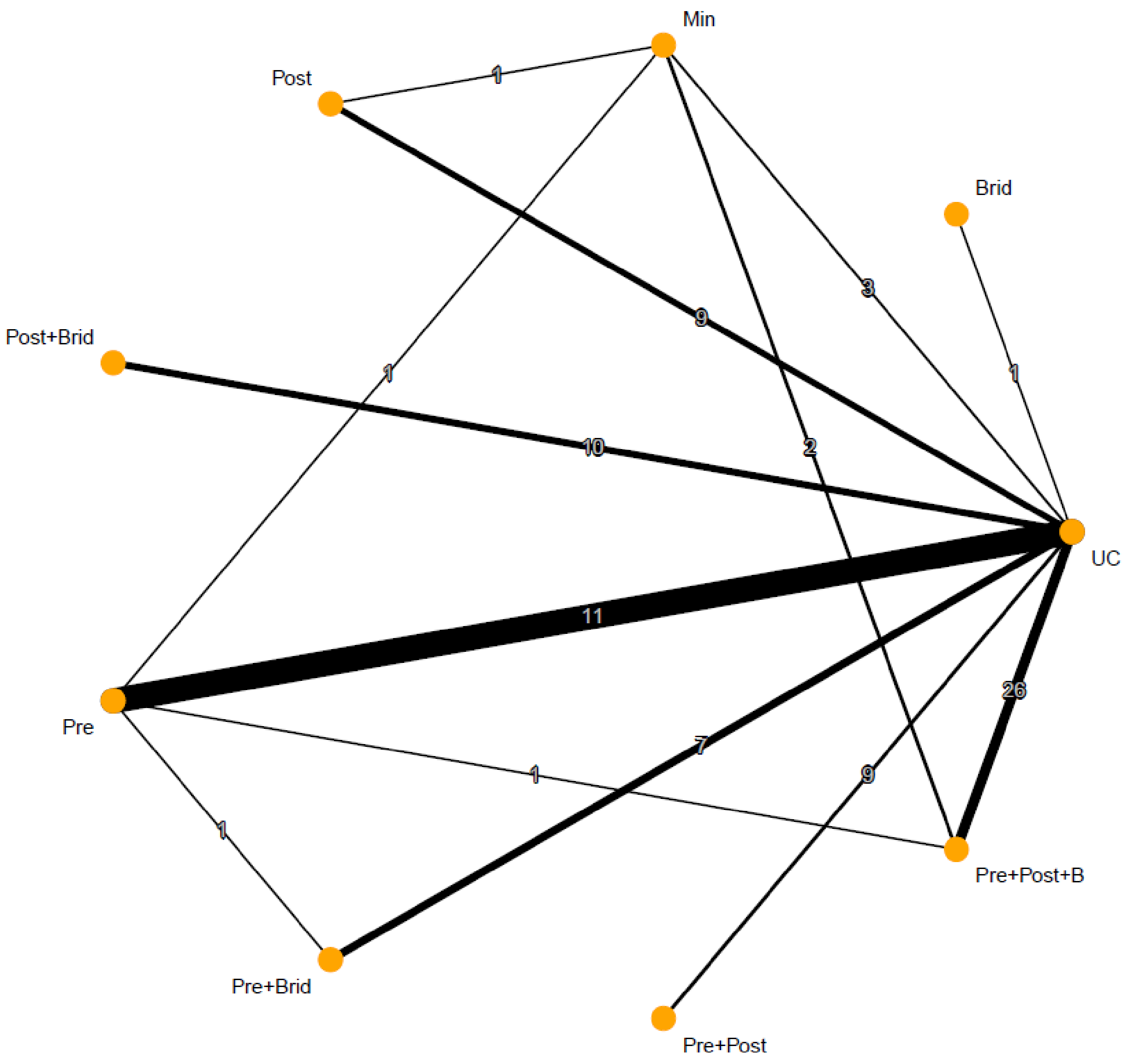

© 2023 Tyler N et al. *JAMA Network Open.*

Network meta-analysis forest plot:

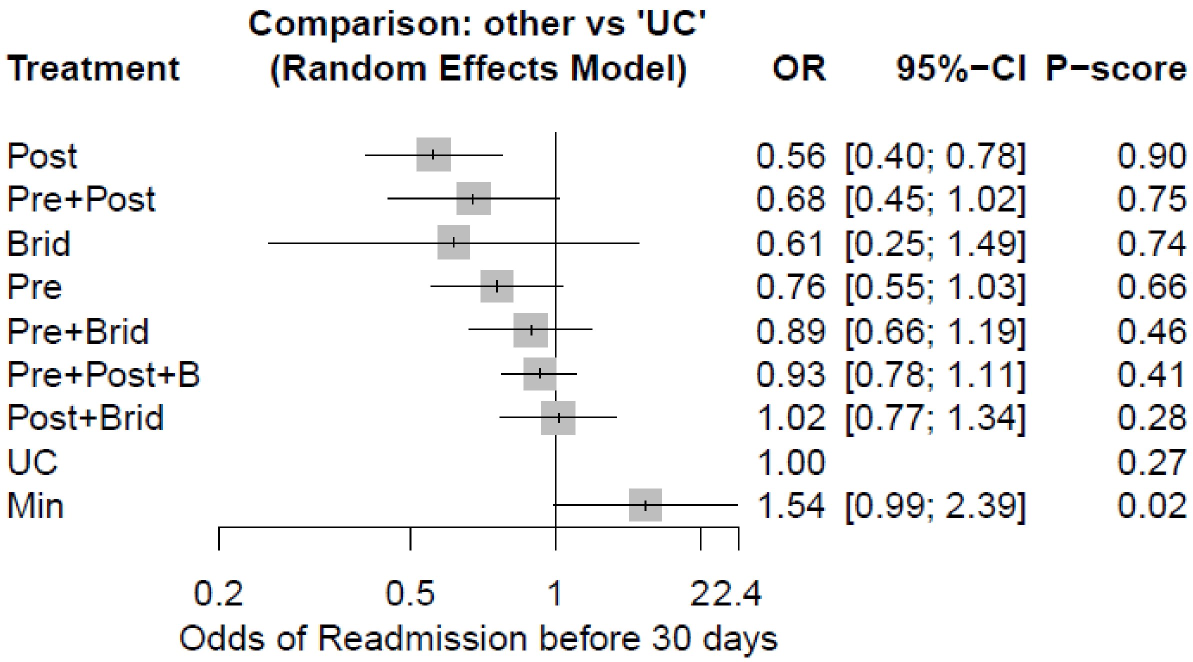

Heterogeneity:

I^2 = 63.9% [53.4%; 72.1%]

- Number of discharge stages

© 2023 Tyler N et al. *JAMA Network Open.*

Network Graph:

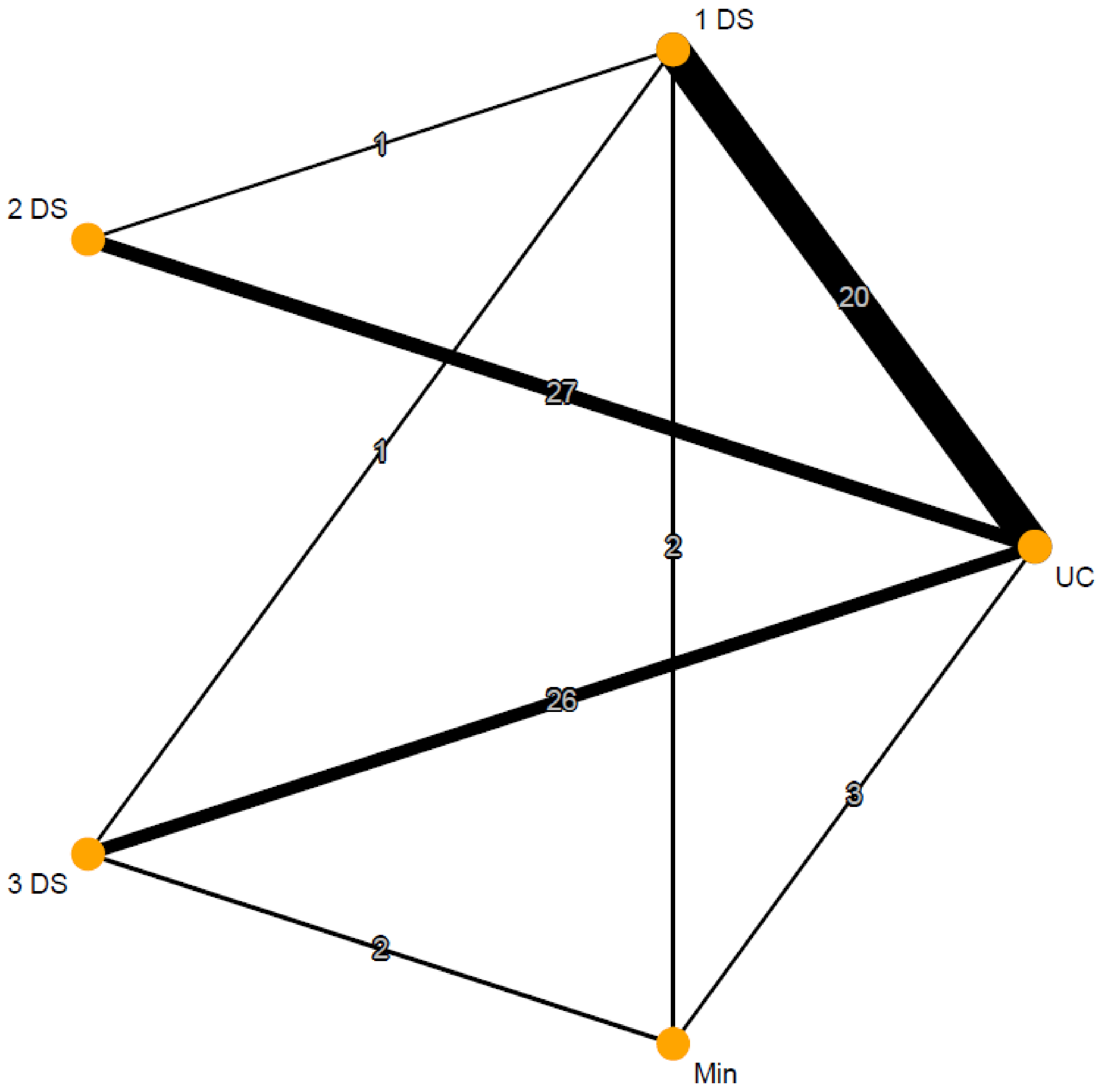

Network meta-analysis forest plot:

© 2023 Tyler N et al. *JAMA Network Open.*

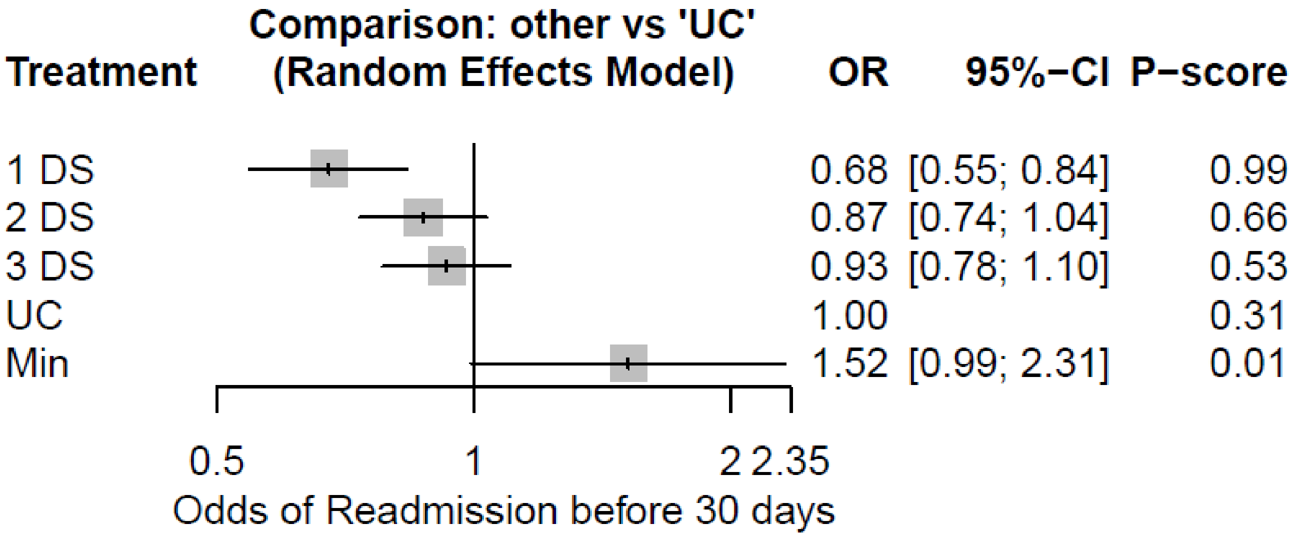

Heterogeneity:

I^2 = 65.5% [55.9%; 73.0%]

90-day readmission

- Discharge process

© 2023 Tyler N et al. *JAMA Network Open.*

Network Graph:

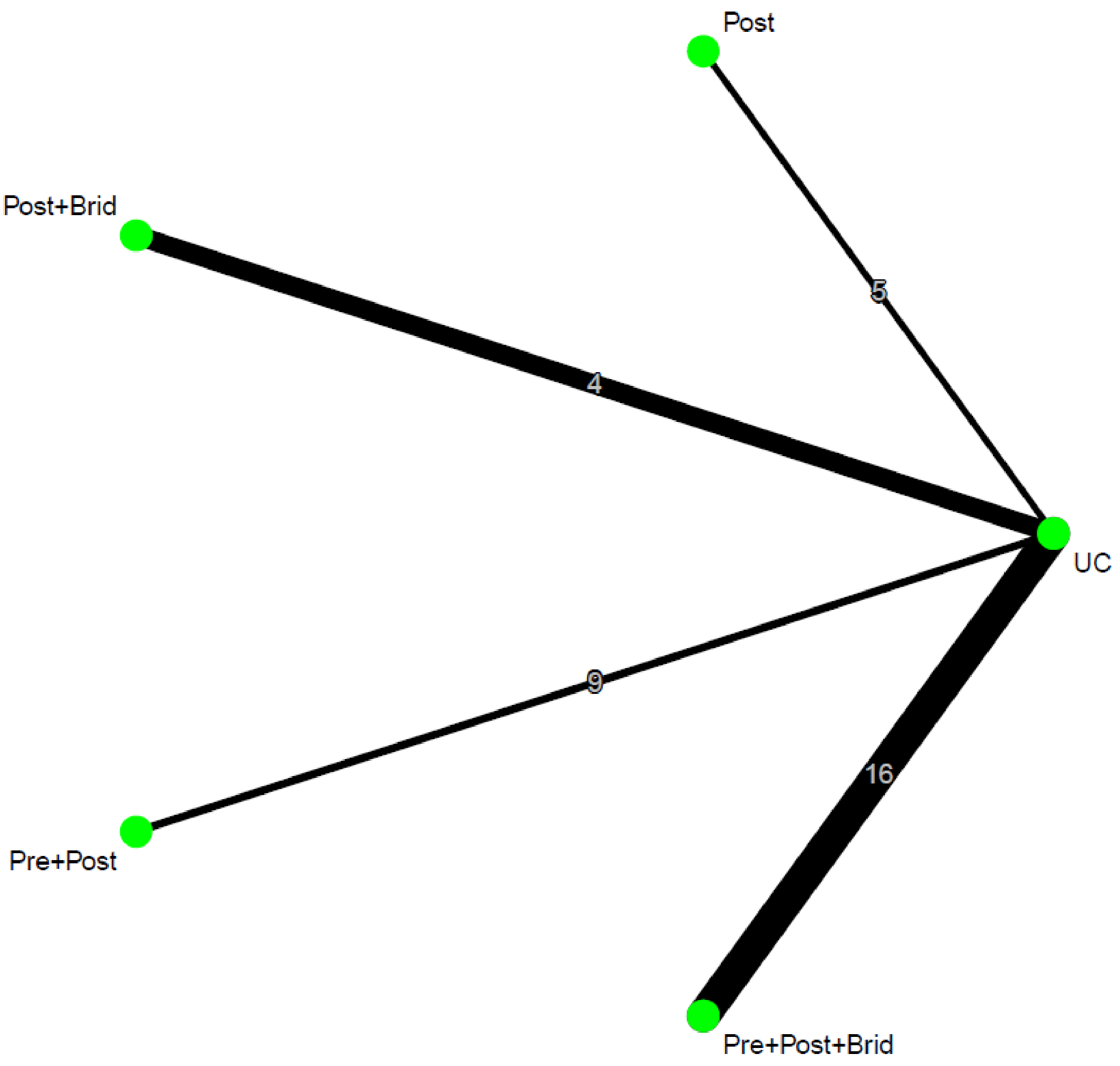

Network meta-analysis forest plot:

© 2023 Tyler N et al. *JAMA Network Open.*

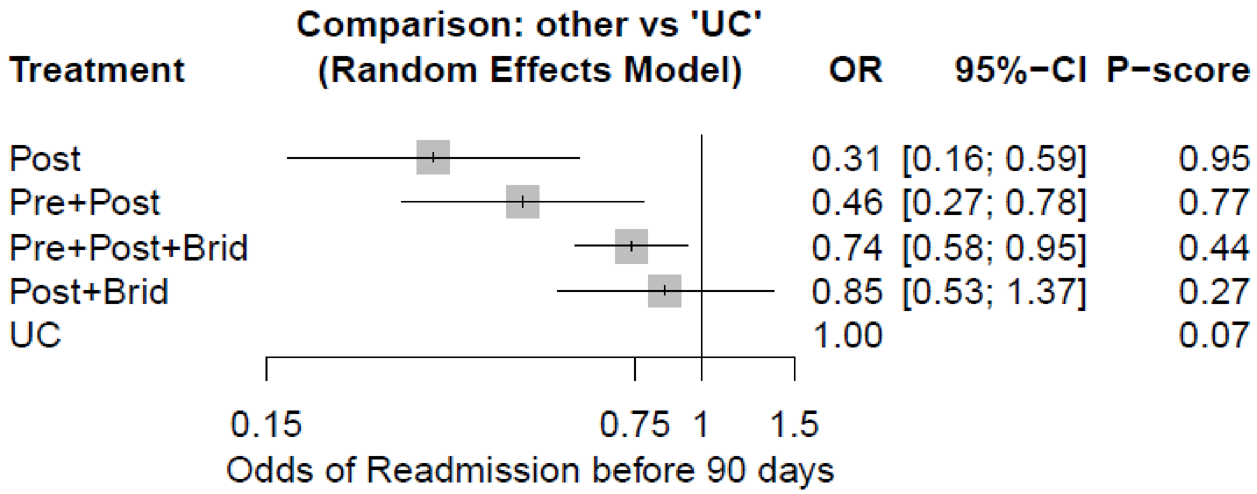

Heterogeneity:

I^2 = 63.4% [46.3%; 75.1%]

- Number of discharge stages

Network Graph:

© 2023 Tyler N et al. *JAMA Network Open.*

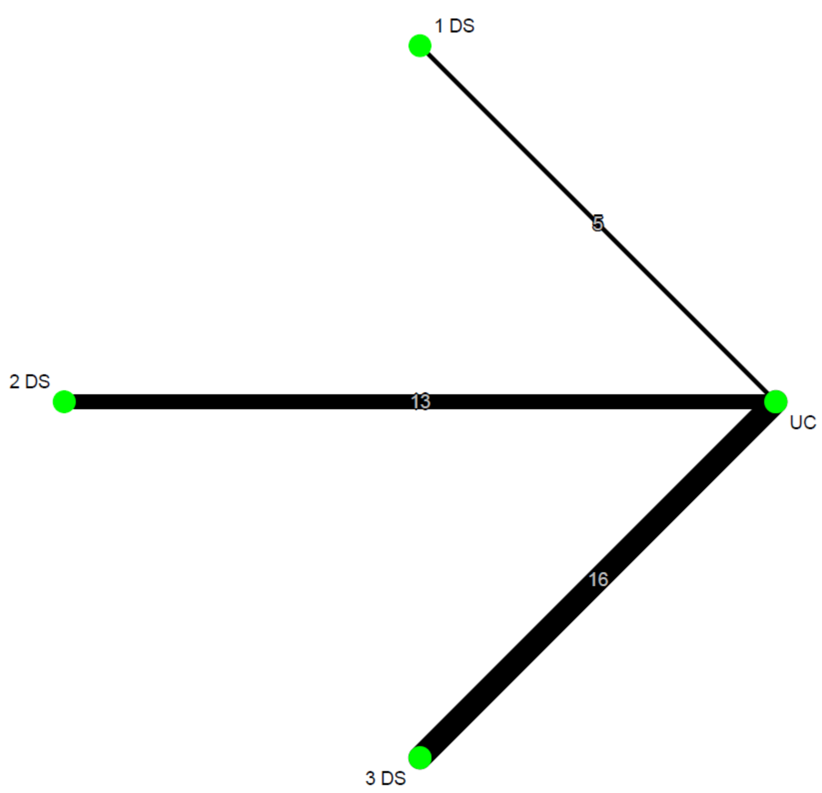

Network meta-analysis forest plot:

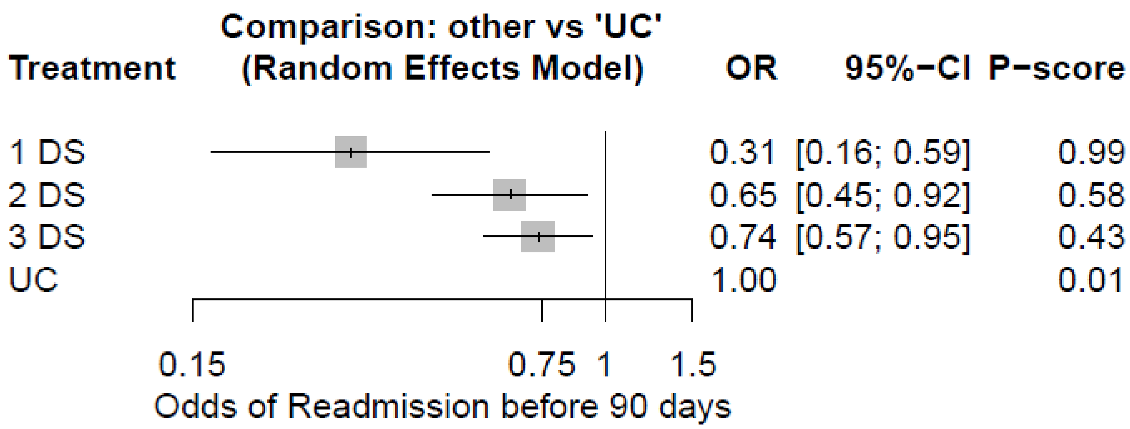

Heterogeneity:

I^2 = 64.6% [48.6%; 75.7%]

© 2023 Tyler N et al. *JAMA Network Open.*

Appendix 9: Readmissions and Secondary outcomes

30-day readmission

Network meta-analysis of 30-day readmissions with network graph and forest plot.

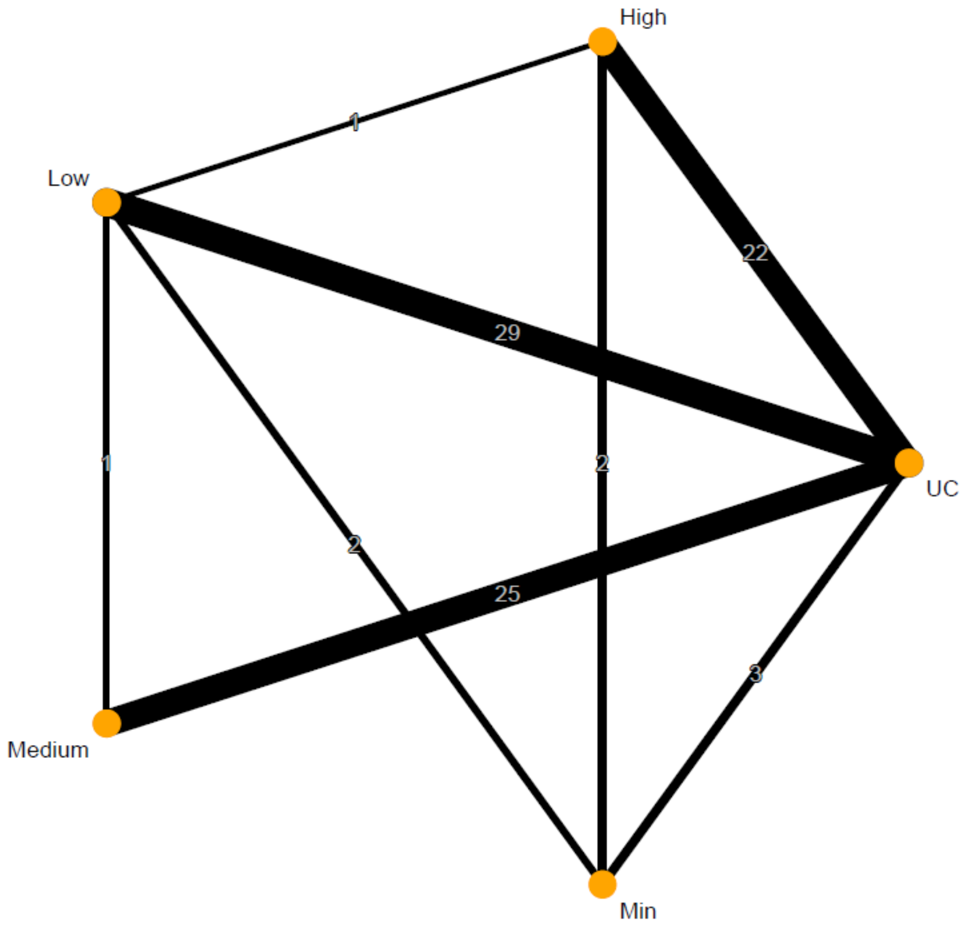

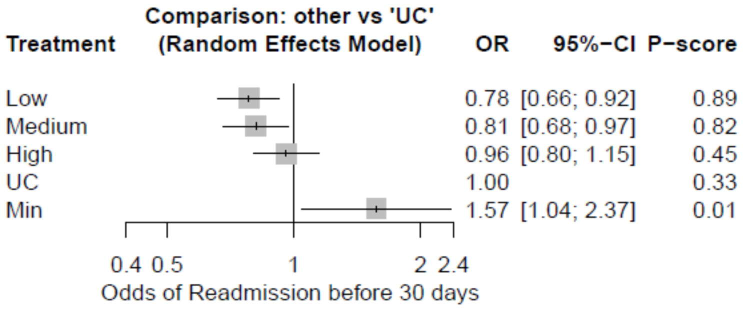

© 2023 Tyler N et al. *JAMA Network Open.*

90-day readmission

Network meta-analysis of 90-day readmissions with network graph and forest plot.

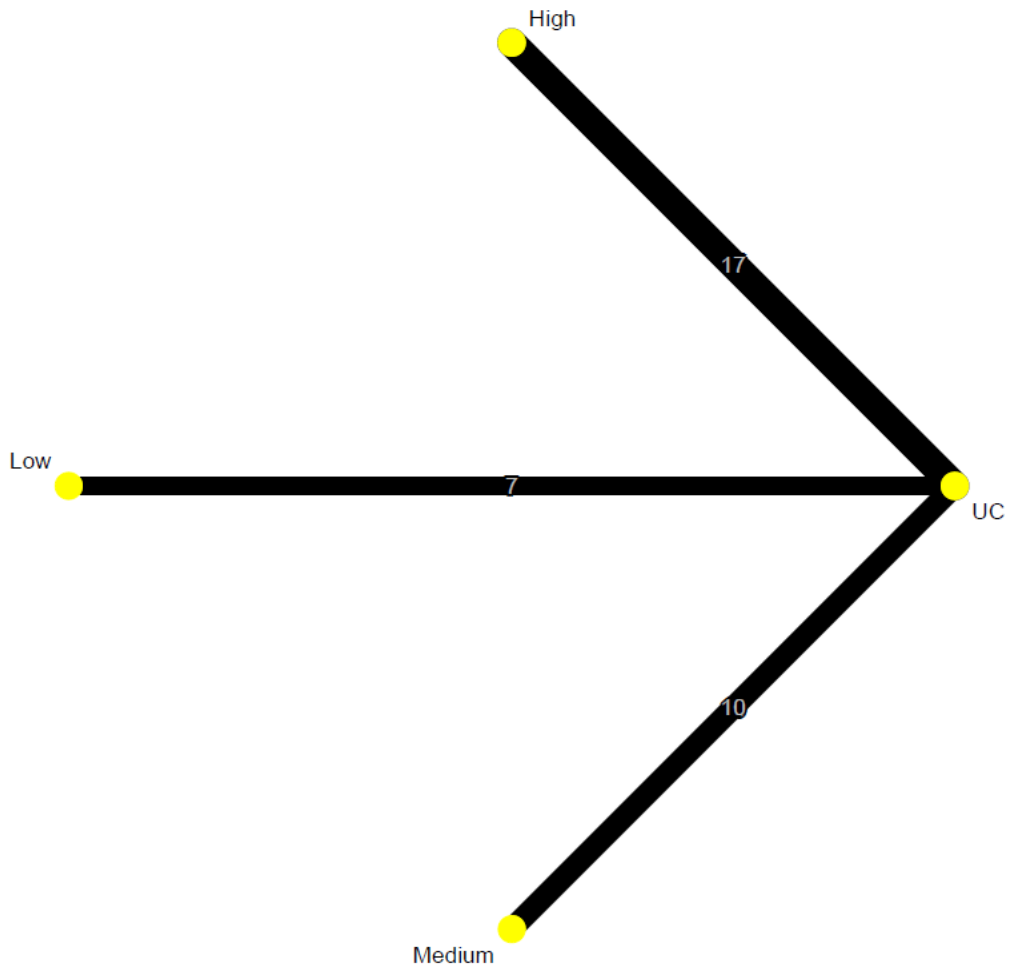

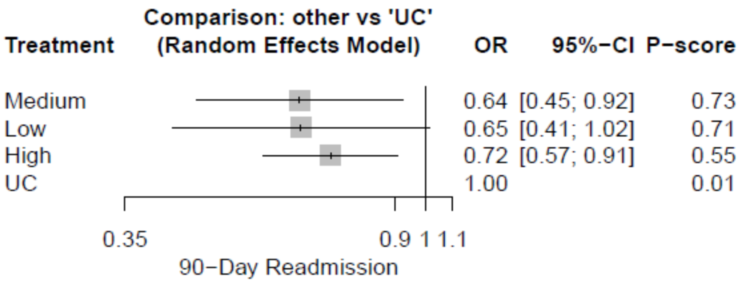

Pairwise meta-analysis of some secondary outcomes was done using Hartung-Knapp adjustment.

© 2023 Tyler N et al. *JAMA Network Open.*

180-day readmission

Network meta-analysis of 180-day readmissions with network graph and forest plot.

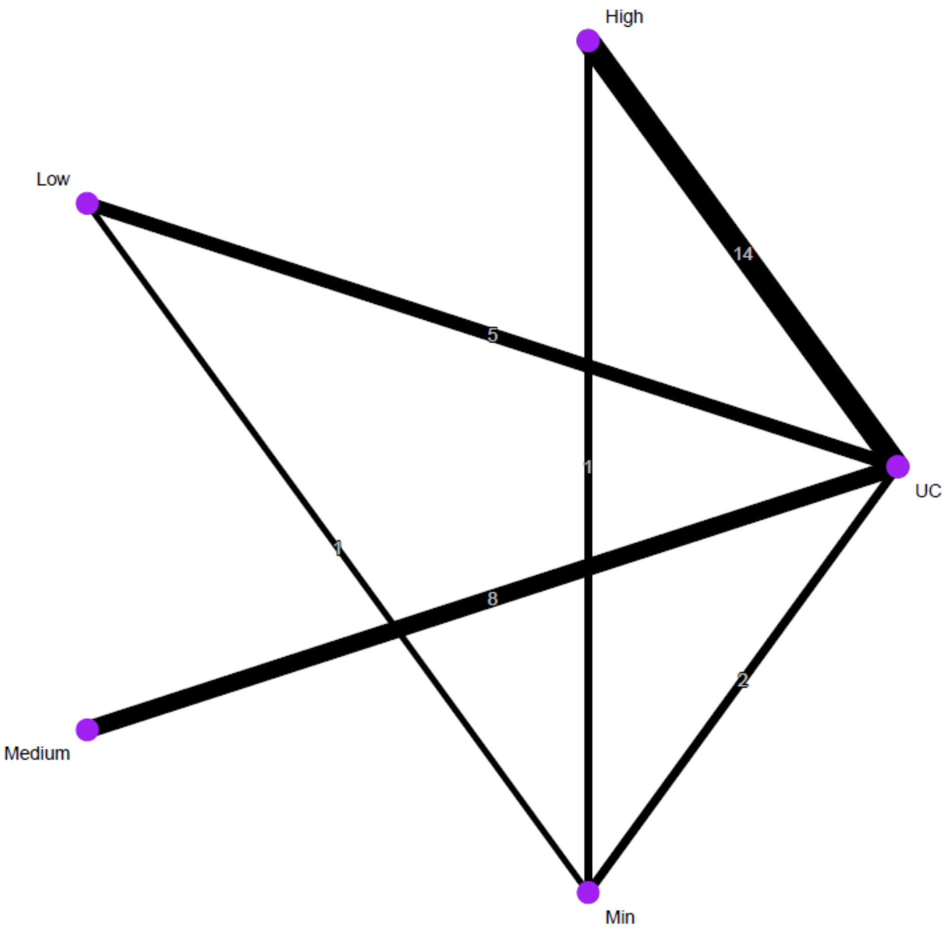

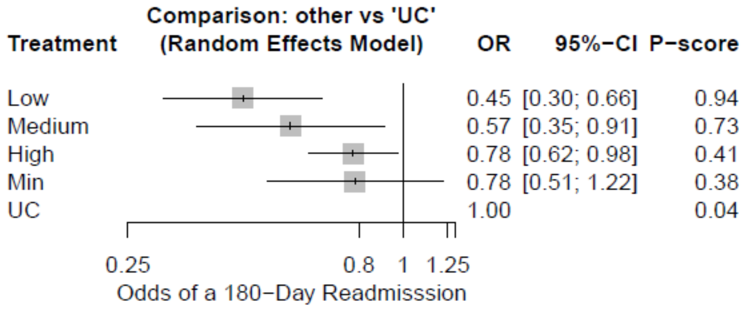

© 2023 Tyler N et al. *JAMA Network Open.*

Adverse events (Pairwise meta-analysis)

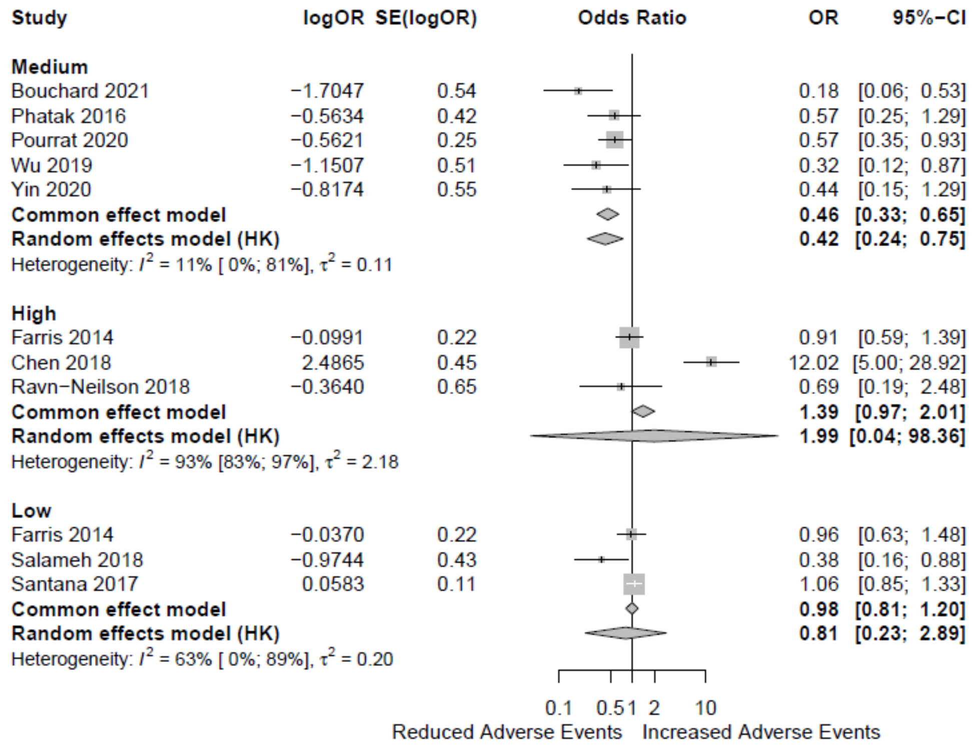

Footnote:

TE: log odds ratio; seTE: standard error logs odds ratio; CI: confidence interval

© 2023 Tyler N et al. *JAMA Network Open.*

Medication Adherence (Pairwise meta-analysis)

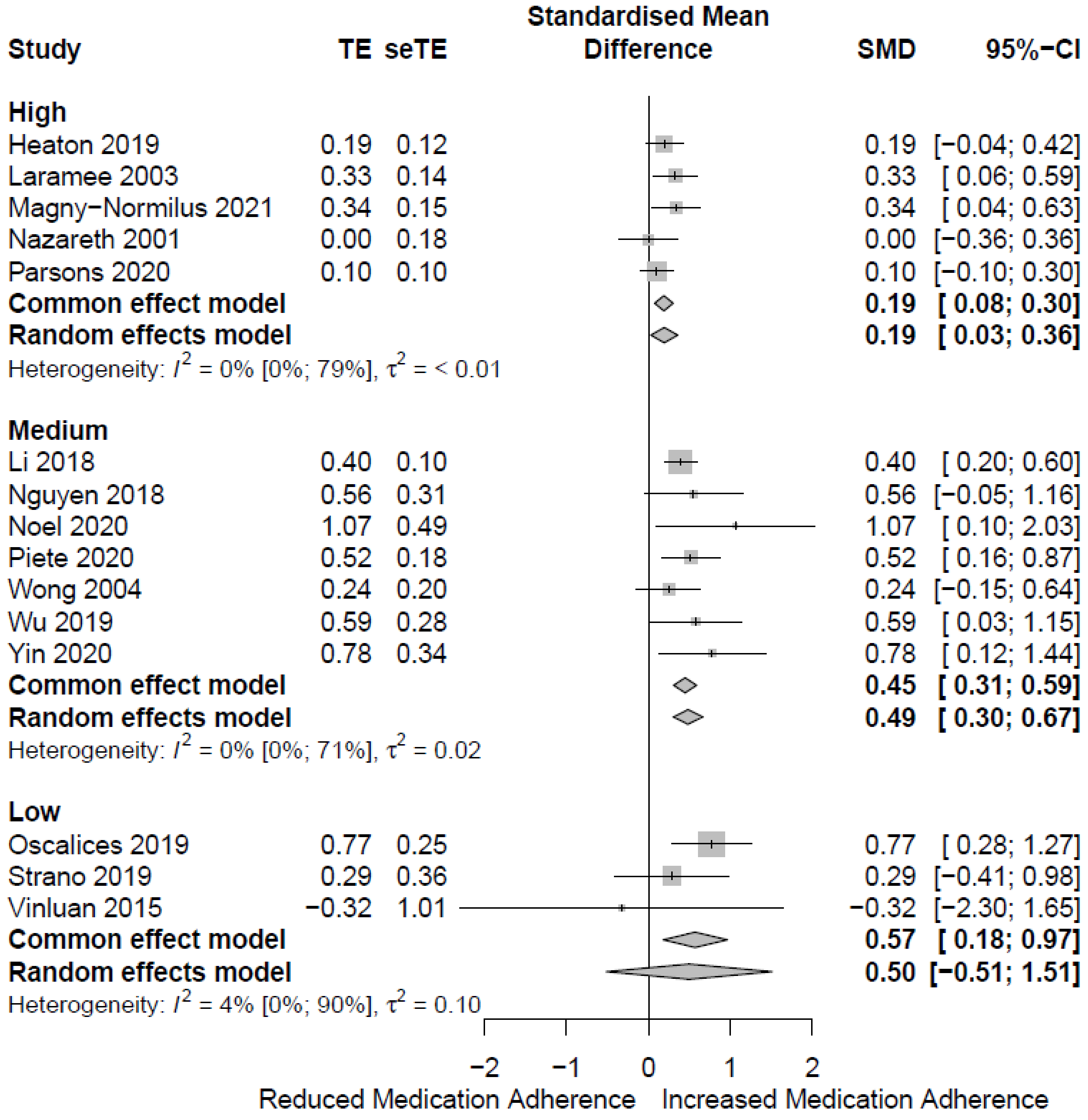

© 2023 Tyler N et al. *JAMA Network Open.*

Patient safety incidents (Pairwise meta-analysis)

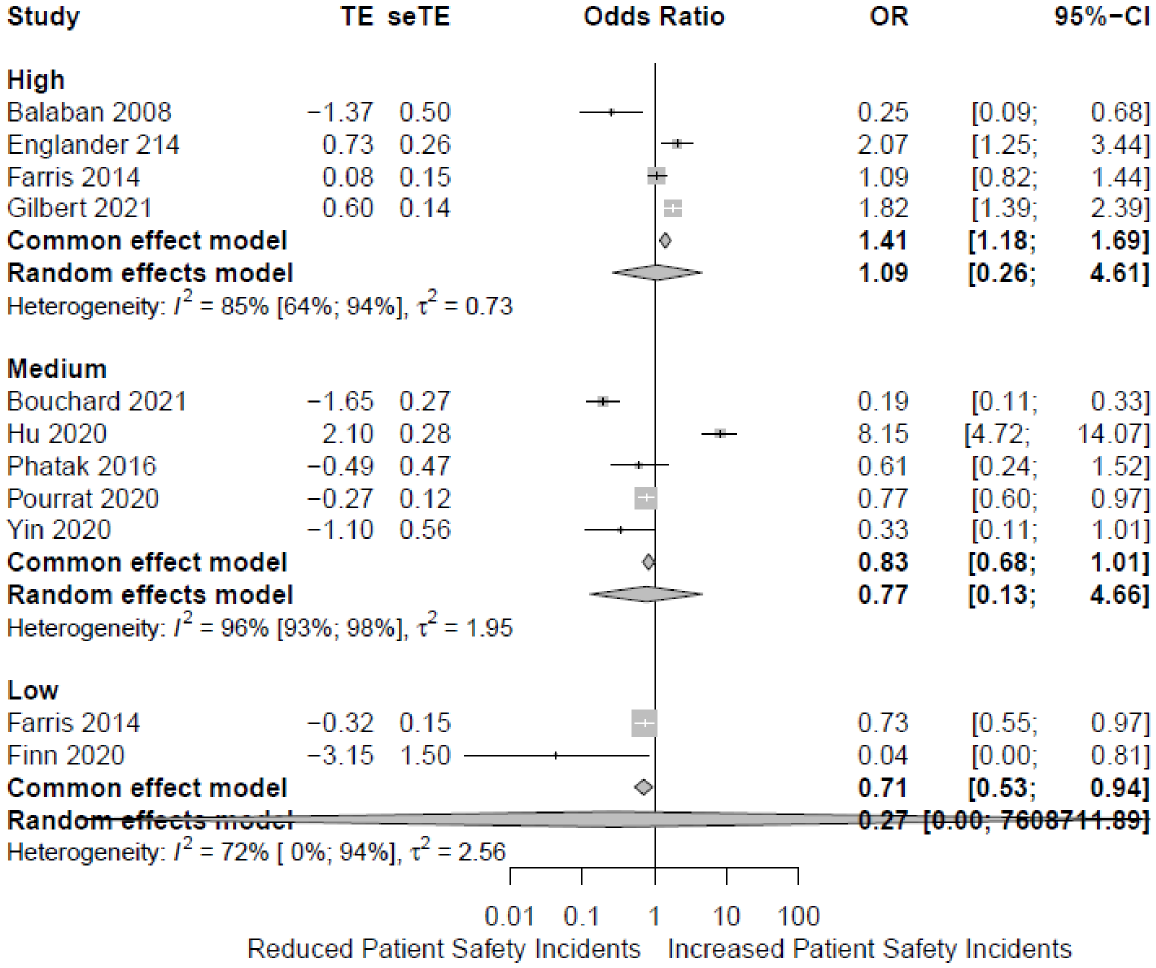

© 2023 Tyler N et al. *JAMA Network Open.*

ED visit

Network meta-analysis of ED visit with network graph and forest plot.

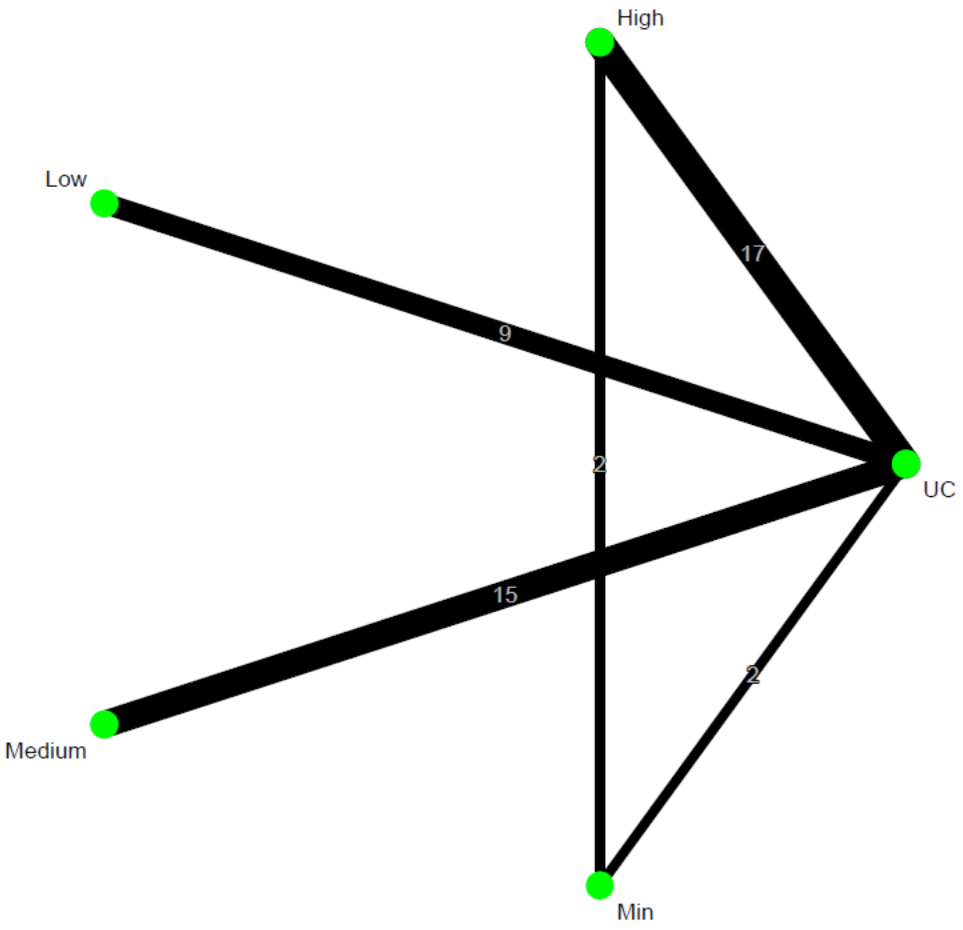

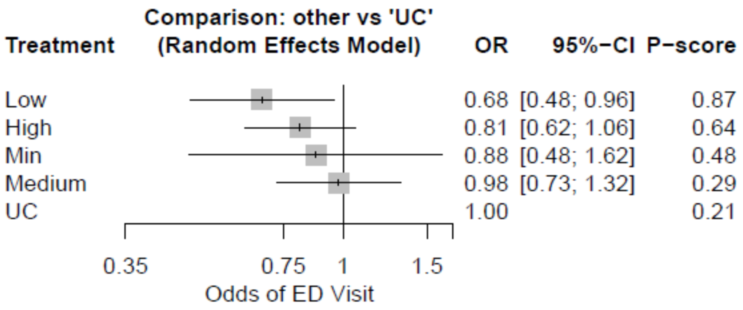

© 2023 Tyler N et al. *JAMA Network Open.*

Length of stay (Pairwise meta-analysis)

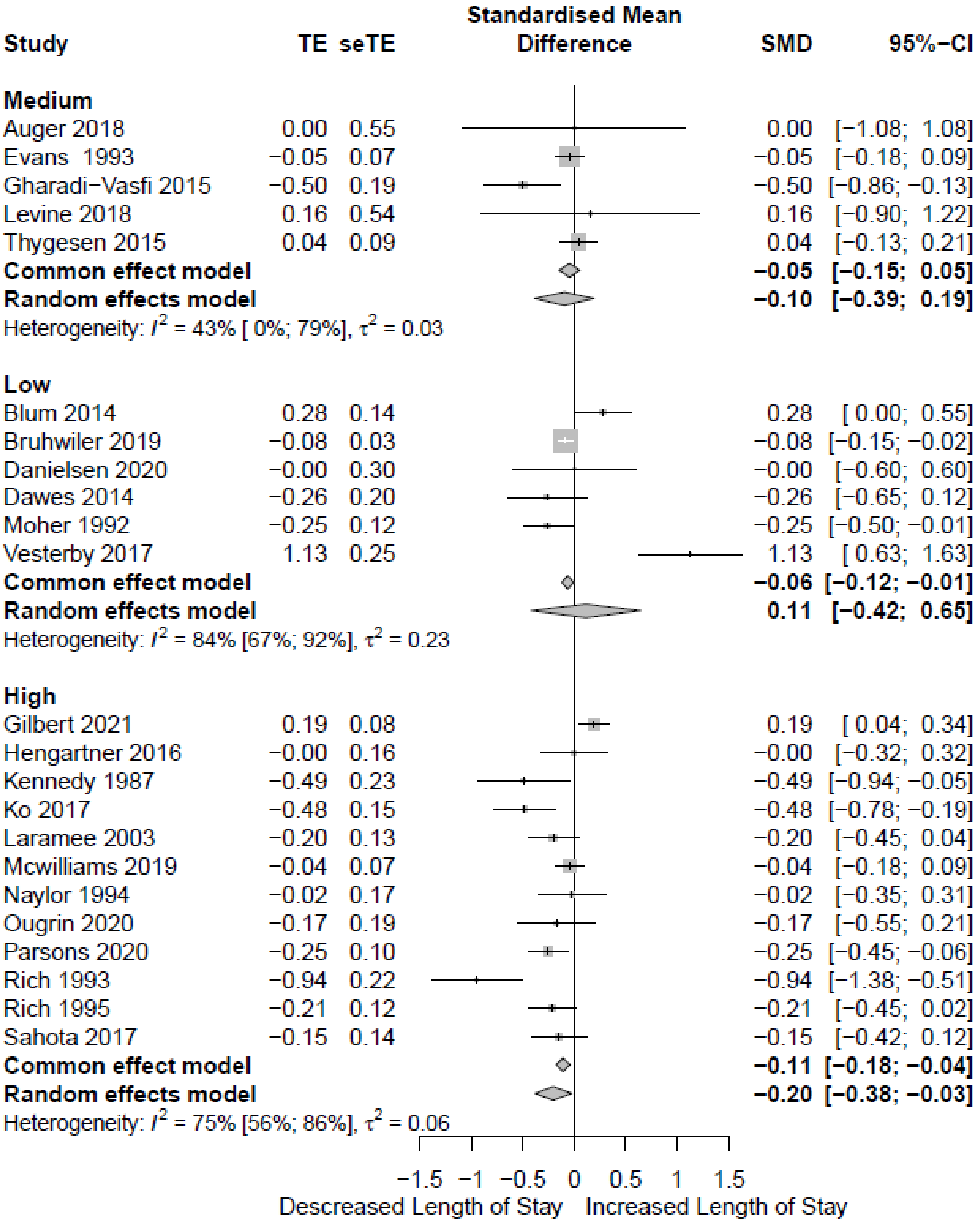

© 2023 Tyler N et al. *JAMA Network Open.*

Mortality (Network meta-analysis)

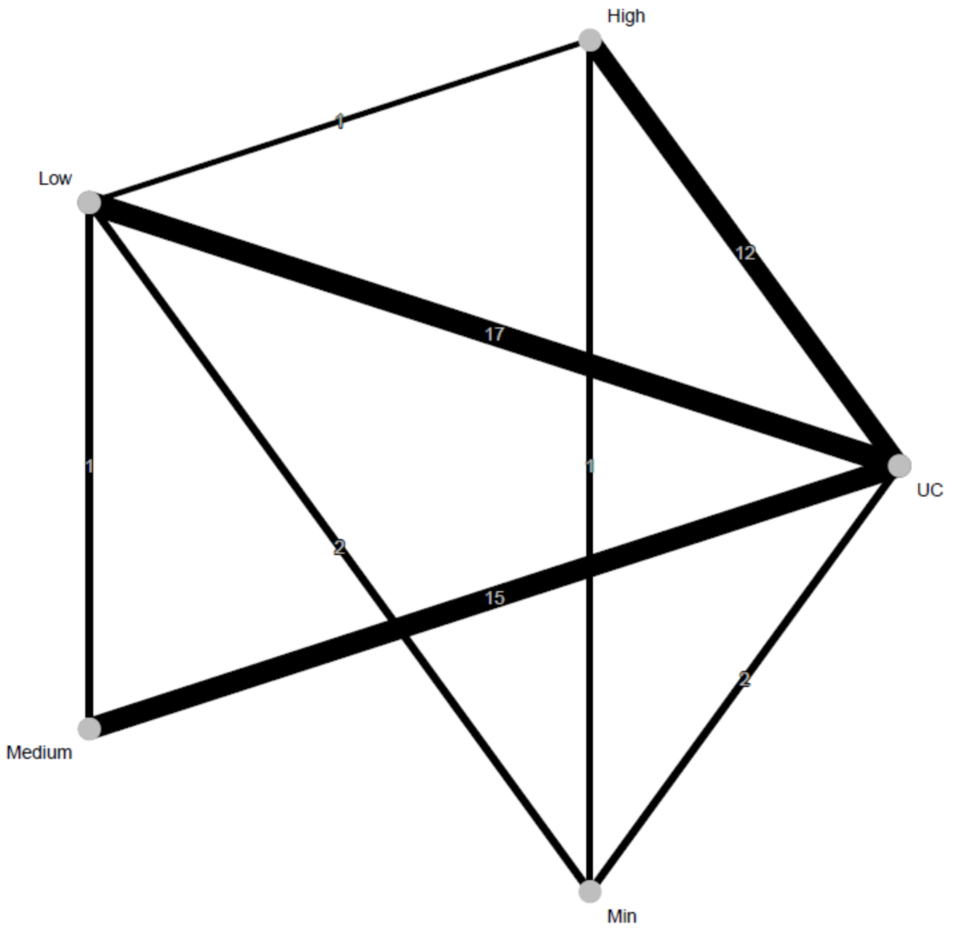

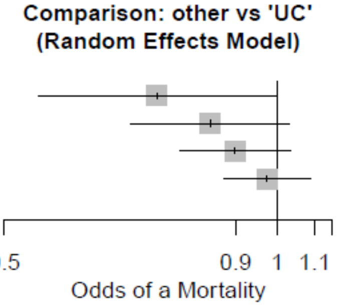

Treatment	OR	95%−CI	P−score
Min	0.74	[0.55; 1.00]	0.90
Medium	0.84	[0.69; 1.03]	0.69
Low	0.90	[0.78; 1.03]	0.54
High	0.97	[0.87; 1.09]	0.25
UC	1.00		0.12

© 2023 Tyler N et al. *JAMA Network Open.*

General QoL (Pairwise meta-analysis)

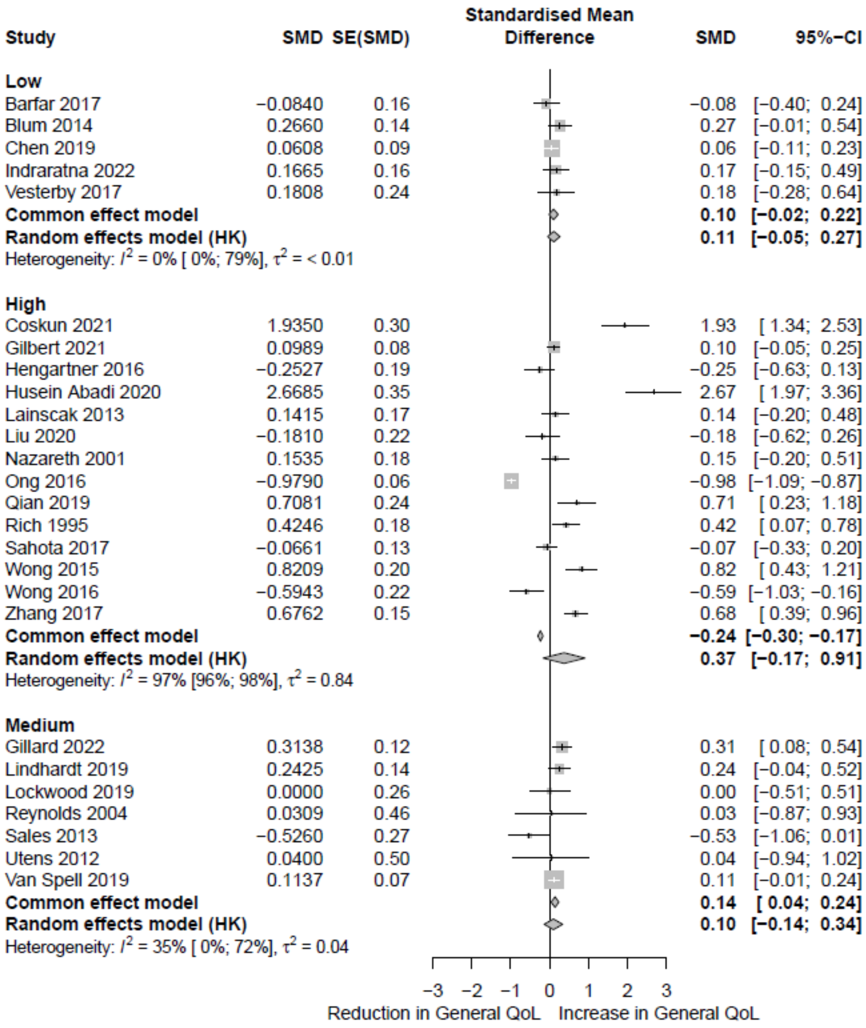

© 2023 Tyler N et al. *JAMA Network Open.*

Mental QoL (Pairwise meta-analysis)

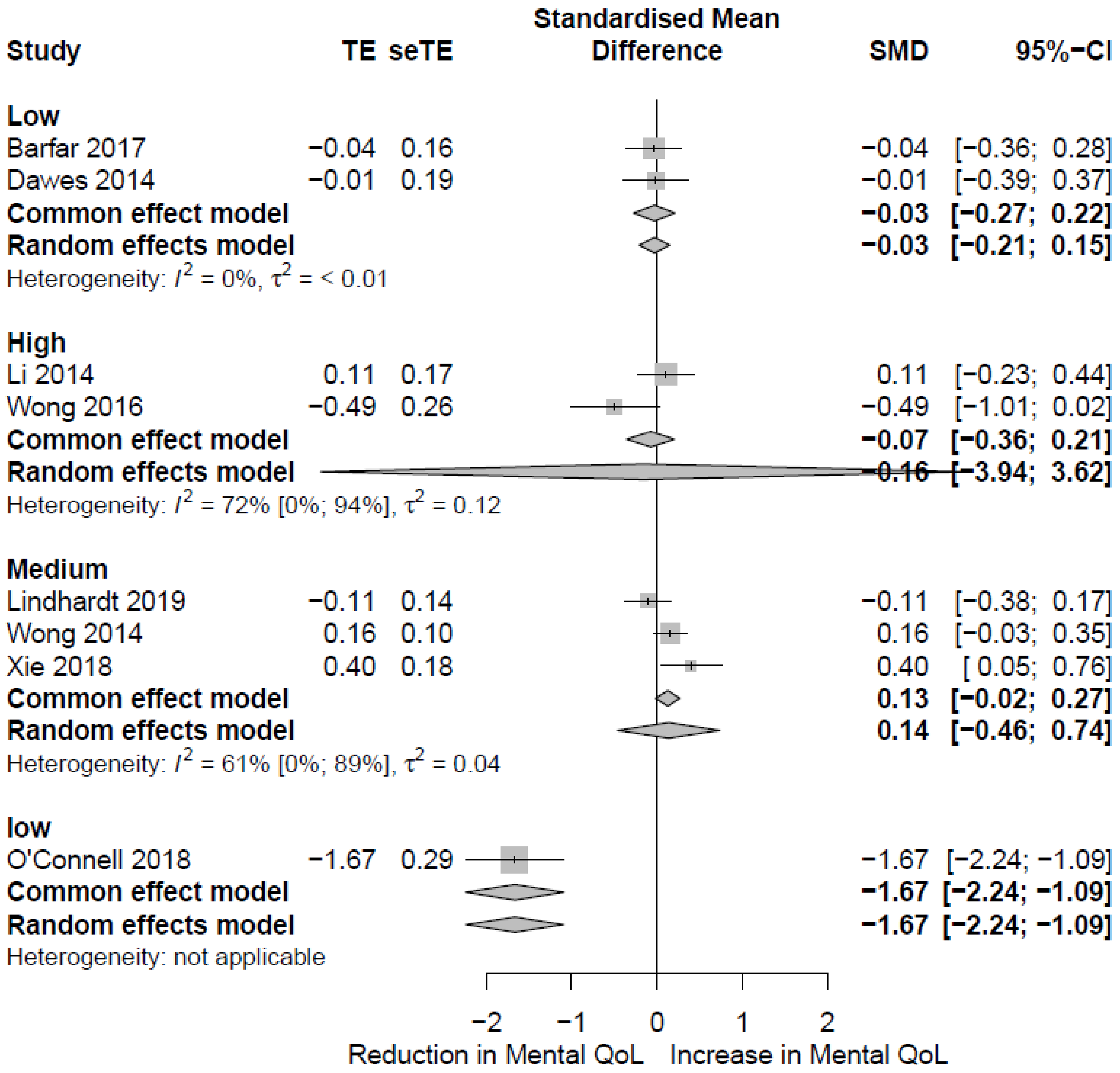

© 2023 Tyler N et al. *JAMA Network Open.*

Physical QoL (Pairwise meta-analysis)

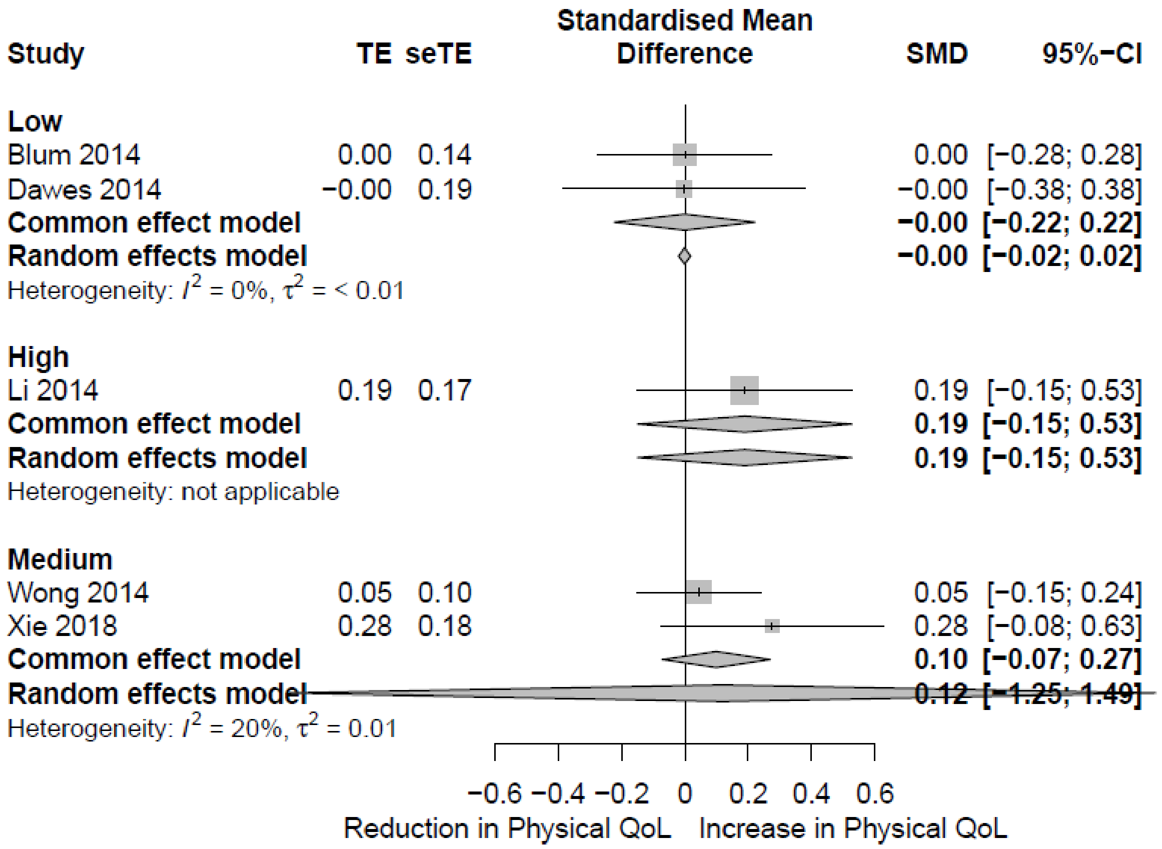

© 2023 Tyler N et al. *JAMA Network Open.*

Patient satisfaction (Pairwise meta-analysis)

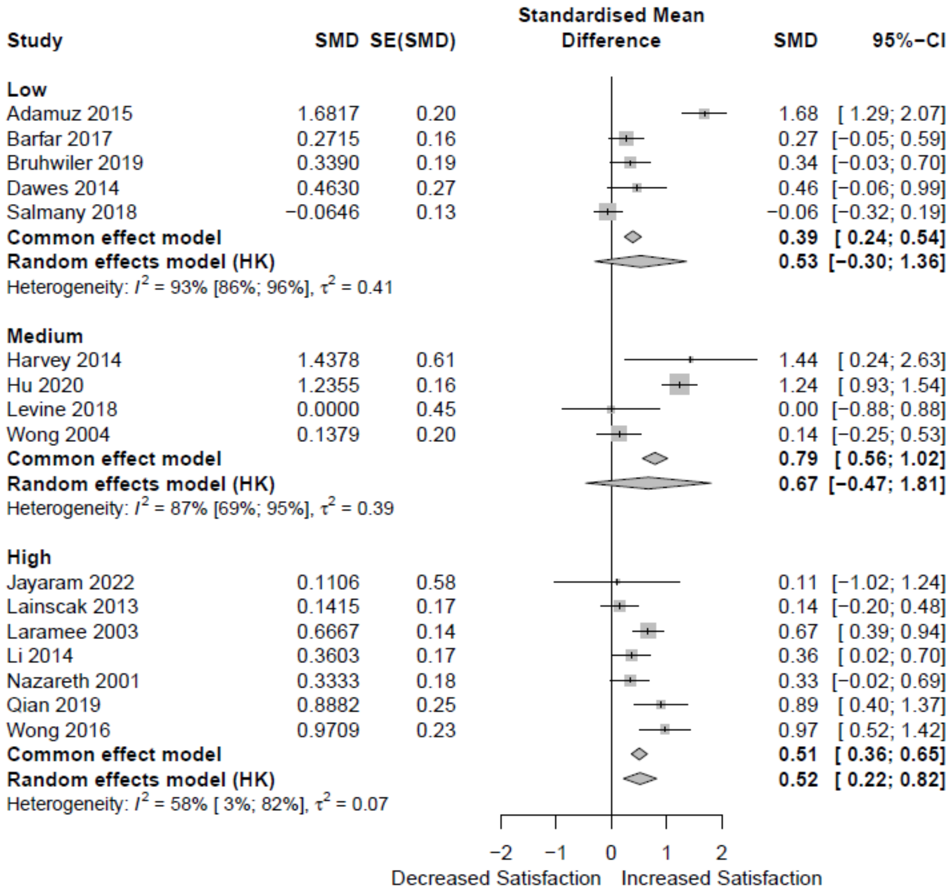

© 2023 Tyler N et al. *JAMA Network Open.*

Intervention Uptake (Network meta-analysis)

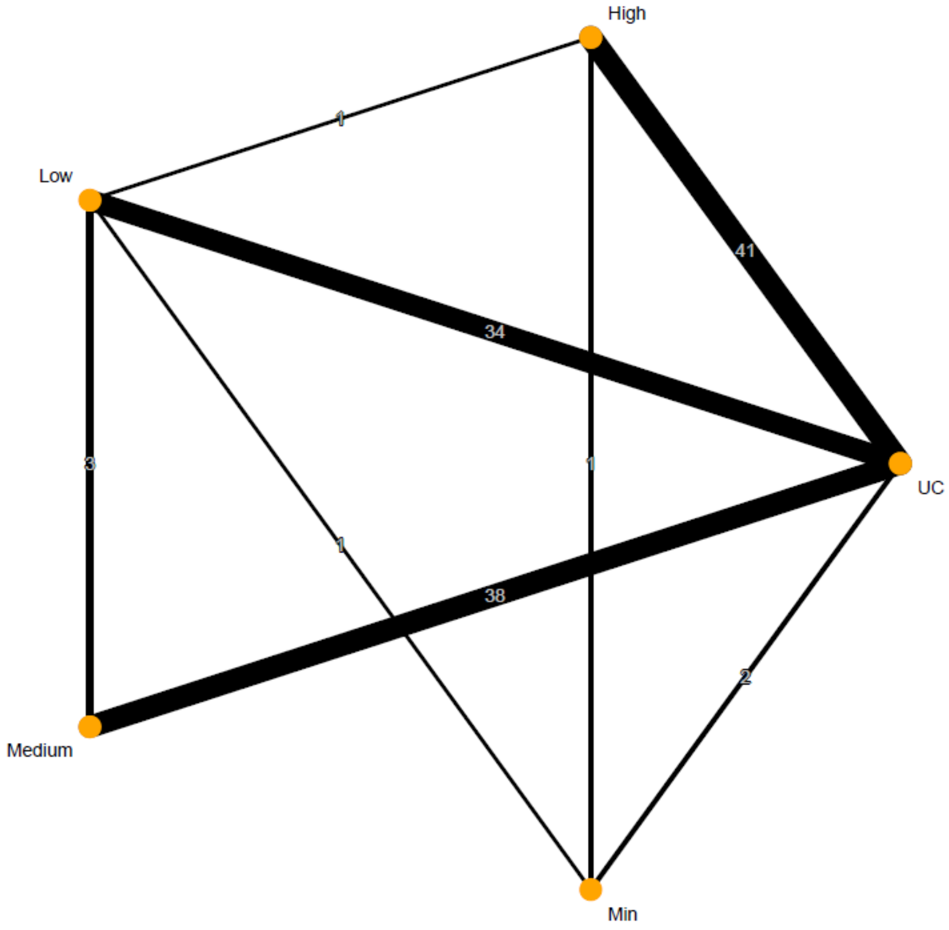

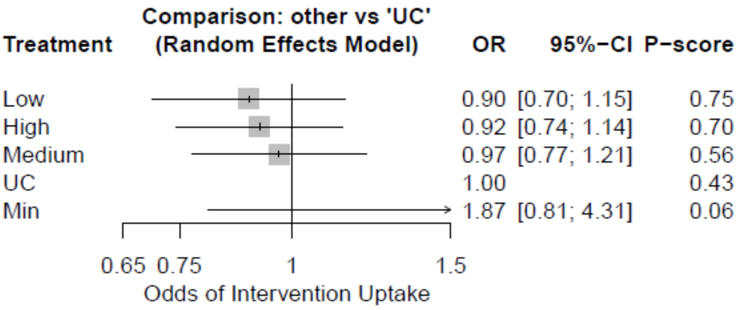

© 2023 Tyler N et al. *JAMA Network Open.*
