# Supplementary material for: Transitional Care Interventions From Hospital to Community to Reduce Health Care Use and Improve Patient Outcomes: A Systematic Review and Network Meta-Analysis
Source: JAMA Netw Open. 2023 Nov 30;6(11):e2344825. doi: 10.1001/jamanetworkopen.2023.44825 (PMC10690480; doi:10.1001/jamanetworkopen.2023.44825)
Supplement: Supplement 2. — Data Sharing Statement [file jamanetwopen-e2344825-s002.pdf]

Data Sharing Statement

Tyler. Transitional Care Interventions From Hospital to Community to Reduce Health Care Use and Improve Patient Outcomes. *JAMA Netw Open*. Published November 30, 2023. doi:10.1001/jamanetworkopen.2023.44825

Data
Data available: Yes
Data types: Study level aggregate data
How to access data: natasha.tyler@manchester.ac.uk
When available: With publication

Supporting Documents
Document types: None

Additional Information
Who can access the data: anyone requesting the data
Types of analyses: any purpose
Mechanisms of data availability: with investigator support
